# Dehydroborylation of Terminal Alkynes Using Lithium Aminoborohydrides

**DOI:** 10.3390/molecules28083433

**Published:** 2023-04-13

**Authors:** P. Veeraraghavan Ramachandran, Henry J. Hamann

**Affiliations:** Herbert C. Brown Center for Borane Research, Department of Chemistry, Purdue University, 560 Oval Drive, West Lafayette, IN 47907, USA

**Keywords:** amine-borane, dehydrogenative borylation, lithium aminoborohydrides, alkynylborane-amines, 1,1-dibromoolefin

## Abstract

Dehydrogenative borylation of terminal alkynes has recently emerged as an atom-economical one-step alternative to traditional alkyne borylation methodologies. Using lithium aminoborohydrides, formed in situ from the corresponding amine-boranes and *n*-butyllithium, a variety of aromatic and aliphatic terminal alkyne substrates were successfully borylated in high yield. The potential to form mono-, di-, and tri-*B*-alkynylated products has been shown, though the mono-product is primarily generated using the presented condition. The reaction has been demonstrated at large (up to 50 mmol) scale, and the products are stable to column chromatography as well as acidic and basic aqueous conditions. Alternately, the dehydroborylation can be achieved by treating alkynyllithiums with amine-boranes. In that respect, aldehydes can act as starting materials by conversion to the 1,1-dibromoolefin and in situ rearrangement to the lithium acetylide.

## 1. Introduction

**Scheme 1:**
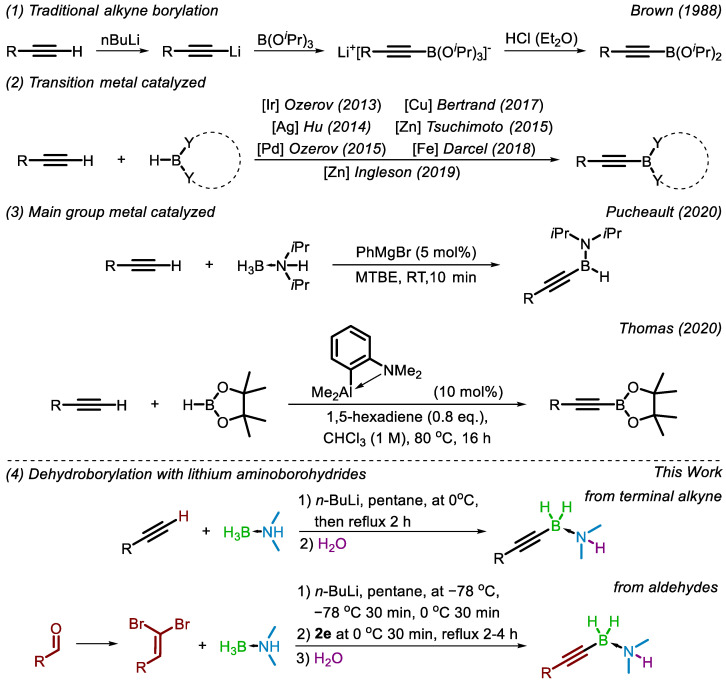
Past and present alkyne borylation methodologies; Brown [3], Ozerov [6], Hu [7], Ozerov [8], Bertrand [11], Tsuchimoto [9], Darcel [12], Ingleson [10], Pucheault [13], Thomas [14].

Alkynylboron compounds are valuable intermediates in organic synthesis and can be used as building blocks in a wide range of transformations [1], including coupling reactions [2]. Traditional synthetic routes to these compounds (Scheme 1(1)) involve conversion to an alkynylmetal, exchange with a boron source, and treatment with a dry Brønsted acid [3]. A more atom-economical approach to this transformation is a one-step, direct coupling between the terminal alkyne and the boron source, producing dihydrogen gas as a byproduct. Efforts towards this one-step, dehydrogenative borylation have been frustrated by the tendency of alkynes to undergo hydroboration rather than boronation upon reaction with dioxaborolane and diboron reagents [4,5]. A transition metal-catalyzed dehydrogenative borylation was first reported in 2013, utilizing an iridium SiNN pincer complex (Scheme 1(2)) [6]. Since that initial report, a variety of transition metals (Ag [7], Pd [8], Zn [9,10], Cu [11], Fe [12]) and ligand systems have been reported to accomplish the dehydrocoupling (Scheme 1(2)). However, many of these methodologies use expensive metal catalysts or intricate ligands.

Recently, several methods utilizing main group elements to catalyze the dehydrogenative borylation of terminal alkynes have been developed. Pucheault and coworkers employed magnesium halide (Grignard) catalysts to produce the corresponding acetylides from terminal alkynes [13]. Reaction with diisopropylaminoborane and tandem deprotonation by alkyne regenerated the magnesium acetylide and provided the dehydrocoupled diisopropylaminoalkynylborane product. Grignard promoted in situ dehydrogenation of diisopropylamine-borane to form diisopropylaminoborane was found to be an equally effective starting point for the dehydrocoupling (Scheme 1(3)).

An aluminum-catalyzed borylation of terminal alkynes reported by Thomas and coworkers utilizes the intramolecular alane–amine complex formed from the reaction of 2-lithio-*N,N*-dimethylaniline with Me_2_AlCl [14]. The tethered Lewis pair catalyzes the formation of an alkynyl aluminum intermediate, which yields the desired alkynylboronate upon reaction with pinacol borane (Scheme 1(3)). While the desired boronate is provided, the highly reactive nature of aluminum compounds makes their handling difficult.

Hydrometallation was a concern for both the magnesium and aluminum catalyzed dehydrocoupling reactions. It was found in each case that by producing an alkynylmetal intermediate, hydrometallation could be circumvented. As part of our work exploring the synthetic utility of amine-boranes [15,16] we have recently discovered a direct C-H dehydrogenative borylation of terminal alkynes. Lithium aminoborohydrides (LABs) generated from air- and moisture-stable amine-borane precursors have been found to be effective reagents for the dehydroborylation (Scheme 1(4)).

## 2. Results and Discussion

**Scheme 2:**
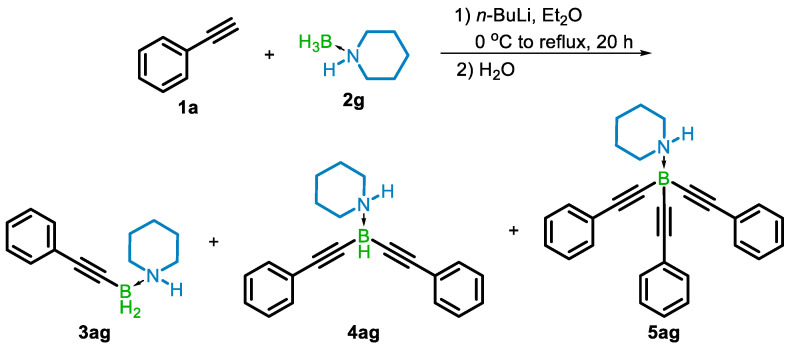
Initially obtained mono-, di- and trialkynylated amine-boranes.

LAB reagents are generated from the reaction of an amine-borane with n-butylithium (n-BuLi) and are best known for their powerful and selective reducing properties, similar to lithium aluminum hydride [17]. When prepared with a slight deficit of n-BuLi, LAB reagents are air-stable solids. Initially, we were investigating LAB reagents as activated derivatives of amine-boranes for the hydroboration of alkynes, as most amine-boranes themselves do not hydroborate under ambient conditions [18,19]. A reaction of phenylacetylene (**1a**) and lithium piperidinoborohydride prepared from piperidine-borane (**2g**) was found to provide a series of alkynylated amine-boranes, not the hydroboration product (Scheme 2).

The formation of the mono- (**3ag**), di- (**4ag**), and tri-*B*-alkynylated (**5ag**) amine-borane was confirmed by ^11^B NMR and HRMS (see Supporting Information). This preliminary experiment demonstrated the potential of LABs to undergo dehydrocoupling with up to 3 terminal alkynes. With this result, we set about standardizing the reaction conditions, starting with the order of addition. Using 4-methoxyphenylacetylene (**1b**) and dimethylamine-borane (DMAB) (**2e**), three experiments were performed in diethyl ether (Et_2_O): (1) the LAB reagent was formed using **2e** and *n*-BuLi, followed by addition of **1b**, (2) the lithium acetylide was formed using **1b** and *n*-BuLi followed by addition of **2e**, and (3) *n*-BuLi was added to a mixture of **1b** and **2e**. In each case the formation of lithium *N,N*-dimethylaminoborohydride was detected by ^11^B NMR (δ −14.88 (q, *J* = 83.4 Hz)), and the total conversion of **1b** to one of the alkynylborane products after 24 h at room temperature was similar for each equivalence examined (see Supporting Information). For operational simplicity, method 3 was used going forward to further examine the reagent equivalence, where it was found that utilizing **1b** and **2e** in a ratio of 1 to 2, with 1.865 eq. of *n*-BuLi provided nearly quantitative conversion of the alkyne, with a favorable 85:15 ratio of mono-*B*-substituted (**3be**) product to the di-*B*-substituted (**4be**) product (see Supporting Information). Using this stoichiometry, none of the tri-*B*-substituted (**5be**) product was detected. It is proposed that the excess of amine-borane helps to limit the formation of **4** and **5**, while the modest deficiency of *n*-BuLi prevents the concurrent formation of the lithium acetylide and LAB reagent.

Shortening the reaction duration increased the proportion of mono-B-substituted (**3be**) product at the expense of overall alkyne conversion, indicating a competition between the lithium alkynylaminoborohydride intermediate and the still-present LAB reagent for the remaining alkyne substrate. The formation of di-B-substituted product was minimized during the solvent study. Dictated by the reactivity of the LAB reagent, a series of primarily ethereal and hydrocarbon solvents were examined (Table 1 entries 1–7). When hydrocarbon solvents were employed, the intermediate product was visibly less soluble and the formation of the **4be** product was almost entirely suppressed. This led to the production of **3be** in both toluene and pentane with a total alkyne conversion of 79% and 92%, respectively. Fine-tuning of the reaction conditions (see Supporting Information) revealed that in refluxing pentane, the reaction was complete within 2 h, with a total alkyne conversion of 97% and a 99:1 ratio of **3be** to **4be** (Table 1 Entry 8).

Following the determination of the optimal reaction solvent and conditions, an investigation of the amine-borane reagent (**2a**–**2j**) was undertaken. The amine-boranes examined were prepared using either a salt metathesis [20] (**2a** and **2e**) or a sodium bicarbonate-mediated [21] (**2b**–**2d**, **2f**–**2j**) reaction. Fortuitously, **2e** used in the earlier investigations showed the greatest total alkyne conversion. Unhindered primary amine-boranes **2a** and **2b** showed only trace conversion, while bulkier **2c** and **2d** gave 16% and 82% conversion, respectively. Similar results were obtained for the other secondary amine-boranes examined. Diethylamine- (**2f**) and piperidine-borane (**2g**) gave 66% and 83% conversion respectively, but morpholine-borane (**2h**) gave only trace product. The highly hindered diisopropylamine-borane (**2i**) gave only 14% conversion, in line with what was observed previously for a similar reaction using **2i** [13]. The complete absence of product when using triethylamine-borane (**2j**) bolsters the proposed importance of the formation of the LAB reagent as the active intermediate in the reaction.

**Figure 1:**
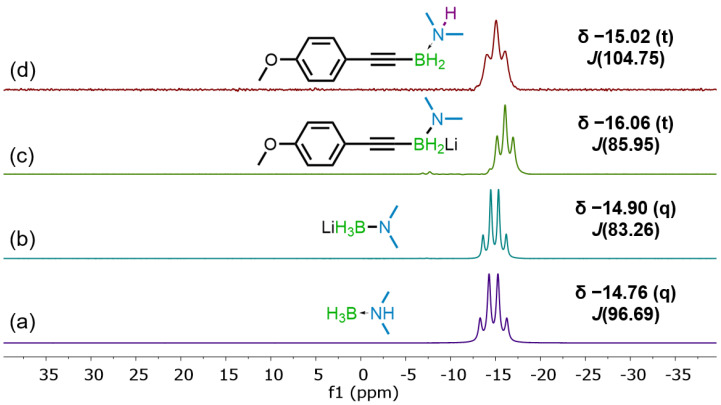
^11^B NMR peaks observed during reaction monitoring experiment; (**a**) dimethylamine-borane starting material, (**b**) lithium dimethylaminoborohydride after *n*-BuLi addition, (**c**) lithium dimethylaminoalkynylborohydride following dehydroborylation, (**d**) final product obtained after water quench.

The LAB intermediate was additionally observed during an experiment tracking the reaction of **1b** and **2e** with ^11^B NMR spectroscopy (Figure 1). The initial quartet at δ −14.76 ppm consistent with **2e** (Figure 1a), was determined to have converted to the corresponding LAB reagent based on the coupling constant (*J* = 96.69 Hz vs. 83.26 Hz) (Figure 1b). Reaction of the LAB reagent with **1b** then produced a lithium dimethylaminoalkynylborohydride, represented by a triplet near δ −16 ppm (Figure 1c). Following the water quench, the product **3be** was detected as a triplet near δ −15 ppm (Figure 1d).

**Scheme 3:**
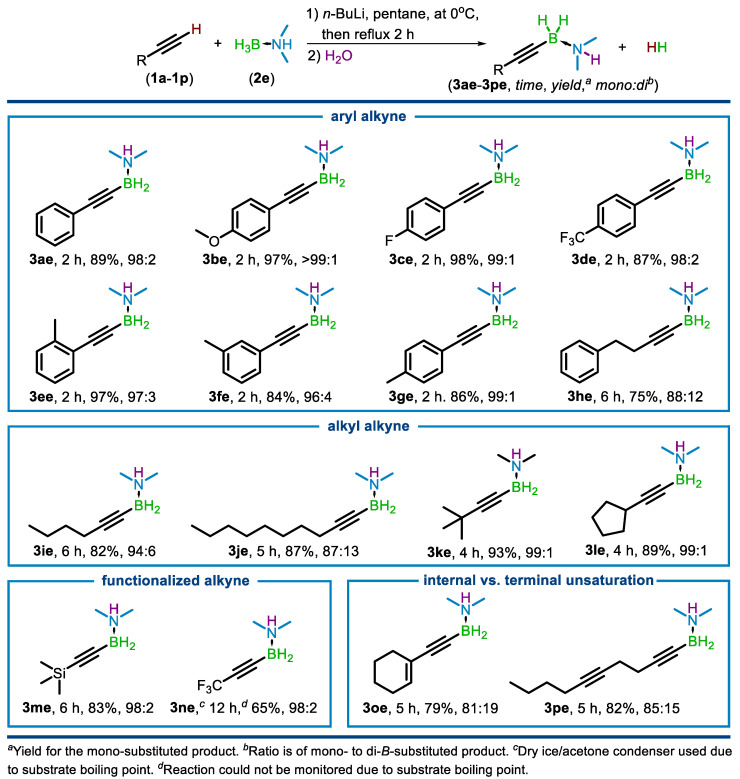
Scope of terminal alkyne substrates.

Using the optimized conditions for **2e**, the substrate scope was examined (Scheme 3). Aromatic terminal alkynes readily underwent the dehydrocoupling, including those with electron donating groups (**1b**), electron withdrawing groups (**1c** and **1d**), and those with substitutions at the ortho (**1e**), meta (**1f**), and para (**1g**) positions. Aromatic homologues (**1h**), as well as straight chain (**1i** and **1j**), branched (**1k**), and cyclic (**1l**) aliphatic terminals alkynes, were also amenable to borylation. The protocol was also applied to functionalized alkynes, including trimethylsilyl- (**1m**), and trifluromethylacetylene (**1n**). Selectivity for borylation at only the terminal alkyne position was demonstrated by the successful reactions of substrates with an internal alkene (**1o**) and internal alkyne (**1p**). The reactions of aryl alkynes were completed within 2 h, while alkyl alkyne substrate required up to 6 h for completion. The mono-*B*-substituted to di-*B*-substituted product ratio was greater than 80:20 for all substrates, though most substrates provided a ratio of 95:5 or greater. Reactions with substrates containing reducible groups or those that could be easily deprotonated were not successful, including esters, nitriles, alcohols, amines, and nitro groups.

**Scheme 4:**
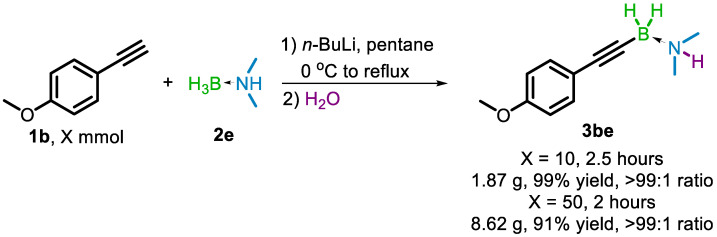
Gram-scale experiments for the synthesis of **3be**.

Following examination of the scope of usable terminal alkyne substrates, a series of gram-scale reactions were performed using 4-methoxyphenylacetylene (**1b**) and **2e**. Reactions at 10 mmol and 50 mmol scale with respect to **1b** each gave results in line with the 1 mmol reaction. Within 2–2.5 h, yields of 99% and 91% were obtained for the 10 mmol and 50 mmol scale reactions, respectively (Scheme 4). To circumvent the large-scale chromatographic separation of the product **3be** from the excess remaining **2e**, an aqueous workup procedure was used. It was found that **2e** would decompose into water-soluble products in the presence of dilute (1 M) HCl, while **3be** would remain intact, allowing it to be extracted using ethyl acetate. Basic aqueous solutions (1 M NaOH) were also tolerated by **3be**.

**Scheme 5:**
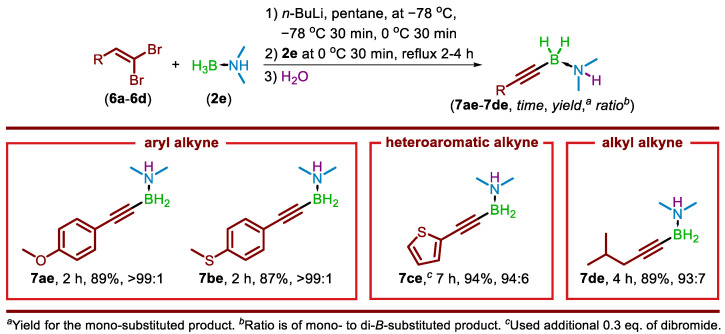
Alkynylborane-amines produced from 1,1-dibromoolefins.

To expand the scope of available terminal alkyne substrates, a modified method was envisioned. Terminal alkynes can be produced from aldehydes by reaction with a triphenylphosphine-dibromomethylene ylide formed from carbon tetrabromide and triphenylphosphine to yield a 1,1-dibromoolefin. Reaction of the dibromoolefin intermediate with *n*-BuLi yields the terminal alkyne, with the overall process known as the Corey–Fuchs reaction [22]. By isolating the 1,1-dibromoolefin intermediate and using that as the starting material, the Fritsch–Buttenberg–Wiechell rearrangement [23,24,25] would be performed in situ, and the lithium acetylide generated would go on to provide the borylated alkyne upon reaction with **2e**. To examine this potential alternative route to the alkynylborane-amine products, **3be** was selected as a target to have a direct comparison between the results of two methods. Using 4-methoxybenzaldehyde, 1-(2,2-dibromovinyl)-4-methoxybenzene (**6a**) was prepared using a reported procedure [22]. An initial reaction maintaining a **6a** to **2e** ratio of 1 to 2, while increasing the *n*-BuLi equiv. to 3.865, provided only 10% of the desired **7ae**. This low yield is attributed to the presence of excess *n*-BuLi, allowing both LAB reagent and lithium acetylide to be present in the reaction. Refinement of the reaction stoichiometry (see Supporting Information) led to an optimal **6a** to **2e** to *n*-BuLi ratio of 1:2:2.3. These conditions afforded **7ae** in 89% yield with a mono- to diborylated product ratio ≥99:≤1. Following this success, a series of 1,1-dibromoolefins (**6b**–**6d**) were prepared from 4-(methylthio)benzaldehyde, thiophene-2-carbaldehyde, and isovaleraldehyde. Substrates **6b**–**6d** were then subjected to the optimized conditions, and provided the corresponding alkynylated borane-amines **7be**, **7ce**, and **7de** in 87%, 99%, and 89%, respectively, demonstrating the compatibility of aryl, heteroaromatic, and alkyl 1,1-dibromoolefins with the devised protocol (Scheme 5).

## 3. Materials and Methods

### 3.1. General Information

All reagents and starting materials were purchased from Sigma-Aldrich (St. Louis, MO, USA), Oakwood (Estill, SC, USA) or Fisher Scientific (Waltham, MA, USA). Amines, ammonium salts, sodium borohydride, sodium bicarbonate, and reagent-grade tetrahydrofuran (75 to 400 ppm BHT) were used as received for preparation of amine-boranes. For the preparation of alkynylborane-amines, solid alkynes and *n*-butyllithium (1.6 M in hexane) were used as received and liquid alkynes were distilled over lithium aluminum hydride under nitrogen. Anhydrous pentane was prepared by distillation from calcium hydride and stored under nitrogen atmosphere. For the preparation of dibromide substrates, the aldehydes, carbon tetrabromide, triphenylphosphine, and reagent-grade dichloromethane were used as received. Additional solvents used for optimization reactions were distilled from sodium/benzophenone (diethyl ether, tetrahydrofuran, dimethoxyethane), calcium hydride (dichloromethane, toluene), or sodium hydroxide (triethylamine) and stored under nitrogen. Thin-layer chromatography (TLC, Silver Spring, MD, USA) was performed on F60 silica gel plates purchased from Macherey-Nagel (Allentown, PA, USA) and visualized using iodine on silica or UV light. Column chromatography was performed using 60 M Kieselgel silica gel. The identities of the products were confirmed by nuclear magnetic resonance (NMR) spectroscopy and measured in δ values in parts per million (ppm). Spectra of products were recorded from a Bruker (Billerica, MA, USA) 400 MHz, Varian (Palo Alto, CA, USA) INOVA 300 MHz, or Varian (Palo Alto, CA, USA) MERCURY 300 MHz NMR spectrometer. The ^1^H NMR (300 MHz or 400 MHz) spectra were recorded at ambient temperature and calibrated against the residual solvent peak of CDCl_3_ (ẟ = 7.26 ppm) as an internal standard. Coupling constants (J) are given in hertz (Hz), and signal multiplicities are described of NMR data as s = singlet, d = doublet, dd = doublet of doublets, dt = doublet of triplets, ddd = doublet of doublet of doublets, ddt = doublet of doublet of triplets, dqd = doublet of quartet of doublets, dqt = doublet of quartet of triplets, dtd = doublet of triplet of doublets, dddd = doublet of doublet of doublet of doublets, t = triplet, td = triplet of doublets, tt = triplet of triplets, tdd = triplet of doublet of doublets, tdt = triplet of doublet of triplets, q= quartet, p= pentet, h = hextet, m = multiplet, and br = broad. The ^13^C NMR (75 MHz or 101 MHz) spectra were recorded at ambient temperature and calibrated using CDCl_3_ (ẟ = 77.0 ppm) as an internal standard. ^11^B NMR (96 MHz) spectra were recorded at ambient temperature and chemical shifts are reported relative to the external standard, BF_3_:OEt_2_ (ẟ = 0 ppm). ^19^F NMR (282 MHz) spectra were recorded at ambient temperature and chemical shifts are reported relative to the external standard—CFCl_3_ (ẟ = 0 ppm).

### 3.2. Experimental

#### 3.2.1. General Procedure for the Salt Metathesis Synthesis of Amines-Boranes

Following a previously reported procedure [26], sodium borohydride (0.76 g, 20 mmol) and the desired ammonium salt (20 mmol) were weighed to a 100 mL dry round bottom flask, containing a magnetic stir-bar. Then, at rt, reagent-grade tetrahydrofuran (20.0 mL) was added, and the mixture was stirred. Reaction progress was monitored using ^11^B NMR spectroscopy. (Note: A drop of DMSO is added to the reaction aliquot prior to running the ^11^B NMR experiment). Upon completion of the reaction, as determined by ^11^B NMR, the reaction contents were filtered through celite and sodium sulfate and the solid residue washed with additional THF. Removal of the solvent from the filtrate using rotary evaporation yielded the corresponding amine-boranes (**2a**, **2e**). Residual solvent was removed by placing under high vacuum for ~12 h.

#### 3.2.2. General Procedure for the Bicarbonate-Mediated Synthesis of Amines-Boranes

Following a previously reported procedure [21], sodium borohydride (1.51 g, 2 eq., 40 mmol) and powdered sodium bicarbonate (6.72 g, 4 eq., 80 mmol) were weighed to a 100 mL dry round bottom flask, containing a magnetic stir-bar. The desired amine (1 eq., 20 mmol) was added to the flask via syringe, followed by addition of reagent-grade tetrahydrofuran (20 mL) at rt. Under vigorous stirring, water (0.36 mL, 4 eq., 80 mmol) was added dropwise to prevent excessive frothing. Reaction progress was monitored using ^11^B NMR spectroscopy. (Note: A drop of DMSO is added to the reaction aliquot prior to running the ^11^B NMR experiment). Upon completion of the reaction, as determined by ^11^B NMR, the reaction contents were filtered through celite and sodium sulfate and the solid residue washed with additional THF. Removal of the solvent from the filtrate using rotary evaporation yielded the corresponding amine-boranes (**2b**–**2d**, **2f**–**2j**). Residual solvent was removed by placing under high vacuum for ~12 h.

#### 3.2.3. Characterization of Amines-Boranes

*Methylamine-borane (***2a***)*: The compound was prepared as described in the salt metathesis procedure and obtained as a white solid (mass = 0.61 g, 68% yield). ^1^H NMR (300 MHz, CDCl_3_) δ 3.78 (s, 2H), 2.55 (t, *J* = 6.3 Hz, 3H), 1.49 (dd, *J* = 189.1, 92.0 Hz, 3H). ^13^C NMR (75 MHz, CDCl_3_) δ 34.6. ^11^B NMR (96 MHz, CDCl_3_) δ −18.78 (q, *J* = 95.1 Hz). Compound characterization is in agreement with previous reports for this compound [15].
*Propylamine-borane (***2b***)*: The compound was prepared as described in the bicarbonate-mediated procedure and obtained as a white solid (mass = 1.38 g, 95% yield). ^1^H NMR (300 MHz, CDCl_3_) δ 3.81 (s, 2H), 2.75 (p, *J* = 7.2 Hz, 2H), 1.63 (h, *J* = 7.4 Hz, 2H), 0.93 (t, *J* = 7.4 Hz, 3H). ^13^C NMR (75 MHz, CDCl_3_) δ 50.4, 22.4, 11.0. ^11^B NMR (96 MHz, CDCl_3_) δ −19.84 (q, *J* = 92.0 Hz). Compound characterization is in agreement with previous reports for this compound [21].
*Cyclohexylamine-borane (***2c***)*: The compound was prepared as described in the bicarbonate-mediated procedure and obtained as a white solid (mass = 2.19 g, 97% yield). ^1^H NMR (300 MHz, CDCl_3_) δ 3.64 (s, 2H), 2.68 (dqt, *J* = 14.1, 7.6, 4.0 Hz, 1H), 2.13 (d, *J* = 10.4 Hz, 2H), 1.80–1.70 (m, 2H), 1.67–1.56 (m, 1H), 1.37–1.07 (m, 5H). ^13^C NMR (75 MHz, CDCl_3_) δ 57.0, 32.3, 25.3, 24.5. ^11^B NMR (96 MHz, CDCl_3_) δ −0.71–−41.09 (m). Compound characterization is in agreement with previous reports for this compound [21].
*t-Butylamine-borane (***2d***)*: The compound was prepared as described in the bicarbonate-mediated procedure and obtained as a white solid (mass = 1.65 g, 95% yield). ^1^H NMR (300 MHz, CDCl_3_) δ 3.76 (s, 2H), 1.27 (s, 9H). ^13^C NMR (75 MHz, CDCl_3_) δ 53.1, 28.0. ^11^B NMR (96 MHz, CDCl_3_) δ −23.25 (q, *J* = 96.7 Hz). Compound characterization is in agreement with previous reports for this compound [21].
*Dimethylamine-borane (***2e***)*: The compound was prepared as described in the salt metathesis procedure and obtained as a white solid (mass = 1.07 g, 91% yield). ^1^H NMR (300 MHz, CDCl_3_) δ 4.31 (s, 1H), 2.46 (d, *J* = 5.8 Hz, 6H), 1.43 (dd, *J* = 188.2, 91.9 Hz, 3H). ^13^C NMR (75 MHz, CDCl_3_) δ 44.2. ^11^B NMR (96 MHz, CDCl_3_) δ −14.76 (q, *J* = 96.7 Hz). Compound characterization is in agreement with previous reports for this compound [15].
*Diethylamine-borane (***2f***)*: The compound was prepared as described in the bicarbonate-mediated procedure and obtained as a white solid (mass = 1.65 g, 95% yield). ^1^H NMR (300 MHz, CDCl_3_) δ 3.20 (s, 1H), 2.83 (dqt, *J* = 9.7, 7.3, 3.8 Hz, 4H), 1.25 (t, *J* = 7.3 Hz, 6H). ^13^C NMR (75 MHz, CDCl_3_) δ 48.5, 11.3. ^11^B NMR (96 MHz, CDCl_3_) δ −17.07 (q, *J* = 96.7 Hz). Compound characterization is in agreement with previous reports for this compound [21].
*Piperidine-borane (***2g***)*: The compound was prepared as described in the bicarbonate-mediated procedure and obtained as a white solid (mass = 1.96 g, 99% yield). ^1^H NMR (300 MHz, CDCl_3_) δ 3.75 (s, 1H), 3.22 (d, *J* = 13.4 Hz, 2H), 2.59–2.36 (m, 2H), 1.75 (d, *J* = 10.1 Hz, 3H), 1.51 (ddt, *J* = 27.9, 14.2, 3.6 Hz, 2H), 1.32 (tdd, *J* = 16.3, 8.7, 4.6 Hz, 1H). ^13^C NMR (75 MHz, CDCl_3_) δ 53.4, 25.4, 22.6. ^11^B NMR (96 MHz, CDCl_3_) δ −15.55 (q, *J* = 96.7 Hz). Compound characterization is in agreement with previous reports for this compound [21].
*Morpholine-borane (***2h***)*: The compound was prepared as described in the bicarbonate-mediated procedure and obtained as a white solid (mass = 1.98 g, 98% yield). ^1^H NMR (300 MHz, CDCl_3_) δ 4.40 (s, 1H), 3.91 (dd, *J* = 12.6, 3.3 Hz, 2H), 3.55 (td, *J* = 12.3, 2.2 Hz, 2H), 3.05 (d, *J* = 13.7 Hz, 2H), 2.86–2.64 (m, 2H), 2.35–0.65 (m, 3H). ^13^C NMR (75 MHz, CDCl_3_) δ 65.6, 51.8. ^11^B NMR (96 MHz, CDCl_3_) δ −15.47 (q, *J* = 98.0, 96.7 Hz). Compound characterization is in agreement with previous reports for this compound [21].
*Diisopropylamine-borane (***2i***)*: The compound was prepared as described in the bicarbonate-mediated procedure and obtained as a white solid (mass = 2.12 g, 92% yield). ^1^H NMR (300 MHz, CDCl_3_) δ 3.18 (s, 1H), 3.10 (dqd, *J* = 13.1, 6.5, 3.5 Hz, 2H), 1.15 (t, *J* = 6.3 Hz, 12H). ^13^C NMR (75 MHz, CDCl_3_) δ 51.6, 20.6, 18.6. ^11^B NMR (96 MHz, CDCl_3_) δ −21.81 (q, *J* = 96.7 Hz). Compound characterization is in agreement with previous reports for this compound [27].
*Triethylamine-borane (***2j***)*: The compound was prepared as described in the bicarbonate-mediated procedure and obtained as a white solid (mass = 2.16 g, 94% yield). ^1^H NMR (300 MHz, CDCl_3_) δ 2.61 (q, *J* = 7.3 Hz, 6H), 1.02 (t, *J* = 7.3 Hz, 9H). ^13^C NMR (75 MHz, CDCl_3_) δ 51.9, 8.1. ^11^B NMR (96 MHz, CDCl_3_) δ −13.81 (q, *J* = 99.4, 98.0 Hz). Compound characterization is in agreement with previous reports for this compound [21].

#### 3.2.4. General Procedure for the 1 mmol Scale Synthesis of Alkynylborane-Amines

In an oven-dried, 25 mL, round-bottom flask with a side arm and containing a stir bar, the dimethylamine-borane (0.118 g, 0.002 mole, 2 eq.) is weighed along with the alkyne (0.001 mole, 1 eq.) if the alkyne substrate is a solid. A reflux condenser is affix to the flask and the whole apparatus is thoroughly sealed and flushed with nitrogen. Freshly distilled pentane (1 mL) is then charged into the flask through the side arm via syringe. If the alkyne substrate is a liquid, it is added at this time. The reaction mixture is stirred and brought to 0 °C using an ice bath. Then, at 0 °C, *n*-butyllithium (0.001865 mole, 1.865 eq.) is added dropwise as a 1.6 M solution in hexanes through the side arm via syringe. The reaction mixture is stirred for 5 min at 0 °C, and additional pentane (1 mL) is added to help rinse any reaction components from the walls of the flask. The mixture is brought to reflux and monitored by TLC or by ^1^H NMR (with a drop of water added to quench the aliquot). The reaction is typically complete in just 2 h for aromatic alkyne substrates and 6 h for aliphatic alkyne substrates. Reaction completion is judged by the absence of the acetylenic proton in the ^1^H NMR spectrum. Once completed, the reaction mixture is allowed to cool to room temperature, quenched with water (2 mL), and stirred for 30 min. The organic phase is separated, and the aqueous phase is extracted with diethyl ether (3 × 15 mL). All organic layers are combined, dried with sodium sulfate, filtered through cotton, and condensed by rotary evaporation. The crude mixture is then subjected to column chromatography using a 7:2:1, dichloromethane:hexane:diethyl ether solvent system, yielding the corresponding alkynylborane-amines (**3ae**–**3pe**). Residual solvent was removed by placing under high vacuum for ~12 h. As an alternative to column chromatography, the excess dimethylamine-borane can be removed by addition of 1 M HCl (5 mL) following the water quench and stirring for 30 min at rt prior to performing the organic extraction.

#### 3.2.5. Characterization of Alkynylborane-Amines from Terminal Alkynes

*(Phenylethynyl)borane-dimethylamine (***3ae***)*: The compound was prepared as described in the 1 mmol scale procedure and obtained in 2 h as a white solid (mass = 142 mg, 89% yield, 98:2 mono:di ratio); melting point: 74–76 °C (Meltemp). ^1^H NMR (300 MHz, CDCl_3_) δ 7.40 (dt, *J* = 7.5, 1.7 Hz, 2H), 7.35–7.11 (m, 3H), 4.38 (s, 1H), 2.53 (d, *J* = 5.7 Hz, 6H). ^13^C NMR (75 MHz, CDCl_3_) δ 131.2, 127.0, 125.2, 100.0, 42.6. ^11^B NMR (96 MHz, CDCl_3_) δ −15.33 (t, *J* = 100.4 Hz).
*((4-Methoxyphenyl)ethynyl)borane-dimethylamine (***3be***)*: The compound was prepared as described in the 1 mmol scale procedure and obtained in 2 h as a white solid (mass = 183 mg, 97% yield, >99:1 ratio); melting point: 118–120 °C (Meltemp). ^1^H NMR (300 MHz, CDCl_3_) δ 7.35 (d, *J* = 8.7 Hz, 2H), 6.78 (d, *J* = 8.8 Hz, 2H), 4.46 (s, 1H), 3.76 (s, 3H), 2.56 (d, *J* = 5.8 Hz, 6H). ^13^C NMR (75 MHz, CDCl_3_) δ 158.4, 132.6, 117.5, 113.7, 99.7, 55.3, 42.5. ^11^B NMR (96 MHz, CDCl_3_) δ −15.14 (t, *J* = 93.3 Hz).
*((4-Fluorophenyl)ethynyl)borane-dimethylamine (***3ce***)*: The compound was prepared as described in the 1 mmol scale procedure and obtained in 2 h as a white solid (mass = 174 mg, 98% yield, 99:1 ratio); melting point: 102–104 °C (Meltemp). ^1^H NMR (300 MHz, CDCl_3_) δ 7.45–7.33 (m, 2H), 7.00–6.89 (m, 2H), 2.67 (d, *J* = 5.8 Hz, 6H). ^13^C NMR (75 MHz, CDCl_3_) δ 163.1, 159.9, 132.9 (d, *J* = 7.9 Hz), 121.3 (d, *J* = 3.0 Hz), 115.9–114.6 (m), 98.9, 42.6. ^11^B NMR (96 MHz, CDCl_3_) δ −15.17 (t, *J* = 99.9 Hz). ^19^F NMR (282 MHz, CDCl_3_) δ −114.72–114.96 (m).
*((4-Ttrifluoromethyl)phenyl)ethynyl)borane-dimethylamine (***3de***)*: The compound was prepared as described in the 1 mmol scale procedure and obtained in 2 h as a white solid (mass = 198 mg, 87% yield, 98:2 ratio); white solid, melting point: 80–82 °C (Meltemp). ^1^H NMR (300 MHz, CDCl_3_) δ 7.49 (s, 4H), 2.69 (d, *J* = 5.8 Hz, 6H). ^13^C NMR (75 MHz, CDCl_3_) δ 131.3, 129.1, 128.7, 128.2, 125.8, 125.0, 124.9, 122.2, 98.9, 42.6. ^11^B NMR (96 MHz, CDCl_3_) δ −15.23 (t, *J* = 100.6 Hz). ^19^F NMR (282 MHz, CDCl_3_) δ −64.14 (s).
*((2-Methylphenyl)ethynyl)borane-dimethylamine (***3ee***)*: The compound was prepared as described in the 1 mmol scale procedure and obtained in 2 h as a clear, colorless liquid (mass = 168 mg, 97% yield, 97:3 ratio); ^1^H NMR (300 MHz, CDCl_3_) δ 7.39 (d, *J* = 7.4 Hz, 1H), 7.13 (dddd, *J* = 14.8, 9.1, 7.2, 2.3 Hz, 3H), 4.28 (s, 1H), 2.58 (d, *J* = 5.8 Hz, 6H), 2.45 (s, 3H). ^13^C NMR (75 MHz, CDCl_3_) δ 139.3, 131.5, 129.2, 126.9, 125.3, 125.0, 42.5, 21.2. ^11^B NMR (96 MHz, CDCl_3_) δ −15.17 (t, *J* = 99.6 Hz).
*((3-Methylphenyl)ethynyl)borane-dimethylamine (***3fe***)*: The compound was prepared as described in the 1 mmol scale procedure and obtained in 2 h as a clear, colorless liquid (mass = 146 mg, 84% yield, 96:4 ratio); ^1^H NMR (300 MHz, CDCl_3_) δ 7.29–7.18 (m, 2H), 7.13 (t, *J* = 7.5 Hz, 1H), 7.02 (d, *J* = 7.6 Hz, 1H), 4.39 (s, 1H), 2.55 (d, *J* = 5.8 Hz, 6H), 2.28 (s, 3H). ^13^C NMR (75 MHz, CDCl_3_) δ 131.9, 128.3, 128.0, 127.9, 125.0, 100.2, 42.6, 21.4. ^11^B NMR (96 MHz, CDCl_3_) δ −15.40 (t, *J* = 100.0 Hz).
*((4-Methylphenyl)ethyny)borane-dimethylamine (***3ge***)*: The compound was prepared as described in the 1 mmol scale procedure and obtained in 2 h as a white solid (mass = 149 mg, 86% yield, 99:1 ratio); melting point: 75–77 °C (Meltemp). ^1^H NMR (300 MHz, CDCl_3_) δ 7.30 (d, *J* = 7.9 Hz, 2H), 7.04 (d, *J* = 7.8 Hz, 2H), 4.44 (s, 1H), 2.52 (d, *J* = 5.8 Hz, 6H), 2.29 (s, 3H). ^13^C NMR (75 MHz, CDCl_3_) δ 136.8, 131.1, 128.9, 122.2, 100.1, 42.5, 21.5. ^11^B NMR (96 MHz, CDCl_3_) δ −15.10 (t, *J* = 99.7 Hz).
*(4-Phenylbut-1-yn-1-yl)borane-dimethylamine (***3he***)*: The compound was prepared as described in the 1 mmol scale procedure and obtained in 6 h as a white solid (mass = 140 mg, 75% yield, 88:12 ratio); melting point: 69–72 °C (Meltemp). ^1^H NMR (300 MHz, CDCl_3_) δ 7.36–7.08 (m, 5H), 4.14 (s, 1H), 2.83 (t, *J* = 7.6 Hz, 2H), 2.52 (t, *J* = 7.8 Hz, 2H), 2.44 (d, *J* = 5.7 Hz, 6H). ^13^C NMR (75 MHz, CDCl_3_) δ 141.3, 128.4, 128.2, 42.2, 36.1, 22.3. ^11^B NMR (96 MHz, CDCl_3_) δ −15.35 (t, *J* = 99.7 Hz).
*(Hexynyl)borane-dimethylamine (***3ie***)*: The compound was prepared as described in the 1 mmol scale procedure and obtained in 6 h as a clear, colorless liquid (mass = 114 mg, 82% yield, 94:6 ratio); ^1^H NMR (300 MHz, CDCl_3_) δ 2.61 (d, *J* = 5.8 Hz, 6H), 2.37–2.06 (m, 2H), 1.63–1.28 (m, 4H), 1.02–0.72 (m, 3H). ^13^C NMR (75 MHz, CDCl_3_) δ 100.7, 42.2, 31.9, 22.2, 19.8, 13.8. ^11^B NMR (96 MHz, CDCl_3_) δ −15.51 (t, *J* = 98.8 Hz).
*(Decynyl)borane-dimethylamine (***3je***)*: The compound was prepared as described in the 1 mmol scale procedure and obtained in 5 h as a clear, colorless liquid (mass = 170 mg, 87% yield, 87:13 ratio); ^1^H NMR (300 MHz, CDCl_3_) δ 4.40 (s, 1H), 2.59 (d, *J* = 5.8 Hz, 6H), 2.21 (t, *J* = 7.2 Hz, 2H), 1.51 (p, *J* = 6.9 Hz, 2H), 1.44–1.34 (m, 2H), 1.34–1.17 (m, 8H), 0.87 (t, *J* = 6.4 Hz, 3H). ^13^C NMR (75 MHz, CDCl_3_) δ 100.7, 42.2, 31.9, 29.8, 29.3, 29.3, 29.2, 22.7, 20.1, 14.2. ^11^B NMR (96 MHz, CDCl_3_) δ −15.50 (t, *J* = 100.8 Hz).
*(3,3-Dimethylbut-1-yn-1-yl)borane-dimethylamine (***3ke***)*: The compound was prepared as described in the 1 mmol scale procedure and obtained in 4 h as a clear, white solid (mass = 1370 mg, 93% yield, 99:1 ratio);White solid, Melting point: 118–120 °C (Meltemp). ^1^H NMR (300 MHz, CDCl_3_) δ 4.03 (s, 1H), 2.61 (d, *J* = 5.9 Hz, 6H), 1.23 (s, 9H). ^13^C NMR (75 MHz, CDCl_3_) δ 109.7, 42.2, 31.9, 28.1. ^11^B NMR (96 MHz, CDCl_3_) δ −15.13 (t, *J* = 98.4 Hz).
*((Cyclopentyl)ethynyl)borane-dimethylamine (***3le***)*: The compound was prepared as described in the 1 mmol scale procedure and obtained in 4 h as a clear, white solid (mass = 135 mg, 89% yield, 99:1 ratio); melting point: 67–70 °C (Meltemp). ^1^H NMR (300 MHz, CDCl_3_) δ 4.36 (s, 1H), 2.60 (d, *J* = 5.7 Hz, 6H), 1.99–1.81 (m, 2H), 1.81–1.64 (m, 2H), 1.64–1.43 (m, 4H). ^13^C NMR (75 MHz, CDCl_3_) δ 42.2, 34.6, 31.5, 25.0. ^11^B NMR (96 MHz, CDCl_3_) δ −15.47 (t, *J* = 98.6 Hz).
*((Trimethylsilyl)ethynyl)borane-dimethylamine (***3me***)*: The compound was prepared as described in the 1 mmol scale procedure and obtained in 6 h as a clear, white solid (mass = 129 mg, 83% yield, 98:2 ratio); melting point: 78–80 °C (Meltemp) ^1^H NMR (300 MHz, CDCl_3_) δ 2.64 (d, *J* = 5.8 Hz, 6H), 0.14 (s, 9H). ^13^C NMR (75 MHz, CDCl_3_) δ 42.7, 1.1. ^11^B NMR (96 MHz, CDCl_3_) δ −15.59 (t, *J* = 99.9 Hz).
*(3,3,3-Trifluoroprop-1-yn-1-yl)borane-dimethylamine (***3ne***)*: The compound was prepared as described in the 1 mmol scale procedure and obtained in 12 h as a clear, pale yellow liquid (mass = 98 mg, 65% yield, 98:2 ratio); ^1^H NMR (300 MHz, CDCl_3_) δ 3.82 (s, 1H), 2.63 (d, *J* = 5.8 Hz, 6H), 1.90 (dd, *J* = 191.7, 97.4 Hz, 2H). ^13^C NMR (75 MHz, CDCl_3_) δ 113.9 (q, *J* = 254.5 Hz), 42.7. ^11^B NMR (96 MHz, CDCl_3_) δ −16.30 (t, *J* = 102.1 Hz). ^19^F NMR (282 MHz, CDCl_3_) δ −50.14.
*((Cyclohex-1-en-1-yl)ethynyl)borane-dimethylamine (***3oe***)*: The compound was prepared as described in the 1 mmol scale procedure and obtained in 5 h as a white solid (mass = 129 mg, 79% yield, 81:19 ratio); melting point: 80–82 °C (Meltemp). ^1^H NMR (300 MHz, CDCl_3_) δ 5.98 (tt, *J* = 3.7, 1.6 Hz, 1H), 4.34 (s, 1H), 2.60 (d, *J* = 5.9 Hz, 6H), 2.13 (tt, *J* = 5.7, 2.3 Hz, 2H), 2.05 (h, *J* = 3.1 Hz, 2H), 1.58 (tdt, *J* = 10.4, 5.6, 2.7 Hz, 4H). ^13^C NMR (75 MHz, CDCl_3_) δ 131.8, 122.0, 102.1, 42.4, 30.1, 25.6, 22.6, 21.8. ^11^B NMR (96 MHz, CDCl_3_) δ −15.20 (t, *J* = 99.7 Hz).
*(Deca-1,5-diyn-1-yl)borane-dimethylamine (***3pe***)*: The compound was prepared as described in the 1 mmol scale procedure and obtained in 5 h as a clear, colorless liquid (mass = 157 mg, 82% yield, 85:15 ratio); ^1^H NMR (300 MHz, CDCl_3_) δ 4.11 (s, 1H), 2.61 (d, *J* = 5.8 Hz, 6H), 2.50–2.30 (m, 4H), 2.14 (tt, *J* = 6.9, 2.0 Hz, 2H), 1.56–1.31 (m, 4H), 0.90 (t, *J* = 7.1 Hz, 2H). ^13^C NMR (75 MHz, CDCl_3_) δ 99.0, 80.8, 79.0, 42.2, 31.2, 22.0, 20.7, 20.0, 18.5, 13.7. ^11^B NMR (96 MHz, CDCl_3_) δ −15.39 (t, *J* = 100.2 Hz).

#### 3.2.6. General Procedure for the Synthesis of Dibromide Substrates

Into a 250 mL round bottom flask containing a stir bar was weighed CBr_4_ (6.63 g, 20 mmol). The flask was purged using N_2_, then CH_2_Cl_2_ (30 mL) was added, and the mixture was stirred to dissolve the solid. The mixture was brough to 0 °C using an ice bath then PPh_3_ (10.5 g, 40 mmol) was added, and the mixture was stirred 1 h at 0 °C. The aldehyde (10 mmol) was then added, and the mixture was stirred for an additional 1 h at 0 °C. Then, at 0 °C, the reaction was quenched with H_2_O (10 mL). The organics were separated, then washed with brine (10 mL), dried using MgSO_4_ and concentrated in a 1 L flask using rotary evaporation. Hexane (250 mL) was added to the residue and the supernatant layer was collected. The remaining residue was redissolved in CH_2_Cl_2_ (50 mL) and concentrated. Hexane (250 mL) was added to the residue and the supernatant layer was collected. This process was repeated once more. The combined supernatant layers were passed through silica gel (100 g) and the eluent was concentrated to yield the dibromide product.

#### 3.2.7. Characterization of Dibromide Substrates

*1-(2,2-Dibromovinyl)-4-methoxybenzene (***6a***)*: The compound was prepared as described in the dibromide synthesis procedure and obtained as a white solid (mass = 1.87 g, 64% yield). ^1^H NMR (300 MHz, CDCl_3_) δ 7.56–7.47 (m, 2H), 7.41 (s, 1H), 6.94–6.85 (m, 2H), 3.82 (s, 3H). ^13^C NMR (75 MHz, CDCl_3_) δ 159.5, 136.1, 129.8, 127.7, 113.7, 87.2, 55.3. Compound characterization is in agreement with previous reports for this compound [28].
*(4-(2,2-Dibromovinyl)phenyl)(methyl)sulfane (***6b***)*: The compound was prepared as described in the dibromide synthesis procedure and obtained as a pale-yellow solid (mass = 1.42 g, 46% yield). ^1^H NMR (300 MHz, CDCl_3_) δ 7.50–7.45 (m, 2H), 7.42 (s, 1H), 7.25–7.18 (m, 2H), 2.49 (s, 3H). ^13^C NMR (75 MHz, CDCl_3_) δ 139.5, 136.1, 131.6, 128.7, 125.7, 88.9, 15.4. Compound characterization is in agreement with previous reports for this compound [29].
*2-(2,2-Dibromovinyl)thiophene (***6c***)*: The compound was prepared as described in the dibromide synthesis procedure and obtained as a white solid (mass = 2.04 g, 76% yield). ^1^H NMR (300 MHz, CDCl_3_) δ 7.66 (d, *J* = 0.7 Hz, 1H), 7.39 (ddt, *J* = 5.1, 1.3, 0.6 Hz, 1H), 7.25 (ddt, *J* = 3.6, 1.2, 0.6 Hz, 1H), 7.04 (ddd, *J* = 5.2, 3.7, 0.5 Hz, 1H). ^13^C NMR (75 MHz, CDCl_3_) δ 137.9, 130.7, 129.9, 127.0, 126.4, 86.9. Compound characterization is in agreement with previous reports for this compound [28].
*1,1-Dibromo-4-methylpent-1-ene (***6d***)*: The compound was prepared as described in the dibromide synthesis procedure and obtained as a pale-yellow liquid (mass = 2.01 g, 83% yield). ^1^H NMR (400 MHz, CDCl_3_) δ 6.40 (td, *J* = 7.3, 2.0 Hz, 1H), 1.99 (td, *J* = 7.1, 2.0 Hz, 2H), 1.75 (dtd, *J* = 13.4, 6.7, 2.0 Hz, 1H), 0.93 (dd, *J* = 6.7, 2.1 Hz, 6H). ^13^C NMR (101 MHz, CDCl_3_) δ 137.7, 88.8, 41.7, 27.7, 22.1. Compound characterization is in agreement with previous reports for this compound [30].

#### 3.2.8. General Procedure for the Synthesis of Alkynylborane-Amines from Dibromide Substrates

In an oven-dried, 25 mL, round-bottom flask with a side arm and containing a stir bar, the dibromide (0.001 mole, 1 eq.) is weighed. A reflux condenser is affixed to the flask and the whole apparatus is thoroughly sealed and flushed with nitrogen. The flask was sealed and purged with nitrogen. Freshly distilled pentane (4 mL) is then charged into the flask through the side arm via syringe. The mixture is brought to −78 °C using a dry ice/acetone bath, and stirred to cool. Then, *n*-BuLi (2.3 eq., 2.3 mmol, 1.5 mL) is added dropwise at −78 °C. The reaction is stirred for 30 min at −78 °C then then brought to 0 °C using an ice bath and stirred for an additional 30 min @ 0 °C. Dimethylamine-borane (0.118 g, 0.002 mole, 2 eq.) is added to the flask @ 0 °C and stirred for an additional 30 min at rt. Then, 2 mL of pentane is added to wash the walls of the flask and the solution brought to reflux and monitored by TLC or by ^1^H NMR (with a drop of water added to quench the aliquot). Reaction completion is judged by the absence of the acetylenic proton in the by ^1^H NMR spectrum. Once completed, the reaction mixture is allowed to cool to room temperature, quenched with water (2 mL), and stirred for 30 min at rt, followed by 1 M HCl (5 mL) and stirred for an additional 30 min at rt. The organic layer is separated and the water extracted with ethyl acetate 3 times. The combined extracts are dried with sodium sulfate and filtered through cotton, then condensed using rotary evaporation, yielding the corresponding alkynylborane-amines (**7ae**–**7de**). Residual solvent is removed by placing under high vacuum for ~12 h.

#### 3.2.9. Characterization of Alkynylborane-Amines from Dibromide Substrates

*((4-Methoxyphenyl)ethynyl)borane-dimethylamine (***7ae***)*: The compound was prepared as described in the alkynylborane-amine from dibromide procedure and obtained in 2 h as a white solid (mass = 168 mg, 89% yield, >99:1 ratio); melting point: 118–120 ^o^C (Meltemp). ^1^H NMR (300 MHz, CDCl_3_) δ 7.39–7.32 (m, 2H), 6.82–6.75 (m, 2H), 3.94 (s, 2H), 3.79 (s, 3H), 2.66 (d, *J* = 5.8 Hz, 6H). ^13^C NMR (75 MHz, CDCl_3_) δ 158.20, 132.33, 117.24, 113.46, 99.50, 55.07, 42.30. ^11^B NMR (96 MHz, CDCl_3_) δ −15.14 (t, *J* = 89.1 Hz).
*((4-(Methylthio)phenyl)ethynyl)borane-dimethylamine (***7be***)*: The compound was prepared as described in the alkynylborane-amine from dibromide procedure and obtained in 2 hours as a white solid (mass = 178 mg, 87% yield, >99:1 ratio); melting point: 118–121 °C (Meltemp). ^1^H NMR (300 MHz, CDCl_3_) δ 7.40–7.28 (m, 2H), 7.18–7.05 (m, 2H), 3.79 (s, 1H), 2.68 (d, *J* = 5.8 Hz, 6H), 2.47 (s, 3H). ^13^C NMR (75 MHz, CDCl_3_) δ 137.2, 131.6, 125.9, 121.9, 42.6, 15.7. ^11^B NMR (96 MHz, CDCl_3_) δ −15.10 (t, *J* = 98.0 Hz).
*(Thiophen-2-ylethynyl)borane-dimethylamine (***7ce***)*: The compound was prepared as described in the alkynylborane-amine from dibromide procedure and obtained in 7 hours as a pale-yellow solid (mass = 155 mg, 94% yield, 94:6 ratio); melting point: 139–142 °C (Meltemp). ^1^H NMR (300 MHz, CDCl_3_) δ 7.14–7.07 (m, 2H), 6.91 (dd, *J* = 5.2, 3.6 Hz, 1H), 3.79 (s, 1H), 2.67 (d, *J* = 5.8 Hz, 6H). ^13^C NMR (101 MHz, CDCl_3_) δ 130.2, 126.6, 125.7, 125.1, 42.5. ^11^B NMR (96 MHz, CDCl_3_) δ −15.13 (t, *J* = 102.1 Hz).
*(4-Methylpent-1-yn-1-yl)borane-dimethylamine (***7de***)*: The compound was prepared as described in the alkynylborane-amine from dibromide procedure and obtained in 4 hours as a clear, colorless liquid (mass = 123 mg, 89% yield, 93:7 ratio); ^1^H NMR (300 MHz, CDCl_3_) δ 3.82 (s, 1H), 2.62 (d, *J* = 5.8 Hz, 6H), 2.11 (dt, *J* = 6.7, 1.9 Hz, 2H), 1.85–1.70 (m, 1H), 0.97 (d, *J* = 6.6 Hz, 6H). ^13^C NMR (101 MHz, CDCl_3_) δ 42.2, 29.3, 28.6, 22.1. ^11^B NMR (96 MHz, CDCl_3_) δ −15.26 (t, *J* = 99.4 Hz).

## 4. Conclusions

Dehydrogenative borylation utilizing air- and moisture-stable amine-boranes has been presented. Terminal alkynes, as well as 1,1-dibromoolefins derived from aldehydes, act as suitable substrates for the reaction with lithium aminoborohydrides generated in situ from the amine-borane and *n*-butyllithium or treating an amine-borane with an alkynyllithium. A wide variety of substrates are amenable to this methodology, including aromatic, heteroaromatic, and aliphatic substrates, with the primary factor limiting the substrate scope being the reducing capabilities of the active lithium aminoborohydride reagent. The *B*-alkynylated amine-borane products are obtained in high yield and are stable to column chromatography as well as acidic and basic aqueous conditions. Though the mono-*B*-alkynylated product is primarily generated using the presented conditions, the potential to form mono-, di-, and tri-B-alkynylated products has been demonstrated. The reaction has additionally been demonstrated at large (up to 50 mmol) scale. Work is currently underway investigating the applications and transformation of these alkynylated amine-borane compounds.

## Figures and Tables

**Table 1:** Optimization of the reaction solvent and amine-borane ^a^.

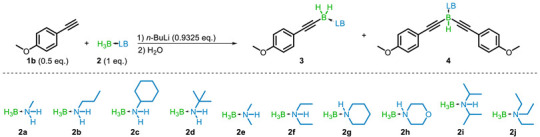						
Entry	Condition	Solvent	Amine-Borane	Products	Ratio ^b^	Conversion ^c^
1	rt, 24 h	Et_2_O	**2e**	**3ae/4ae**	85:15	95%
2	rt, 24 h	THF	**2e**	**3ae/4ae**	73:27	51%
3	rt, 24 h	DME	**2e**	**3ae/4ae**	80:20	30%
4	rt, 24 h	DCM	**2e**	**3ae/4ae**	87:13	80%
5	rt, 24 h	toluene	**2e**	**3ae/4ae**	100:0	79%
6	rt, 24 h	pentane	**2e**	**3ae/4ae**	99:1	92%
7	rt, 24 h	Et_3_N	**2e**	**3ae/4ae**	85:15	80%
8	reflux, 2 h	pentane	**2e**	**3ae/4ae**	99:1	97%
9	reflux, 2 h	pentane	**2a**	**3aa/4aa**	-	trace
10	reflux, 2 h	pentane	**2b**	**3ab/4ab**	-	trace
11	reflux, 2 h	pentane	**2c**	**3ac/4ac**	99:1	16%
12	reflux, 2 h	pentane	**2d**	**3ad/4ad**	92:8	82%
13	reflux, 2 h	pentane	**2f**	**3af/4af**	99:1	66%
14	reflux, 2 h	pentane	**2g**	**3ag/4ag**	99:1	83%
15	reflux, 2 h	pentane	**2h**	**3ah/4ah**	-	trace
16	reflux, 2 h	pentane	**2i**	**3ai/4ai**	93:7	14%
17	reflux, 2 h	pentane	**2j**	**3aj/4aj**	-	0%

^a^ Reactions were carried out using optimized stoichiometry with 4-methoxyphenylacetylene. ^b^ Ratio of mono- to di-B-substituted product. ^c^ Alkyne conversion includes both mono- and di-B-substituted products.

## Data Availability

All of the ^1^H, ^11^B, ^13^C, and ^19^F NMR spectra are available in the Supporting Information.

## Supplementary Materials

The following Supporting Information can be downloaded at: https://www.mdpi.com/article/10.3390/molecules28083433/s1, Characterization and NMR spectra of initial mono-, di-, and tri-B-alkynylated borane-amines, reaction optimization data, and NMR spectra of amine-boranes, alkynylborane-amine from terminal alkynes, dibromide substrates, and alkynylborane-amine from dibromides.

## Sample Availability

Samples of the compounds are not available from the authors.
