# Supplementary material for: Dehydroborylation of Terminal Alkynes Using Lithium Aminoborohydrides
Source: Molecules. 2023 Apr 13;28(8):3433. doi: 10.3390/molecules28083433 (PMC10144115; doi:10.3390/molecules28083433)
Supplement: Supplementary file 1 [file molecules-28-03433-s001.zip › molecules-2329941-supplementary.pdf]

SUPPORTING INFORMATION:

Dehydroborylation of Terminal Alkynes Using Lithium Aminoborohydrides

P. Veeraraghavan Ramachandran* and Henry J. Hamann

Herbert C. Brown Center for Borane Research, Department of Chemistry, Purdue University, West Lafayette, Indiana 47907, United States

E-mail: chandran@purdue.edu

Contents:

Page

Initial mono-, di- and trialkynylborane-amines S2-S5
Reaction optimization data S5-S6
NMR spectra of amine-boranes S7-S21
NMR spectra of alkynylborane-amines from terminal alkynes S22-S47
NMR spectra of dibromides S47-S51
NMR spectra of alkynylborane-amines from dibromides S51-S57

Initial mono-, di- and trialkynylborane-amines:

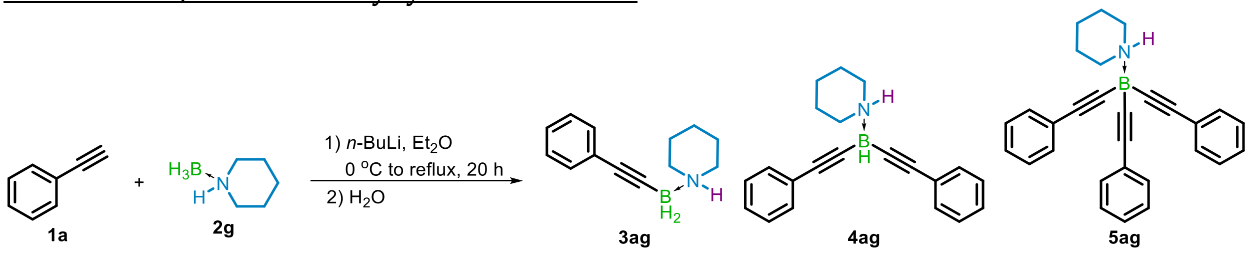

(**3ag** : **4ag** : **5ag**) = (8 : 82 : 10)

Scheme S1. Initial reaction of terminal alkyne and amine-borane.

Characterization

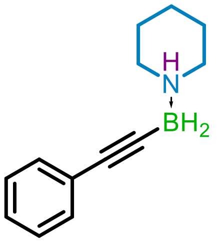

(phenylethynyl)borane-piperidine (**3ag**); Melting point: 145-147 °C (DSC). **^{1}H NMR** (300 MHz, Chloroform-*d*) δ 7.49 – 7.37 (m, 2H), 7.31 – 7.16 (m, 3H), 3.49 (d, *J* = 13.4 Hz, 2H), 3.35 (s, 1H), 2.76 – 2.56 (m, 2H), 1.84 (d, *J* = 14.1 Hz, 3H), 1.66 – 1.30 (m, 4H). **^{13}C NMR** (75 MHz, Chloroform-*d*) δ 131.1, 127.9, 126.7, 125.4, 51.8, 25.3, 23.0. **^{11}B NMR** (96 MHz, Chloroform-*d*) δ -16.09 (t, *J* = 102.1 Hz). **HRMS** (ESI) calcd for $C_{13}H_{18}BNNa$ $(M+Na)^+$: *m/z*, 221.1466, found 221.1462.

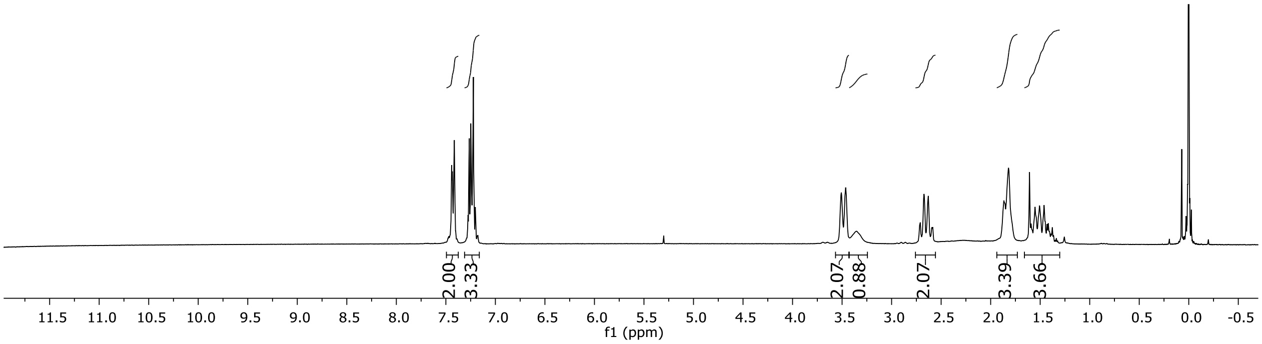

^{1}H NMR (300 MHz, $CDCl_3$) (phenylethynyl)borane-piperidine (**3ag**)

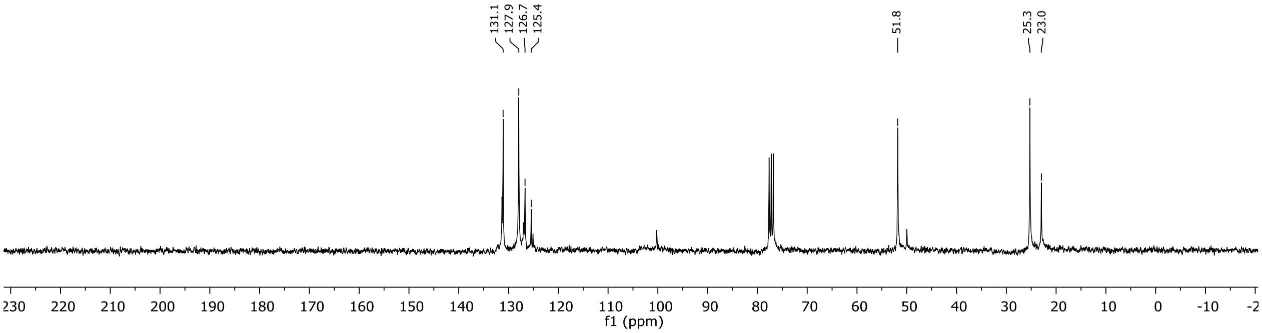

^{13}C NMR (75 MHz, $CDCl_3$) (phenylethynyl)borane-piperidine (**3ag**)

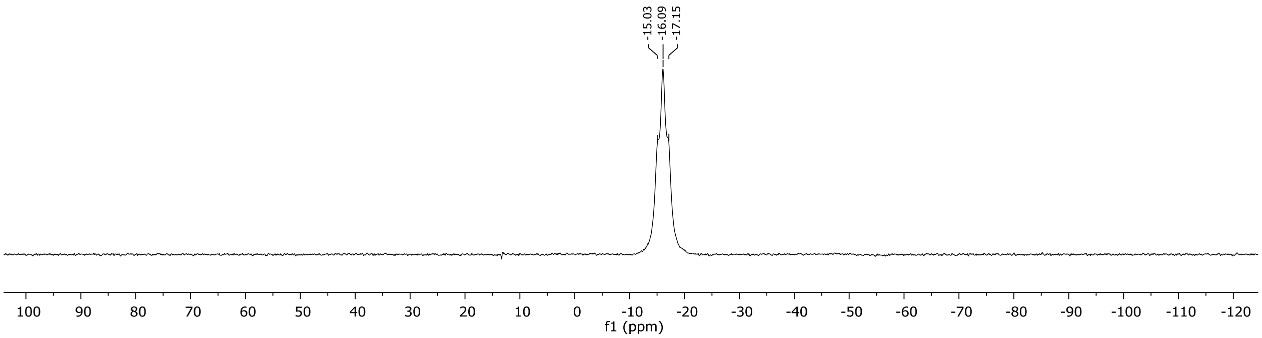

^{11}B NMR (96 MHz, $CDCl_3$) (phenylethynyl)borane-piperidine (**3ag**)

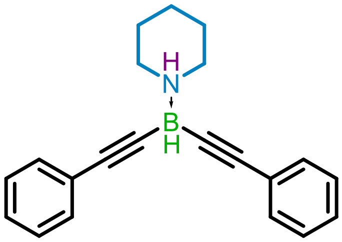

bis(phenylethynyl)borane-piperidine (**4ag**); Melting point: 105-107 °C (DSC). **^{1}H NMR** (400 MHz, Chloroform-*d*) δ 7.49 – 7.43 (m, 4H), 7.29 – 7.21 (m, 6H), 3.67 (d, *J* = 13.5 Hz, 2H), 3.23 (s, 1H), 2.89 (q, *J* = 12.4 Hz, 2H), 1.89 (t, *J* = 18.0 Hz, 3H), 1.65 – 1.49 (m, 3H). **^{13}C NMR** (101 MHz, Chloroform-*d*) δ 131.5, 127.9, 127.0, 125.2, 108.6, 49.7, 25.4, 22.8. **^{11}B NMR** (96 MHz, Chloroform-*d*) δ -16.42 (d, *J* = 102.1 Hz). **HRMS** (ESI) calcd for $C_{21}H_{22}BNNa$ (M+Na)$^+$: *m/z*, 321.1779, found 321.1775.

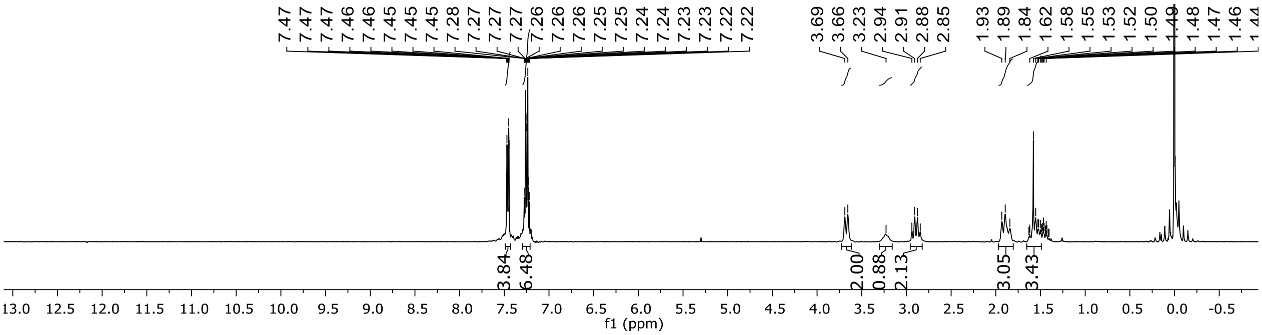

^{1}H NMR (300 MHz, $CDCl_3$) bis(phenylethynyl)borane-piperidine (**4ag**)

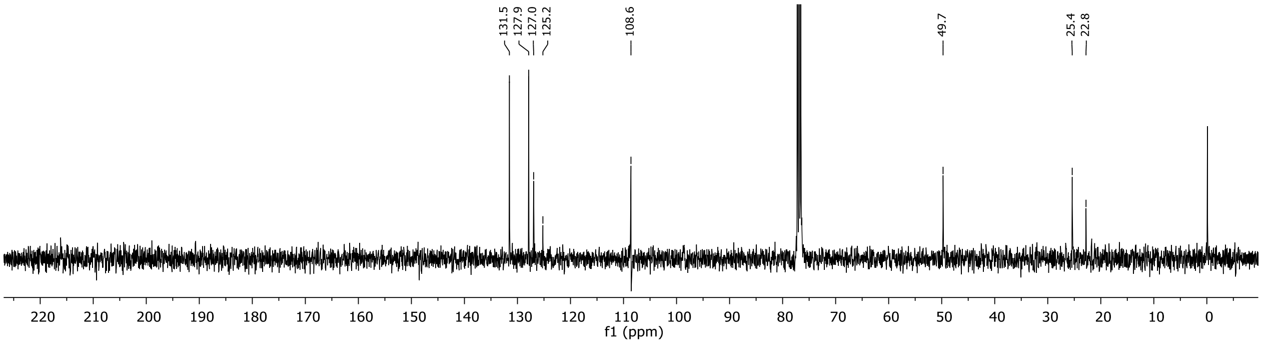

^{13}C NMR (75 MHz, $CDCl_3$) bis(phenylethynyl)borane-piperidine (**4ag**)

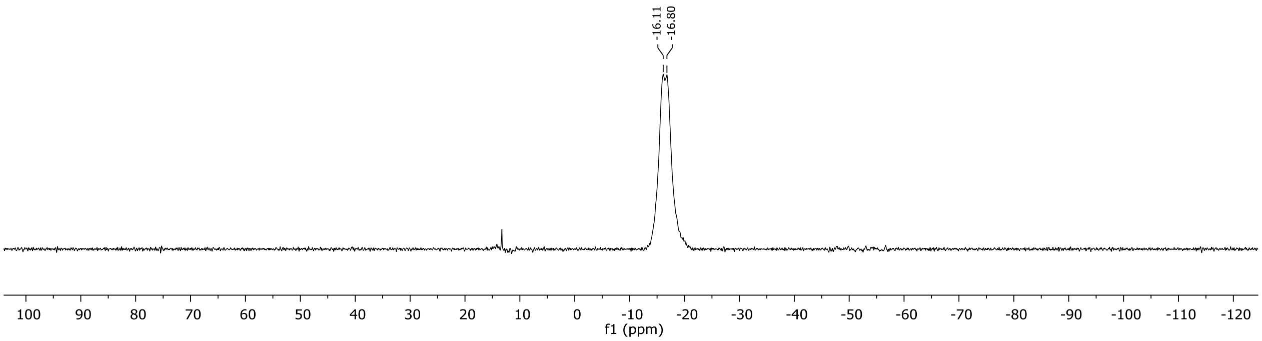

^{11}B NMR (96 MHz, $CDCl_3$) bis(phenylethynyl)borane-piperidine (**4ag**)

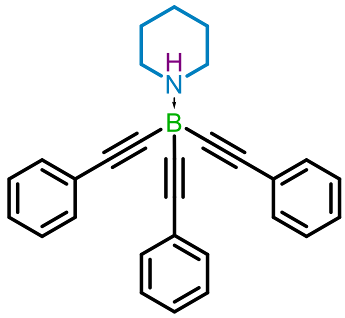

tris(phenylethynyl)borane-piperidine (**5ag**); Melting point: 232-235 °C (DSC). **^{1}H NMR** (400 MHz, Chloroform-*d*) δ 7.57 – 7.41 (m, 6H), 7.33 – 7.14 (m, 9H), 3.96 (d, *J* = 13.3 Hz, 2H), 3.23 (d, *J* = 11.4 Hz, 1H), 3.09 – 2.85 (m, 2H), 1.93 (dd, *J* = 29.9, 13.5 Hz, 3H), 1.67 – 1.43 (m, 3H). **^{13}C NMR** (101 MHz, Chloroform-*d*) δ 131.66, 127.86, 127.16, 124.97, 48.20, 25.47, 22.97. **^{11}B NMR** (96 MHz, Chloroform-*d*) δ -16.59. **HRMS** (ESI) calcd for $C_{29}H_{26}BNNa$ $(M+Na)^+$: *m/z*, 421.2092, found 421.2086.

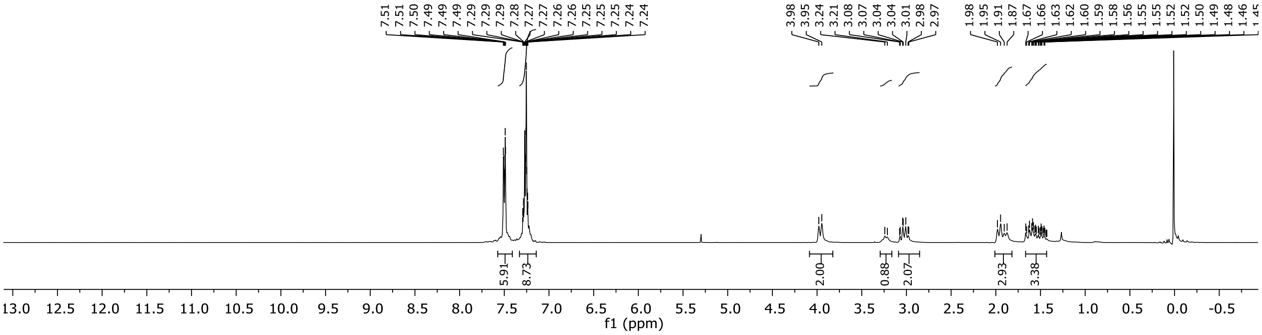

^{1}H NMR (300 MHz, $CDCl_3$) tris(phenylethynyl)borane-piperidine (**5ag**)

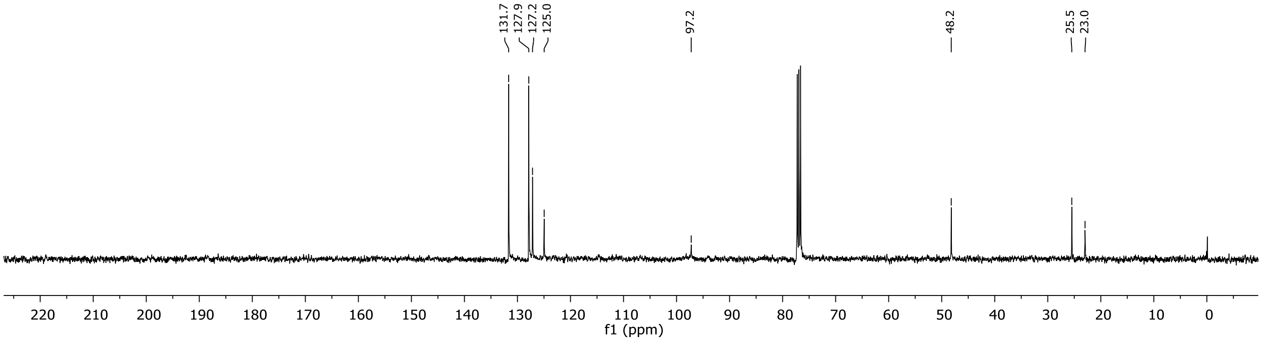

^{13}C NMR (75 MHz, $CDCl_3$) tris(phenylethynyl)borane-piperidine (**5ag**)

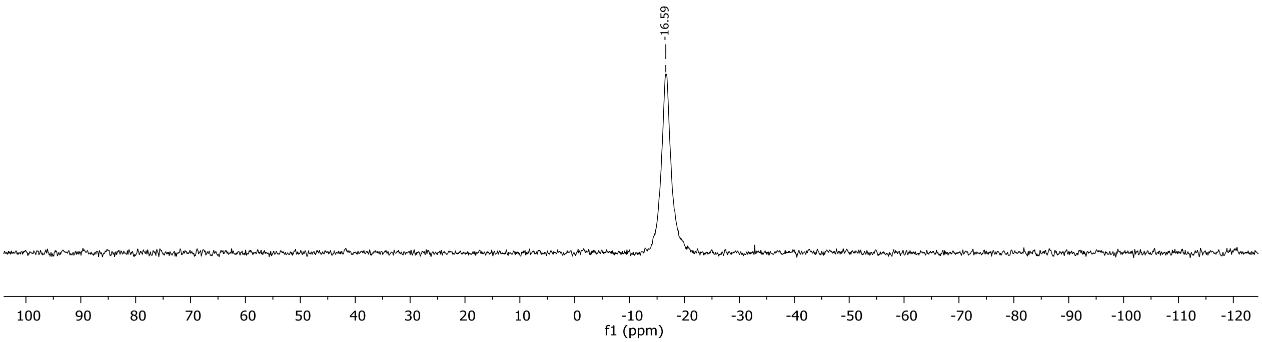

^{11}B NMR (96 MHz, $CDCl_3$) tris(phenylethynyl)borane-piperidine (**5ag**)

Reaction optimization:

Table S1. Examination of order of reagent addition

Addition Type[a]	Equivalency DMAB:nBuLi:Alkyne	Alkyne Conversion[b,c]
LAB formation	1:1:0.3	69%
Acetylide formation	1:1:0.3	70%
Parallel addition	1:1:0.3	70%
LAB formation	1:1:0.5	51%
Acetylide formation	1:1:0.5	48%
Parallel addition	1:1:0.5	50%

[a]The reactions were carried out using diethyl ether as the solvent and 4-methoxyphenylacetylene as the alkyne. [b]Isolated yield, but without complete chromatographic separation. [c]Alkyne conversion includes both mono- and di- substituted products

Table S2. Examination of reagent equivalency

Conditions[a]	Equivalency DMAB:nBuLi:Alkyne	Product Ratio[b]	Alkyne Conversion[c,d]
rt, 48 h	1:1:1	78:22	68%
rt, 24 h	1:1:0.8	94:6	75%
rt, 24 h	1: 0.9325:0.8	91:9	56%
rt, 24 h	1:1:0.65	92:8	66%
rt, 24 h	1: 0.875:0.65	87:3	82%
rt, 24 h	1:1:0.5	87:13	98%
rt, 24 h	1:0.9325:0.5	85:15	99%
rt, 24 h	1:0.75:0.5	90:10	74%
rt, 24 h	1:1:0.3	99:1	66%

[a]The reactions were carried out using diethyl ether as the solvent and 4-methoxyphenylacetylene as the alkyne. [b]Ratio is of mono- substituted to di- substituted product. [c]Isolated yield, but without complete chromatographic separation. [d]Alkyne conversion includes both mono- and di- substituted products

Table S3. Examination of reaction duration

Conditions[a]	Product Ratio[b]	Alkyne Conversion[c,d]
6 h	97:3	70%
12 h	94:6	78%
24 h	85:15	95%

[a]The reactions were carried out at room temperature, using diethyl ether as the solvent, dimethylamine-borane as the amine-borane, and 4-methoxyphenylacetylene as the alkyne. [b]Ratio is of mono- substituted to di- substituted product. [c]Isolated yield, but without complete chromatographic separation. [d]Alkyne conversion includes both mono- and di- substituted products

Table S4. Examination of reaction solvent[a]

Solvent	Product Ratio[b]	Alkyne Conversion[c,d]
Diethyl ether	85:15	95%
Tetrahydrofuran	73:27	51%
Dimethoxymethane	80:20	30%
Dichloromethane	87:13	80%
**Toluene**	**100:0**	**79%**
**Pentane**	**99:1**	**92%**
Triethylamine	85:15	80%

[a]The reactions were carried out at room temperature, using dimethylamine-borane as the amine-borane, and 4-methoxyphenylacetylene as the alkyne. [b]Ratio is of mono- substituted to di- substituted product. [c]Isolated yield, but without complete chromatographic separation. [d]Alkyne conversion includes both mono- and di- substituted products

Table S5. Final reaction conditions standardization[a]

Reaction Conditions	Equivalency DMAB:nBuLi:Alkyne	Product Ratio[b]	Alkyne Conversion[c,d]
rt, 24 h, pentane (0.33 M)	2:1.865:1	99:1	59%
rt, 36 h, pentane (1 M)	2:1.865:1	99:1	99%
rt, 24 h, pentane (neat)	2:1.865:1	99:1	98%
reflux, 24 h, pentane	2:1.865:1	99:1	86%
**reflux, 2 h, pentane**	2:1.865:1	**99:1**	**97%**
reflux 2 h, hexane	2:1.865:1	99:1	91%
reflux 18 h, pentane	2:1.865:1	99:1	72%
rt, 24 h, toluene	2:1.865:1	99:1	86%
60 ^oC, 24 h, toluene	2:1.865:1	99:1	94%
60 ^oC, 4 h, toluene	2:1.865:1	99:1	87%
reflux, 24 h, toluene	2:1.865:1	99:1	82%
60 ^oC, 16 h, toluene	2:1.865:1.5	99:1	79%
60 ^oC, 16 h, toluene	1:0.9325:1	94:6	62%

[a]The reactions were carried out using dimethylamine-borane as the amine-borane, and 4-methoxyphenylacetylene as the alkyne. [b]Ratio is of mono- substituted to di- substituted product. [c]Isolated yield, but without complete chromatographic separation. [d]Alkyne conversion includes both mono- and di- substituted products

Table S6. Optimization of Dibromide Conversion

Reaction Conditions	Equivalency DMAB:nBuLi:Dibromide	Product Ratio[b]	Alkyne Conversion[c,d]
1h -78 ^oC, 1h 0 ^oC, 2h reflux	2:3.865:1	99:1	10%
1h -78 ^oC, 1h 0 ^oC, 2h reflux	2:2.865:1	99:1	62%
1h -78 ^oC, 1h 0 ^oC, 4h reflux	2:2.865:1	99:1	60%
1h -78 ^oC, 1h 0 ^oC, 2h reflux	2:2.3:1	99:1	89%

NMR spectra of amine-boranes

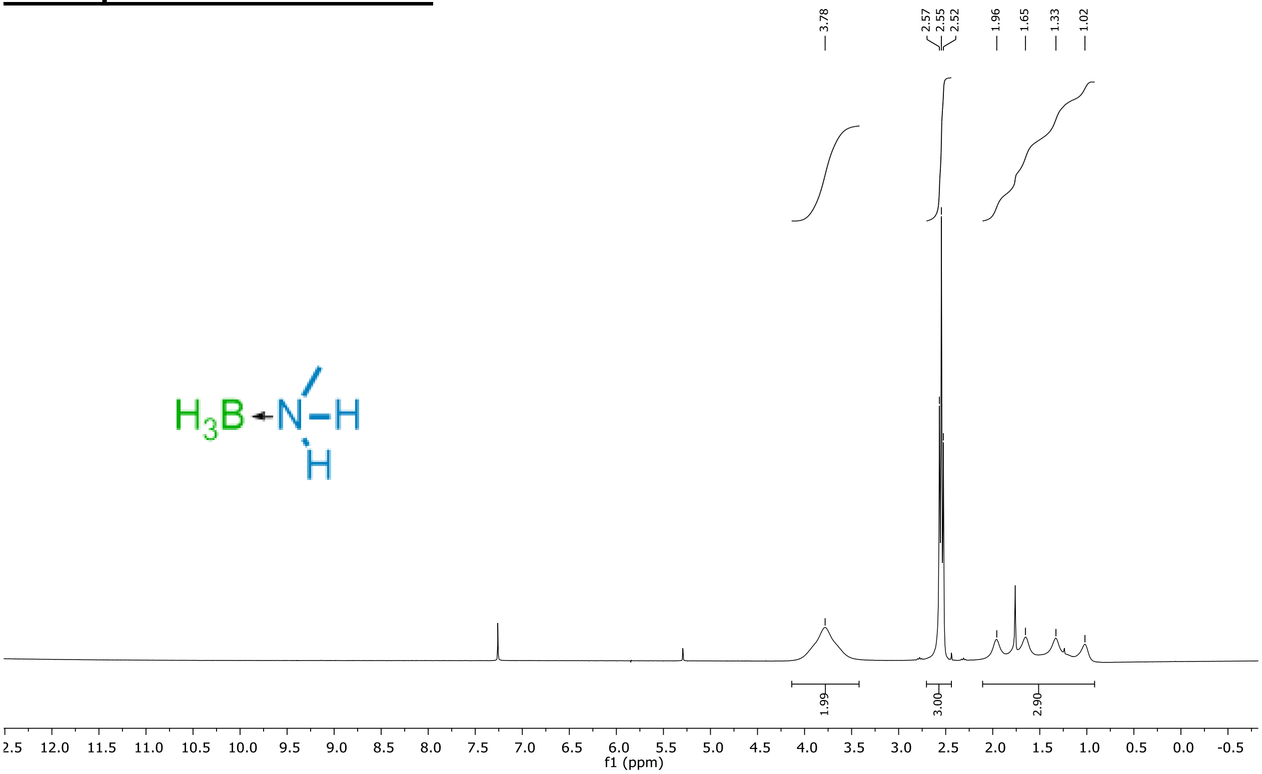

^{1}H NMR (300 MHz, $CDCl_3$) methylamine-borane (**2a**)

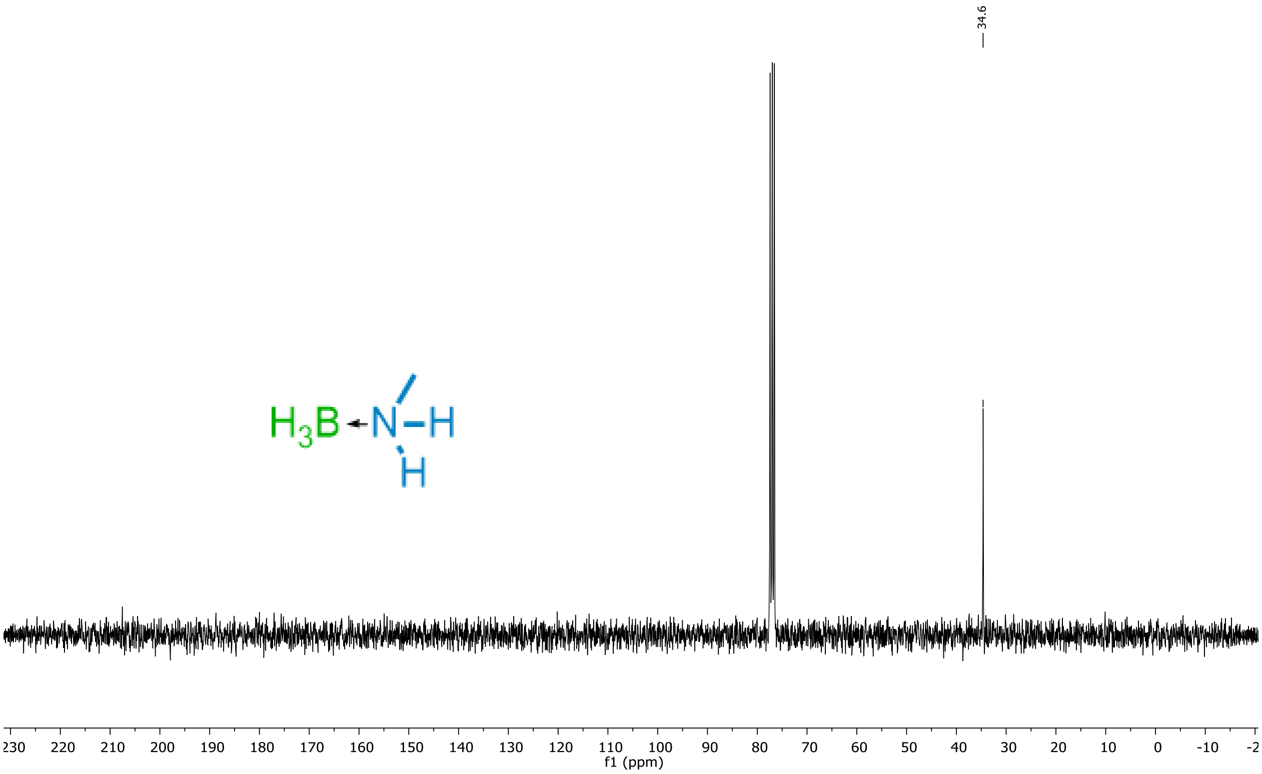

^{13}C NMR (75 MHz, $CDCl_3$) methylamine-borane (**2a**)

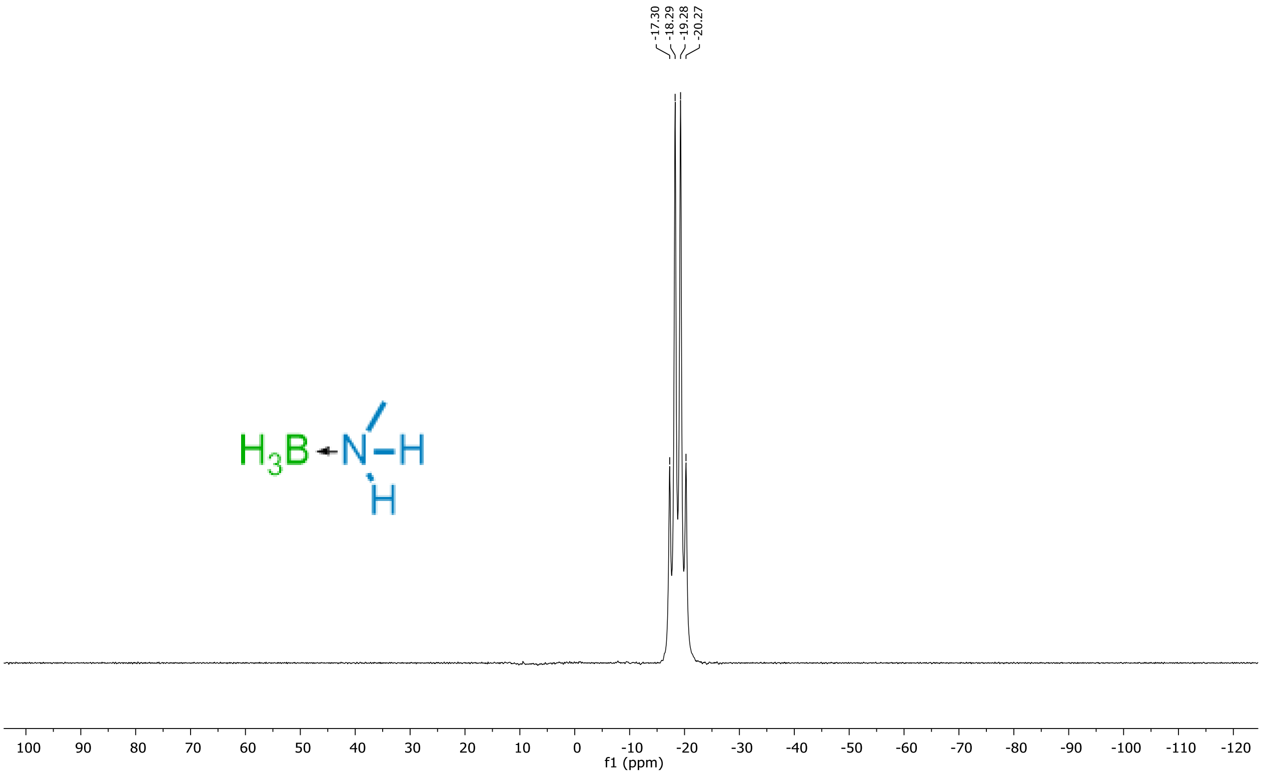

^{11}B NMR (96 MHz, $CDCl_3$) methylamine-borane (**2a**)

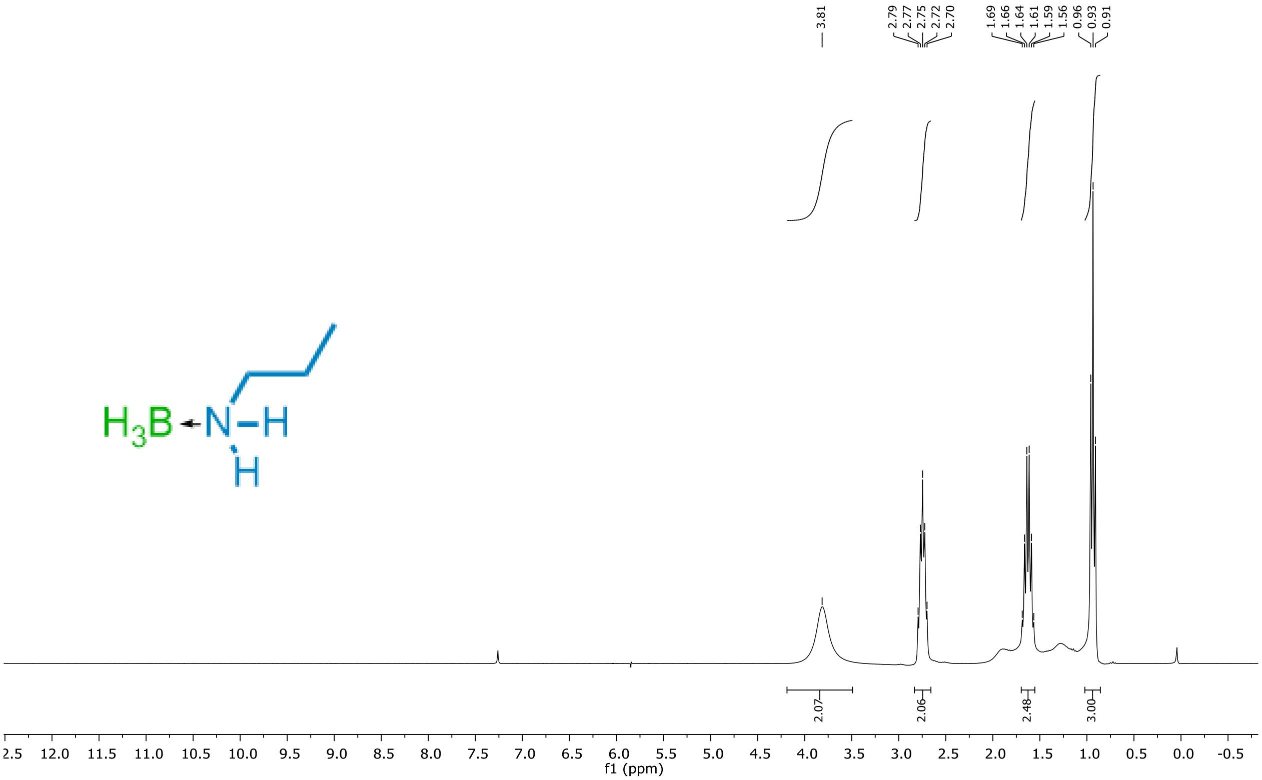

^{1}H NMR (300 MHz, $CDCl_3$) propylamine-borane (**2b**)

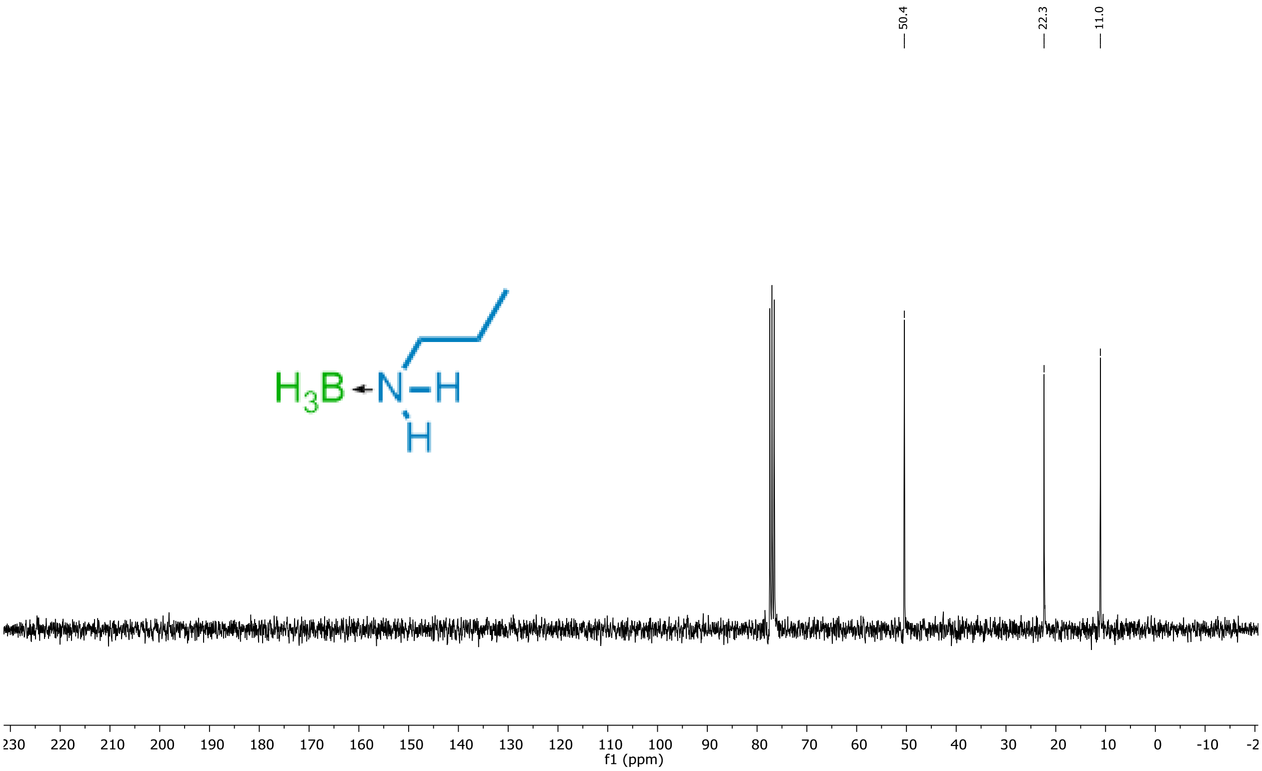

^{13}C NMR (75 MHz, $CDCl_3$) propylamine-borane (**2b**)

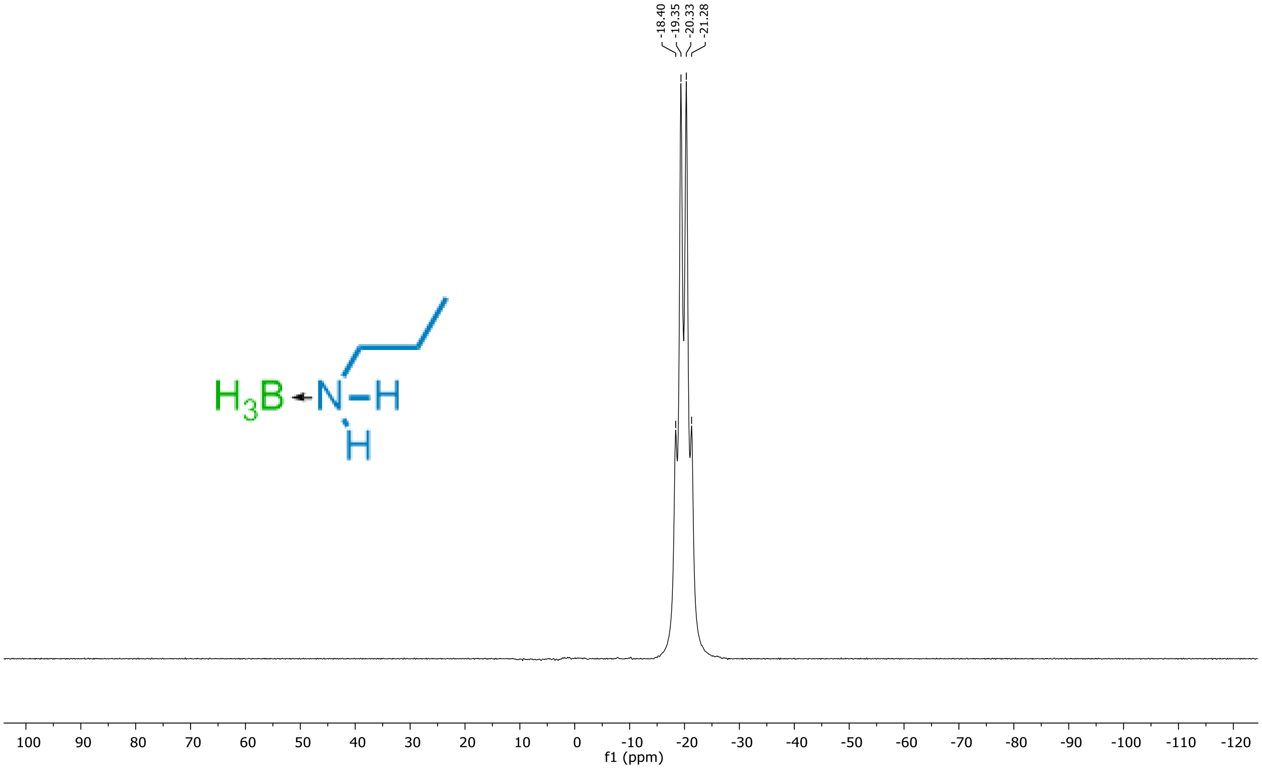

^{11}B NMR (96 MHz, $CDCl_3$) propylamine-borane (**2b**)

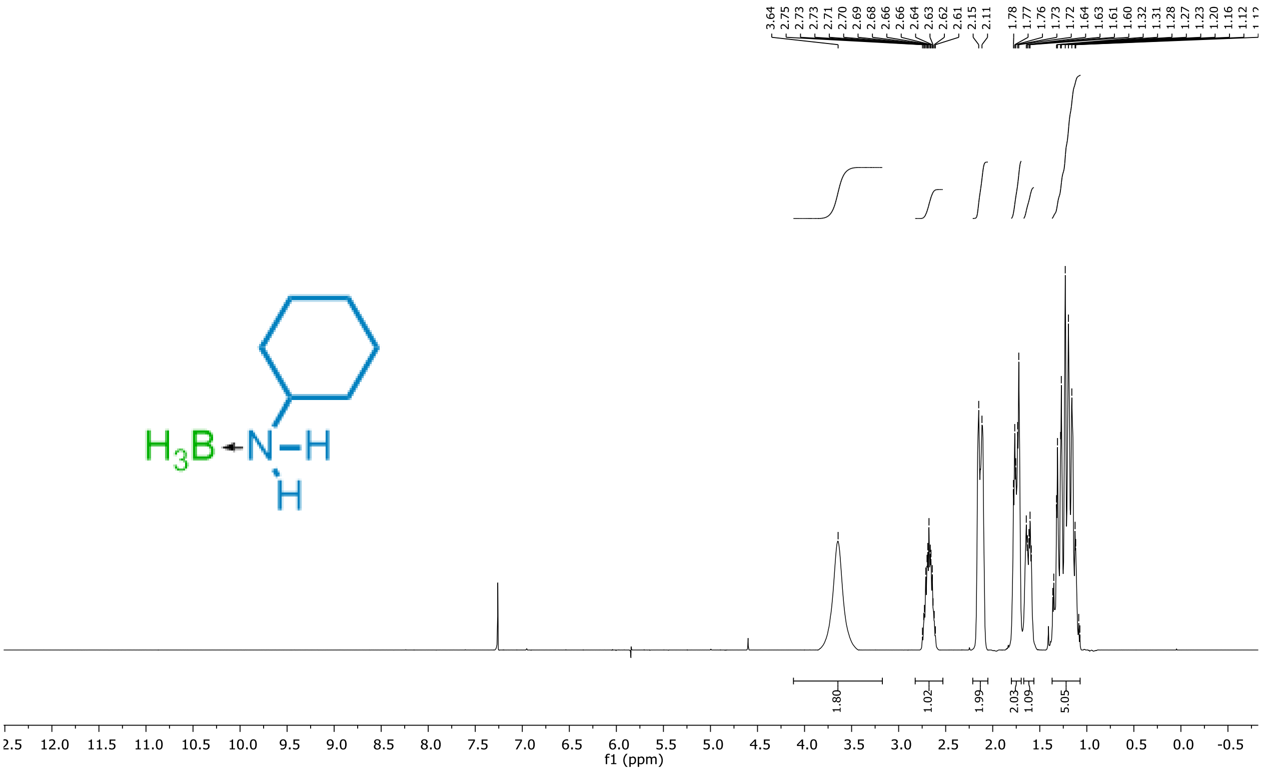

^{1}H NMR (300 MHz, $CDCl_3$) cyclohexylamine-borane (**2c**)

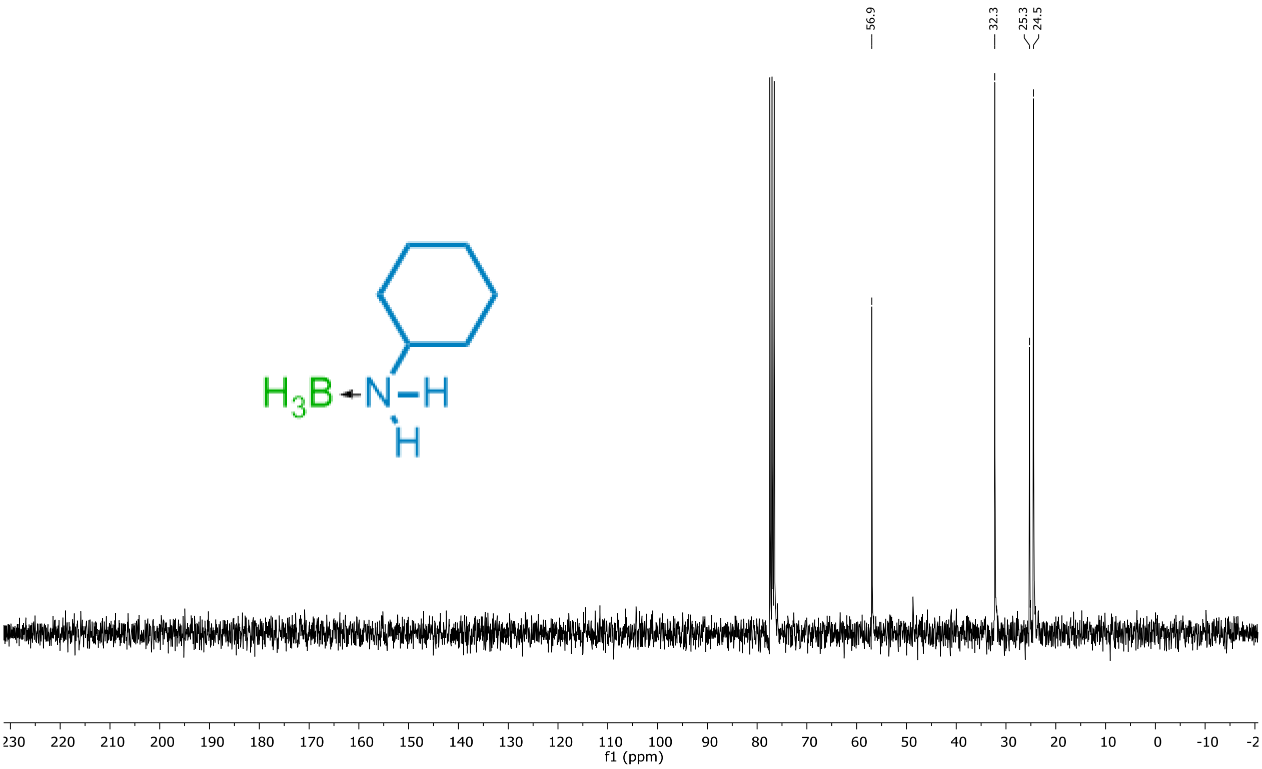

^{13}C NMR (75 MHz, $CDCl_3$) cyclohexylamine-borane (**2c**)

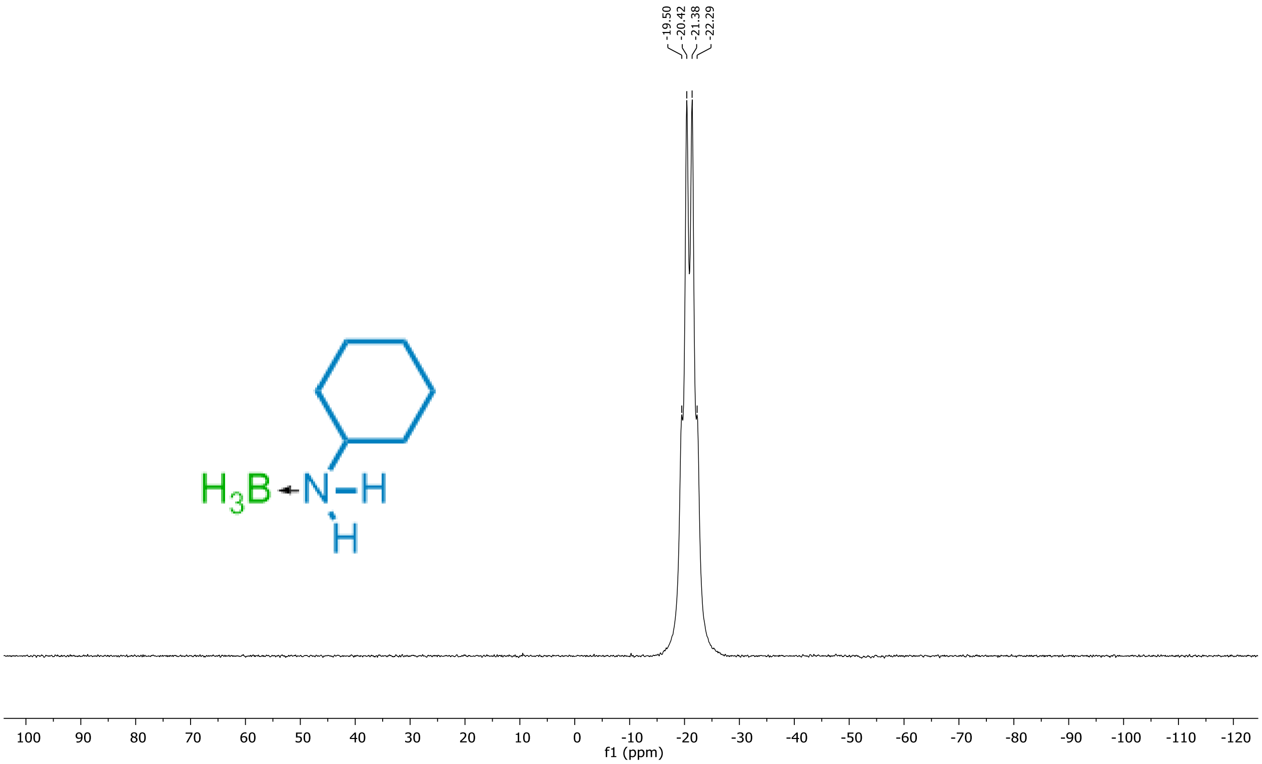

^{11}B NMR (96 MHz, $CDCl_3$) cyclohexylamine-borane (**2c**)

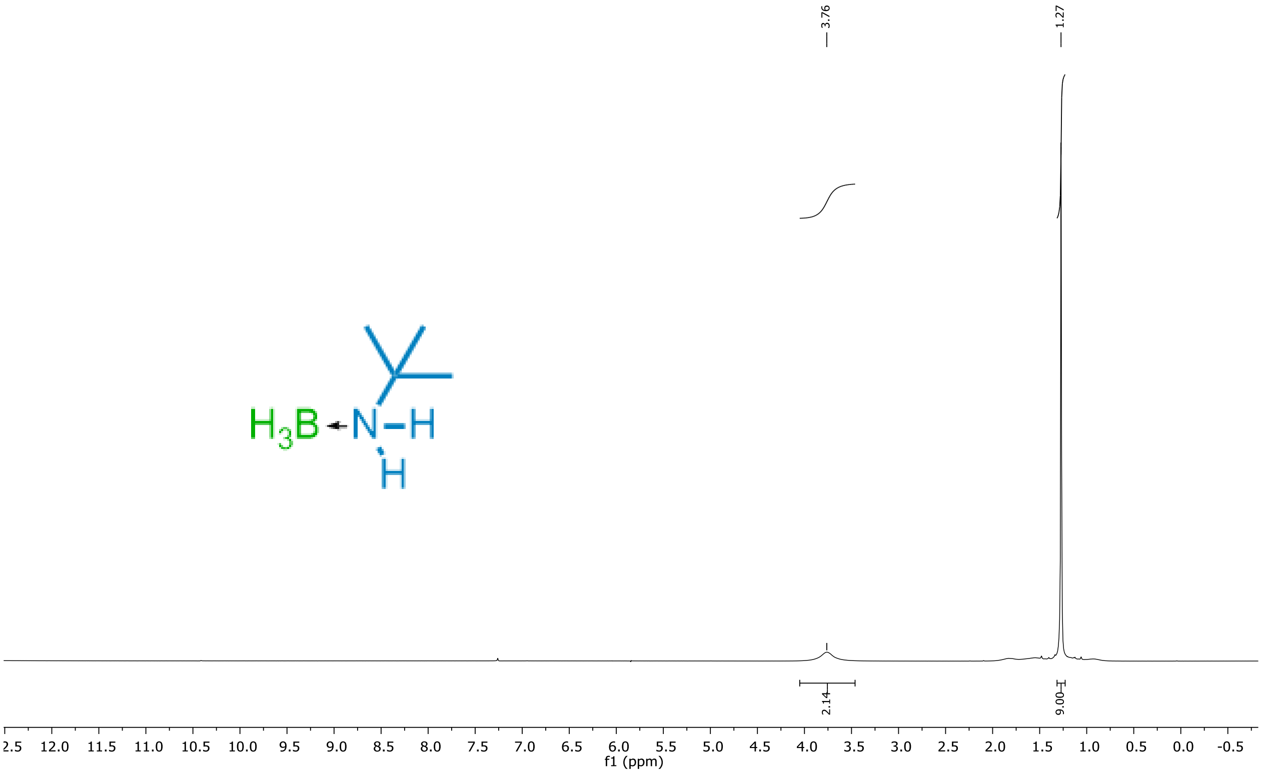

^{1}H NMR (300 MHz, $CDCl_3$) t-butylamine-borane (**2d**)

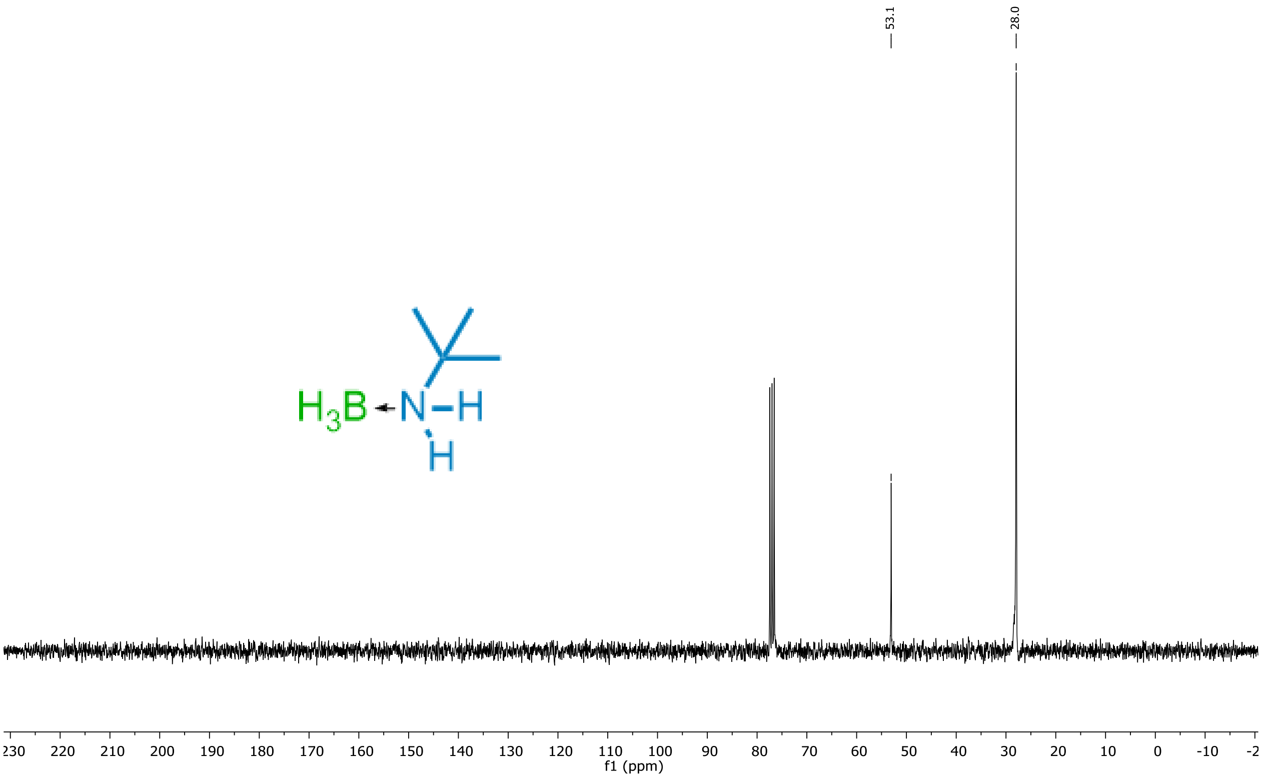

^{13}C NMR (75 MHz, $CDCl_3$) t-butylamine-borane (**2d**)

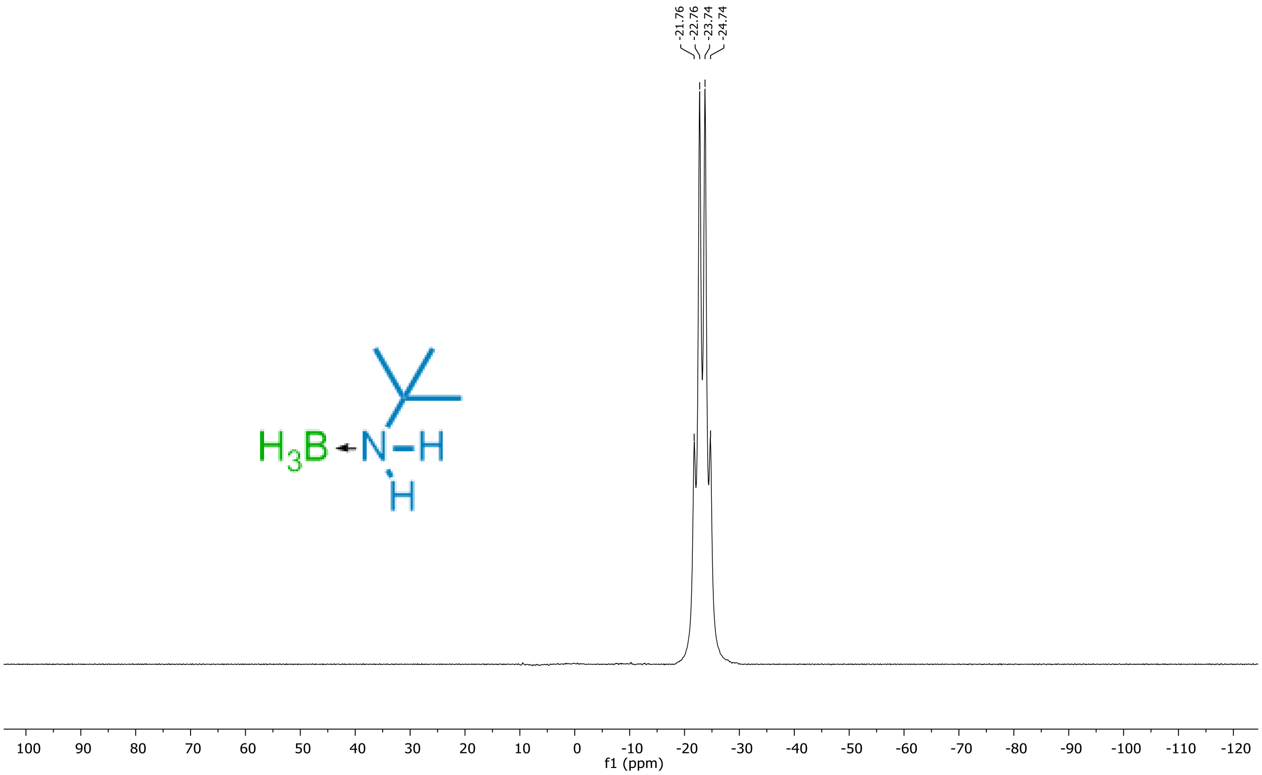

^{11}B NMR (96 MHz, $CDCl_3$) t-butylamine-borane (**2d**)

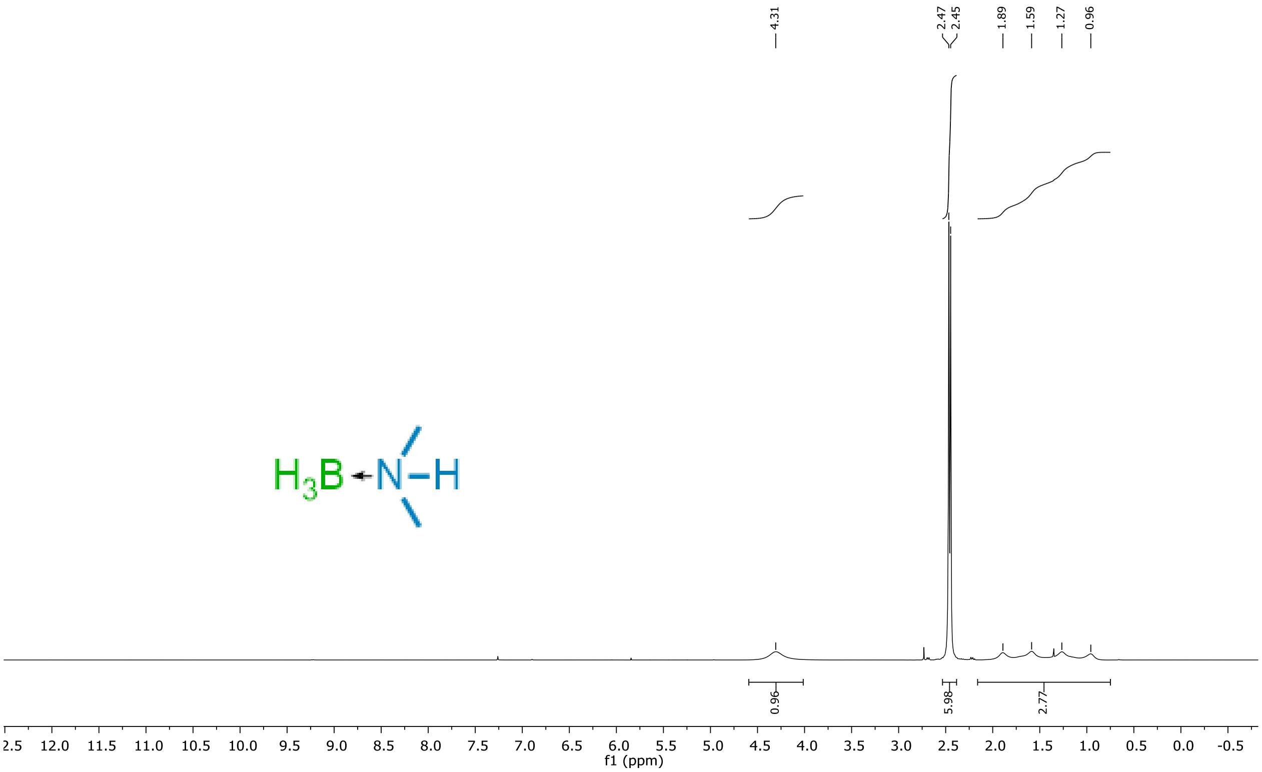

^{1}H NMR (300 MHz, $CDCl_3$) dimethylamine-borane (**2e**)

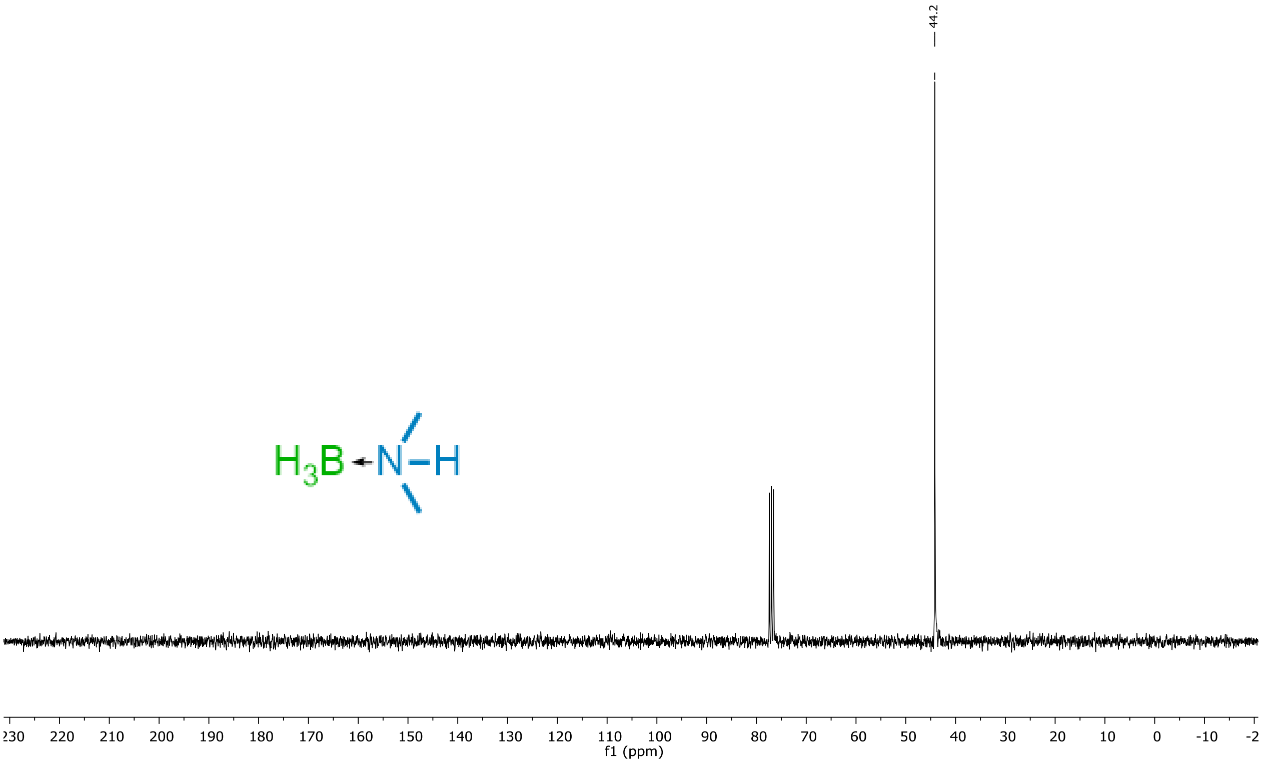

^{13}C NMR (75 MHz, $CDCl_3$) dimethylamine-borane (**2e**)

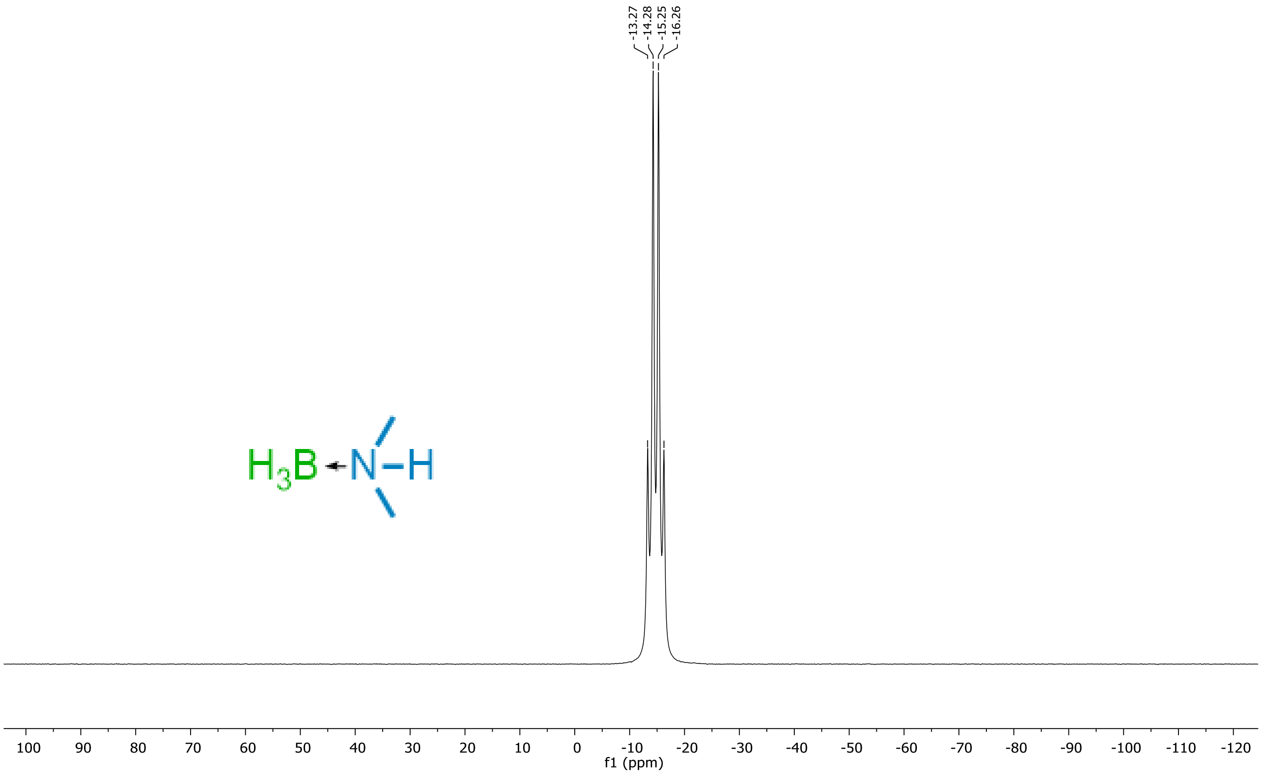

^{11}B NMR (96 MHz, $CDCl_3$) dimethylamine-borane (**2e**)

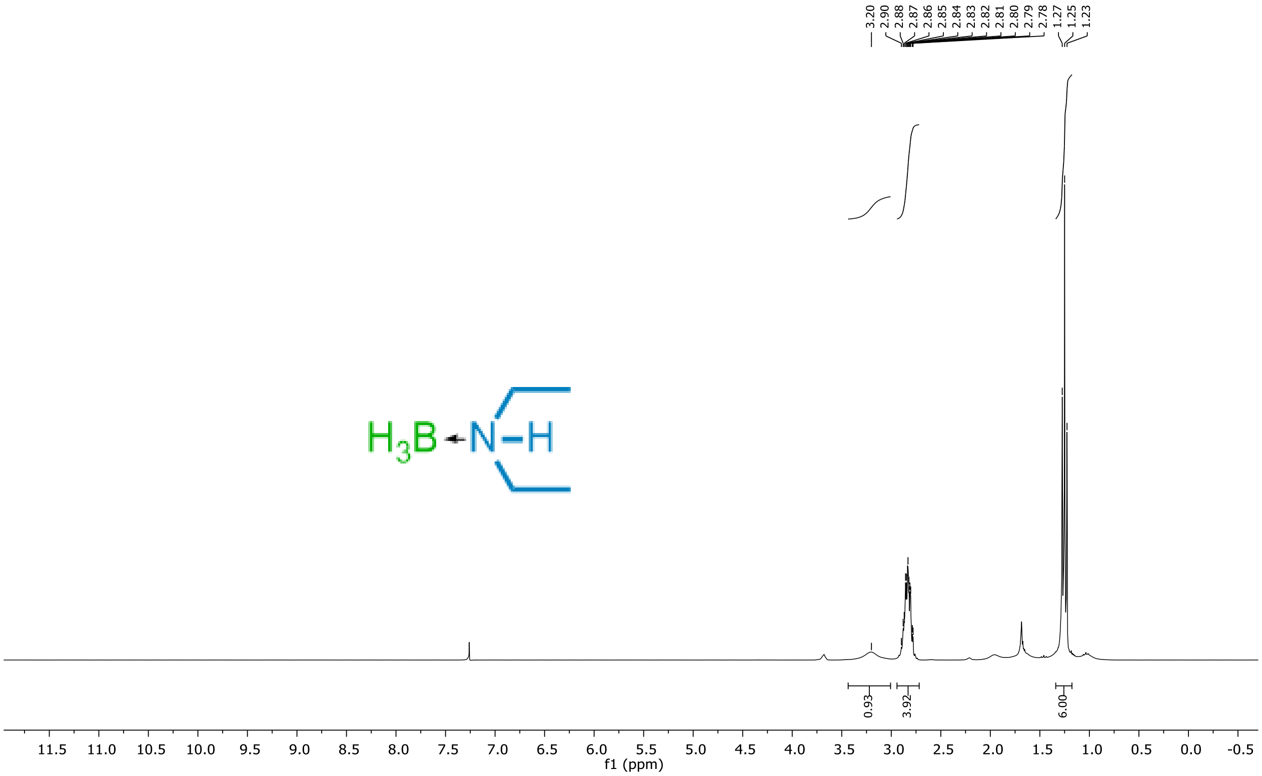

^{1}H NMR (300 MHz, $CDCl_3$) diethylamine-borane (**2f**)

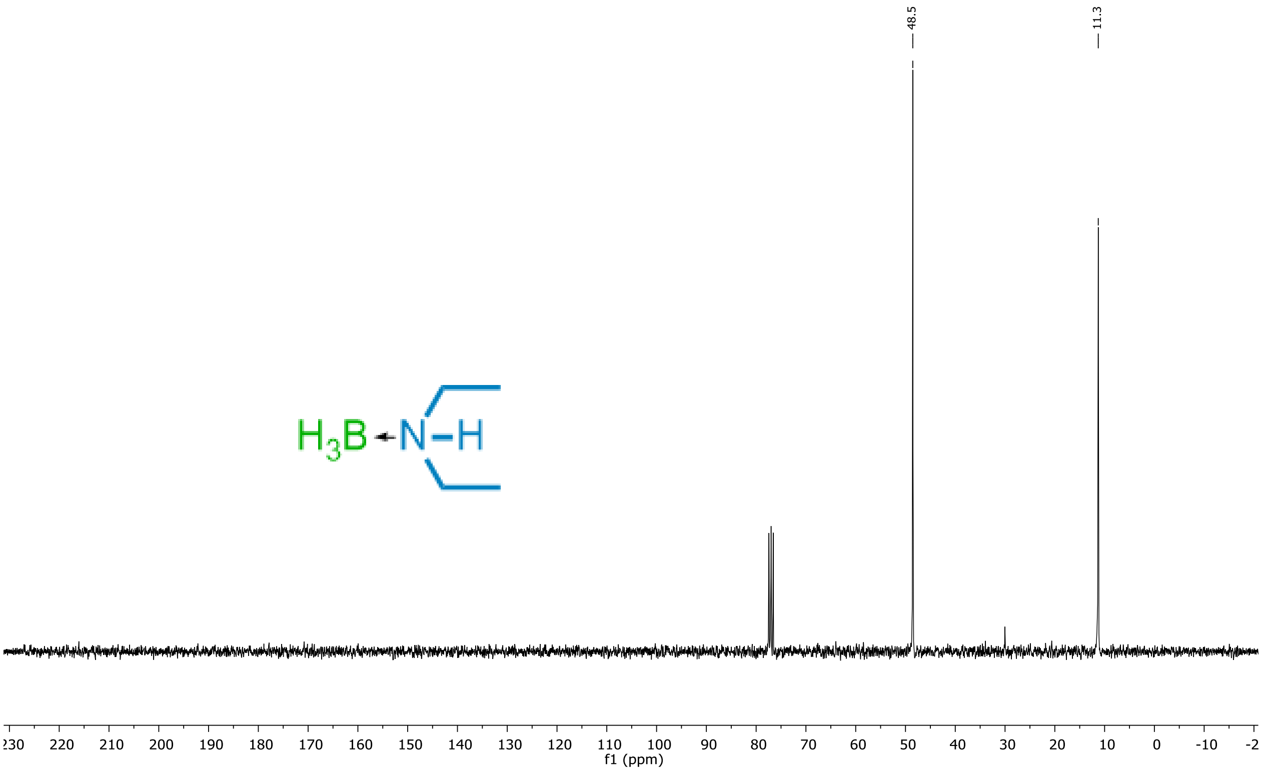

^{13}C NMR (75 MHz, $CDCl_3$) diethylamine-borane (**2f**)

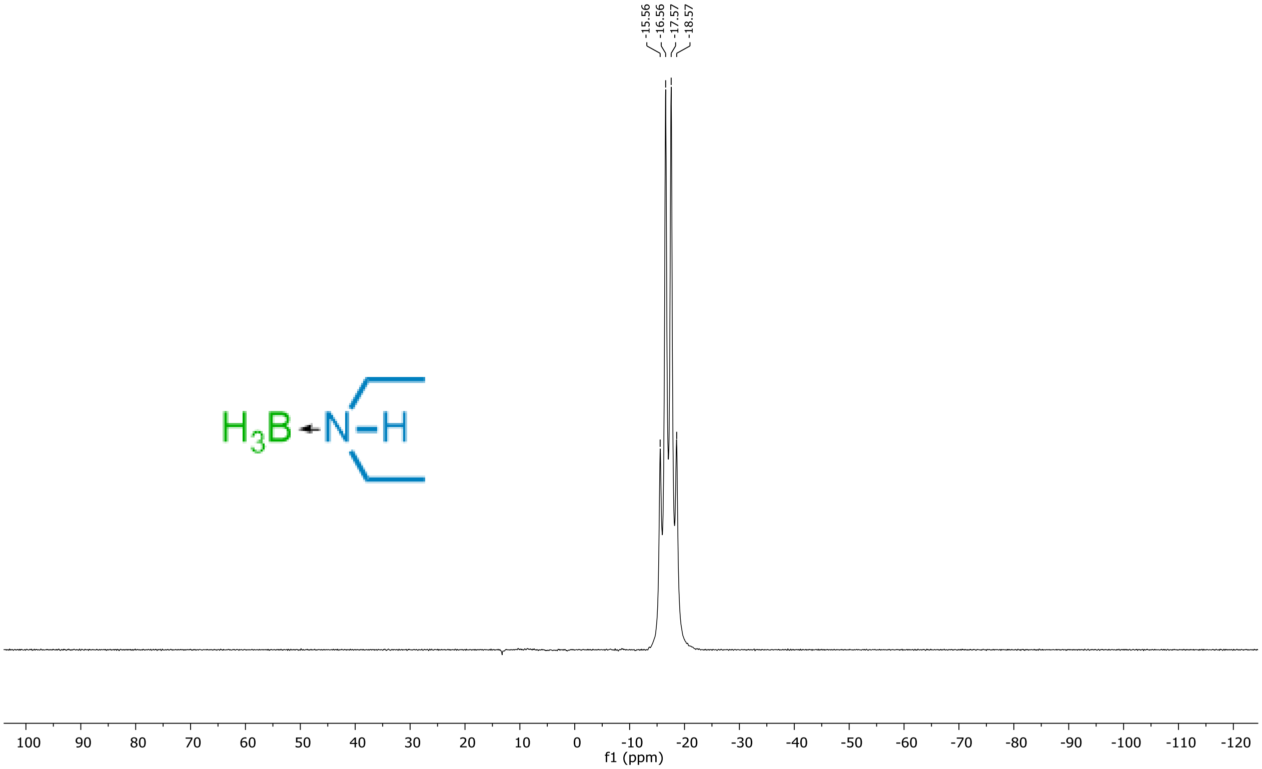

^{11}B NMR (96 MHz, $CDCl_3$) diethylamine-borane (**2f**)

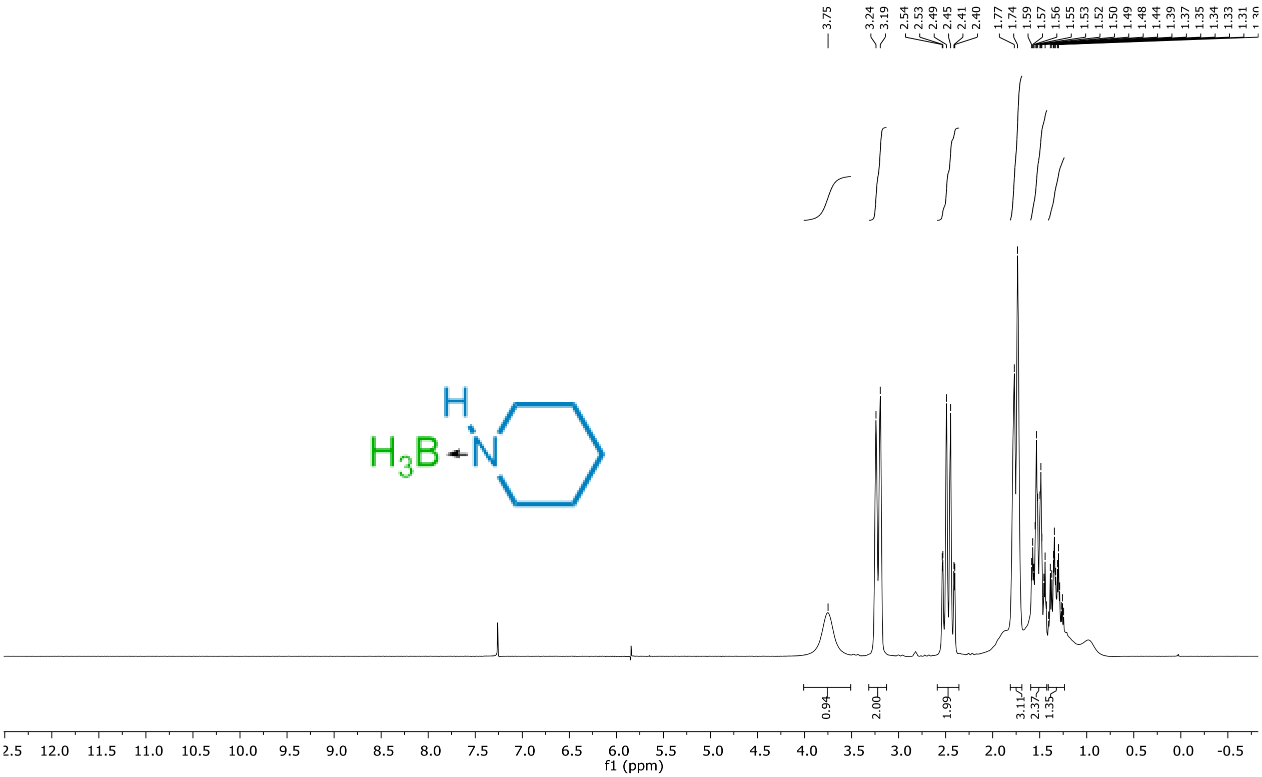

^{1}H NMR (300 MHz, $CDCl_3$) piperidine-borane (**2g**)

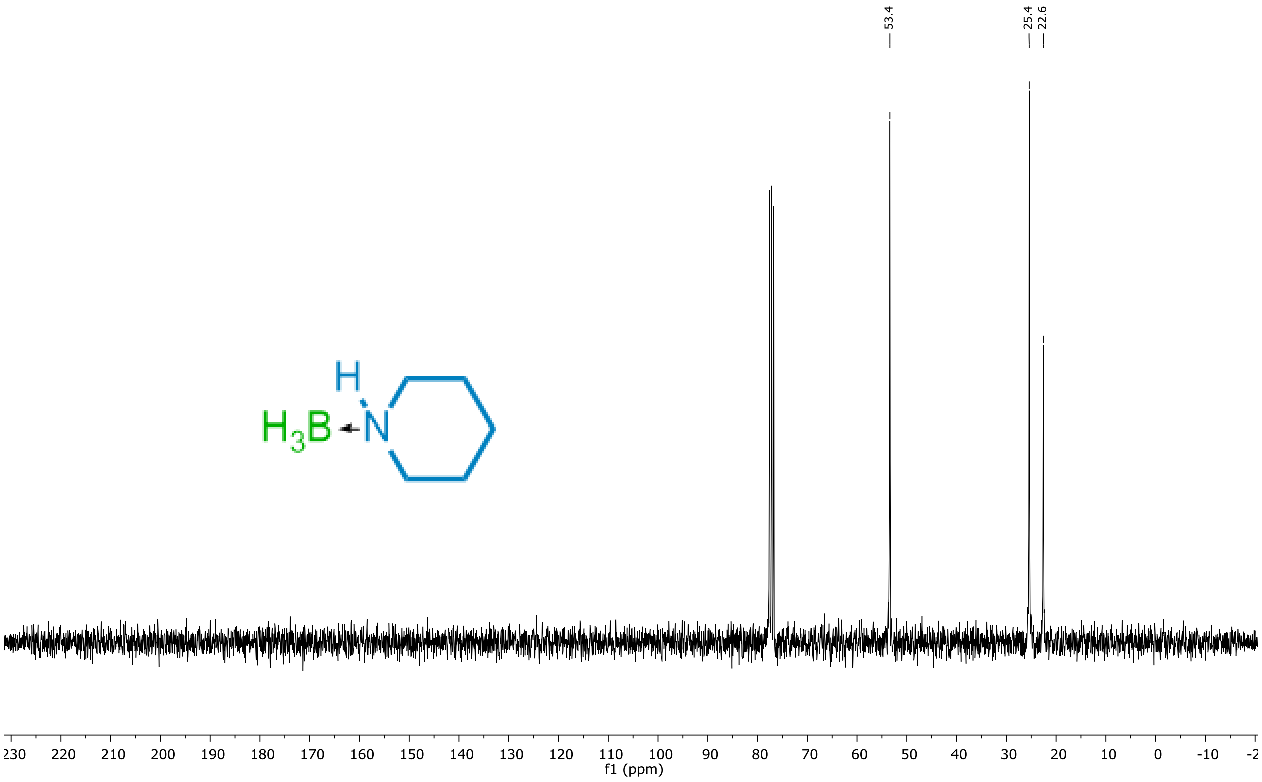

^{13}C NMR (75 MHz, $CDCl_3$) piperidine-borane (**2g**)

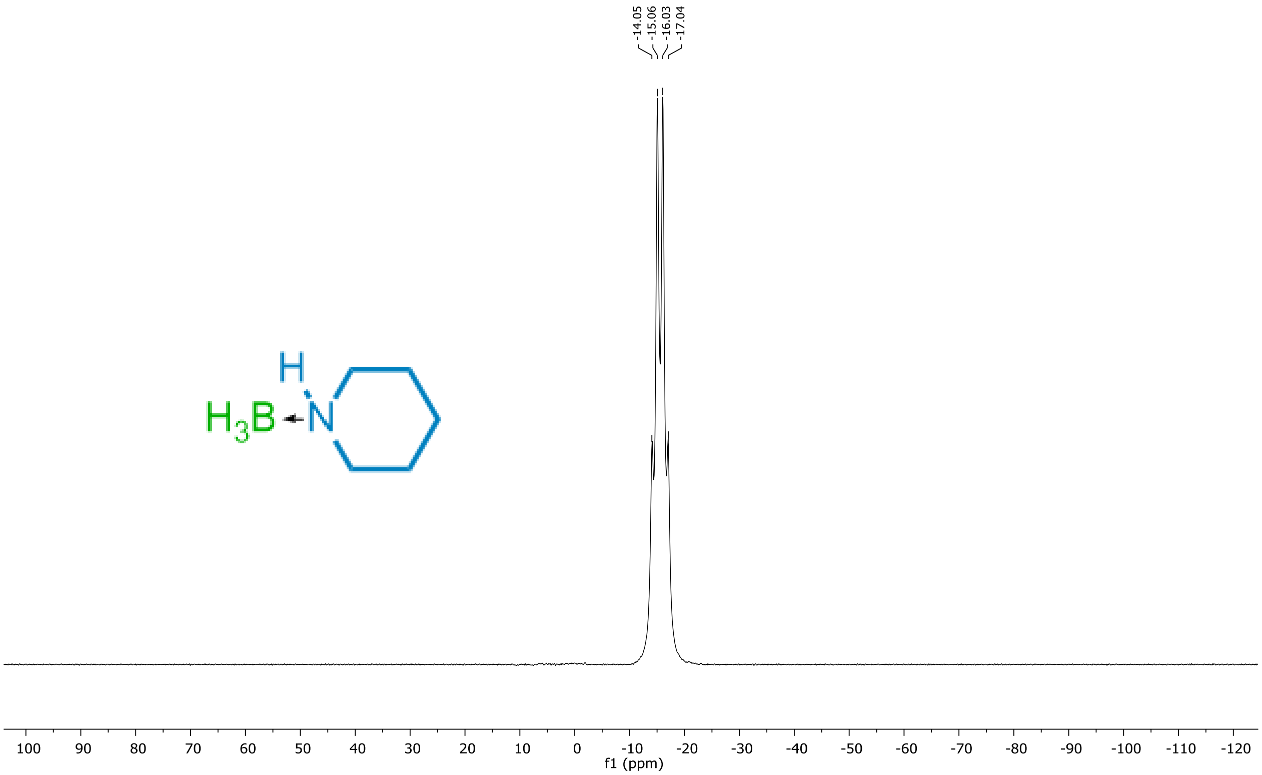

^{11}B NMR (96 MHz, $CDCl_3$) piperidine-borane (**2g**)

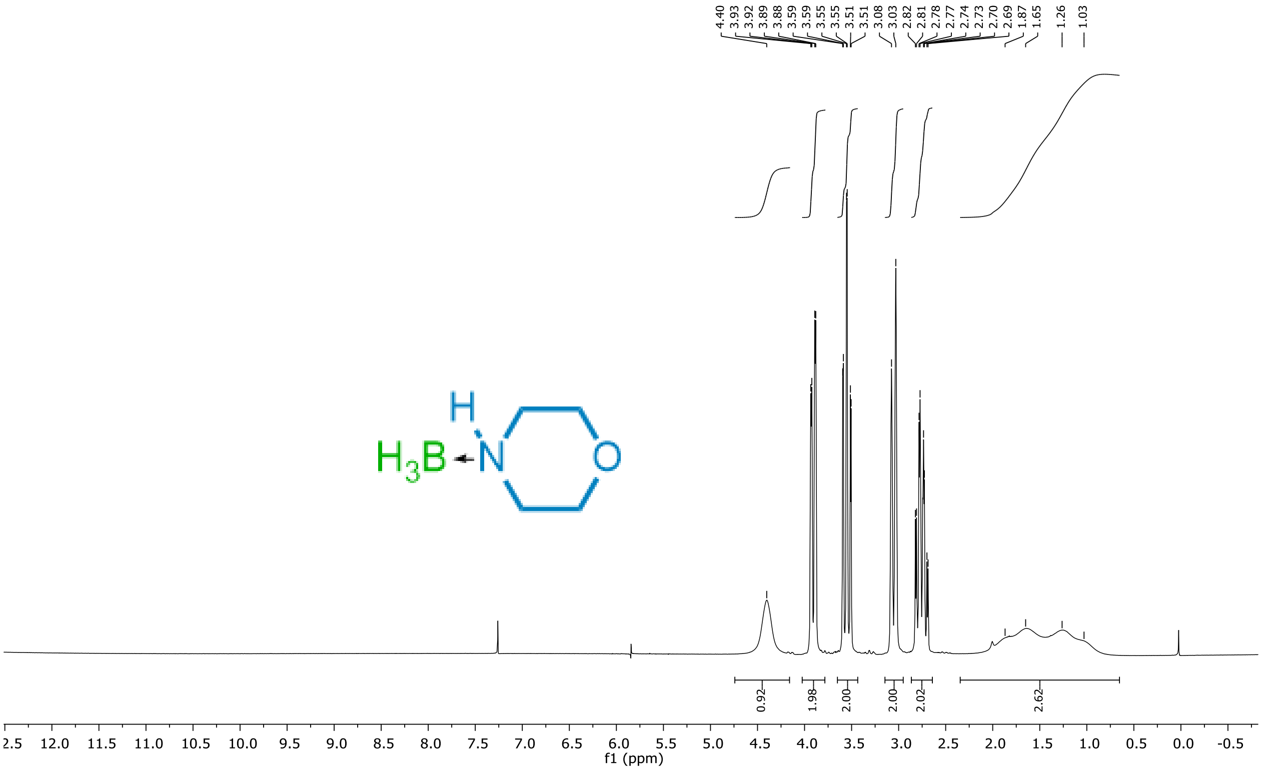

^{1}H NMR (300 MHz, $CDCl_3$) morpholine-borane (**2h**)

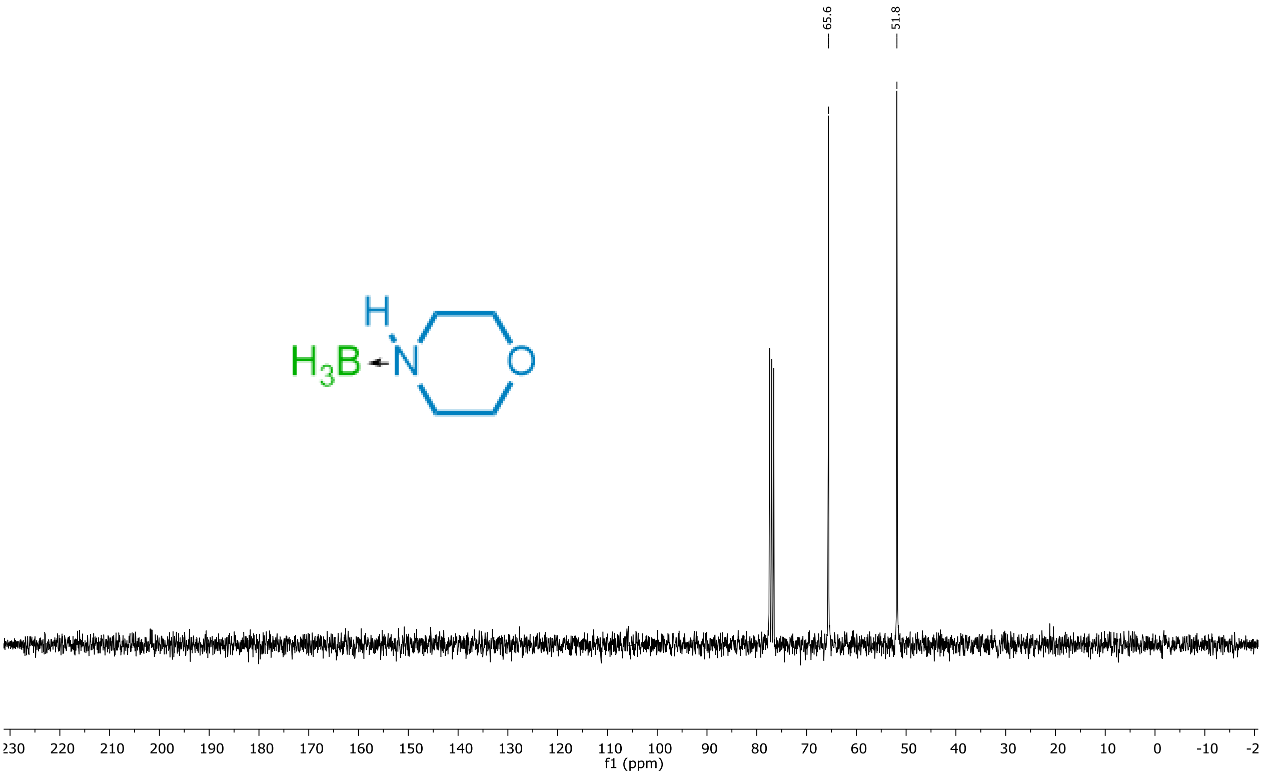

^{13}C NMR (75 MHz, $CDCl_3$) morpholine-borane (**2h**)

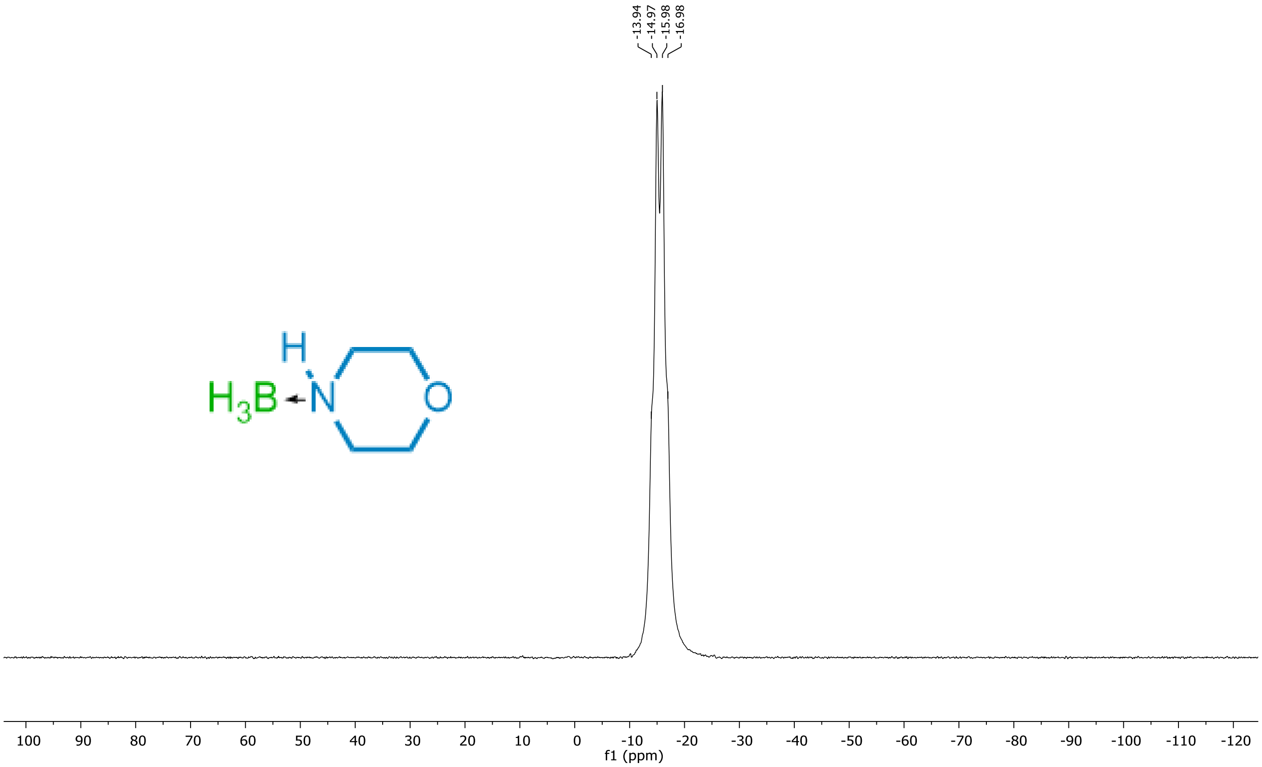

^{11}B NMR (96 MHz, $CDCl_3$) morpholine-borane (**2h**)

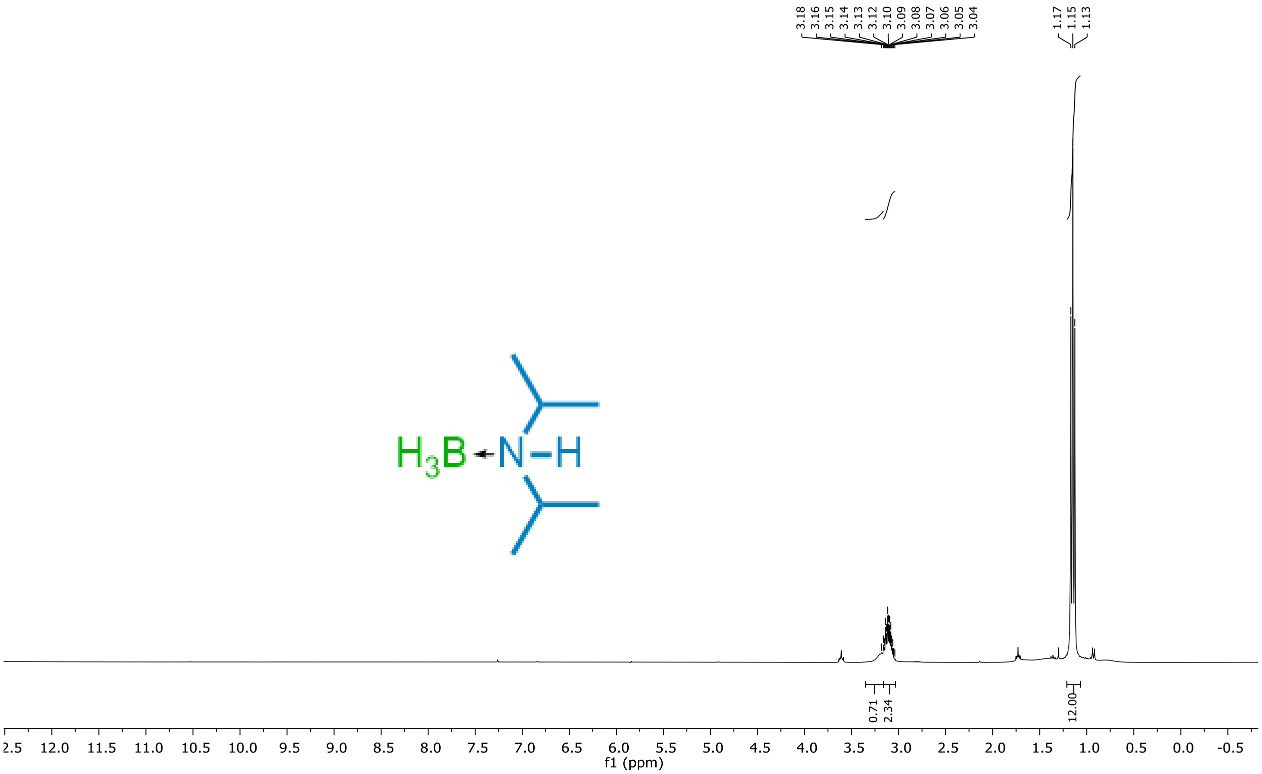

^{1}H NMR (300 MHz, $CDCl_3$) diisopropylamine-borane (**2i**)

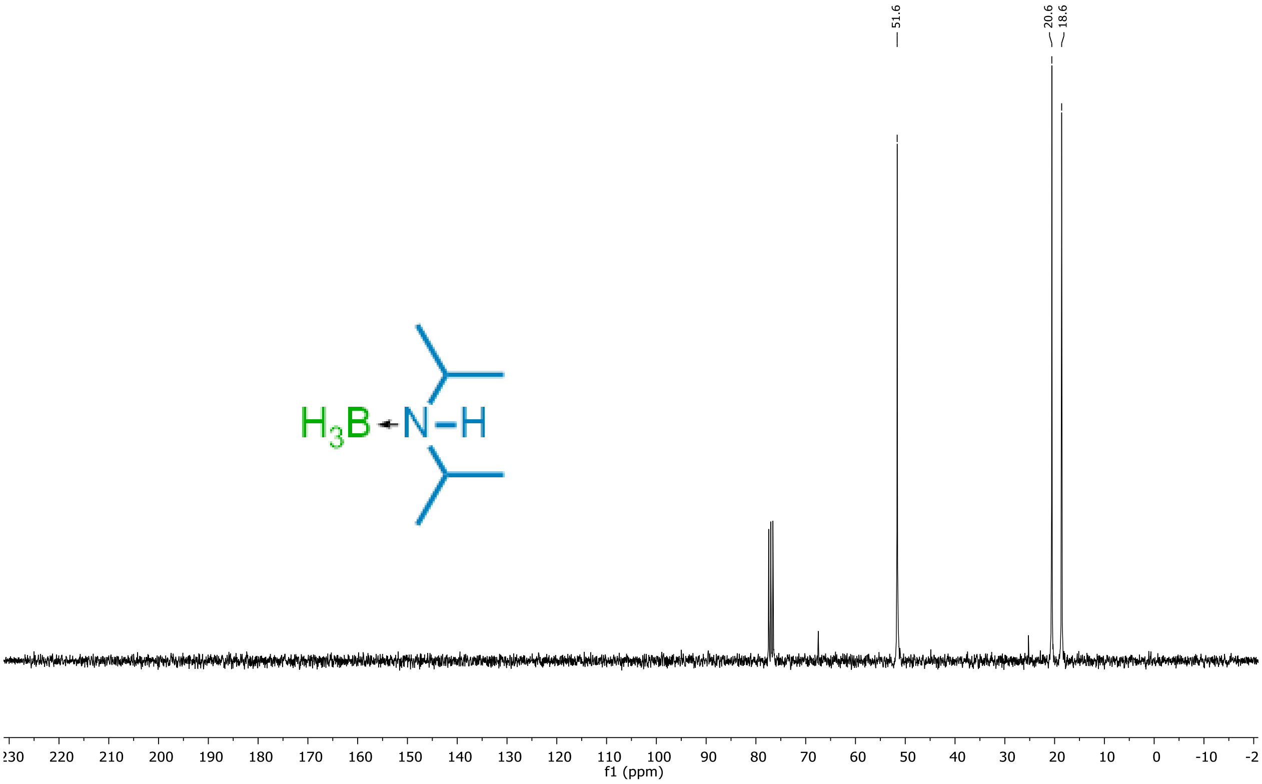

^{13}C NMR (75 MHz, $CDCl_3$) diisopropylamine-borane (**2i**)

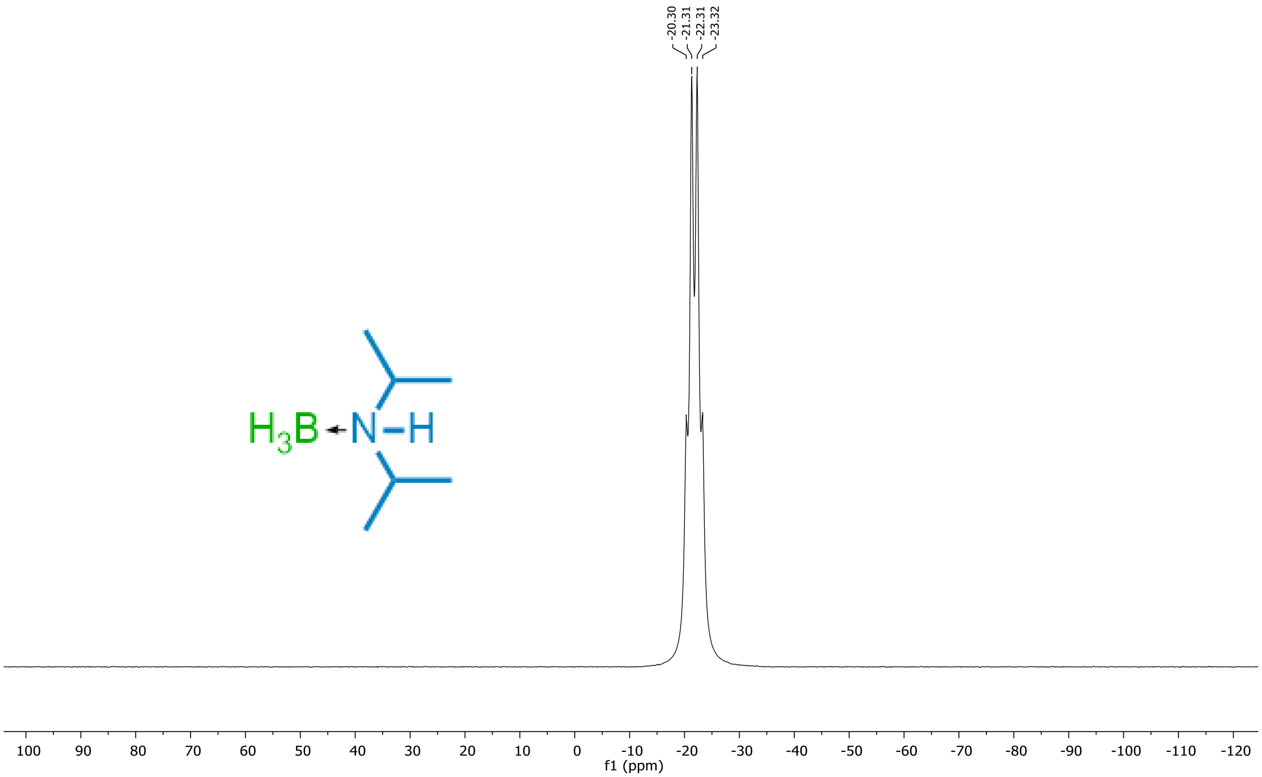

^{11}B NMR (96 MHz, $CDCl_3$) diisopropylamine-borane (**2i**)

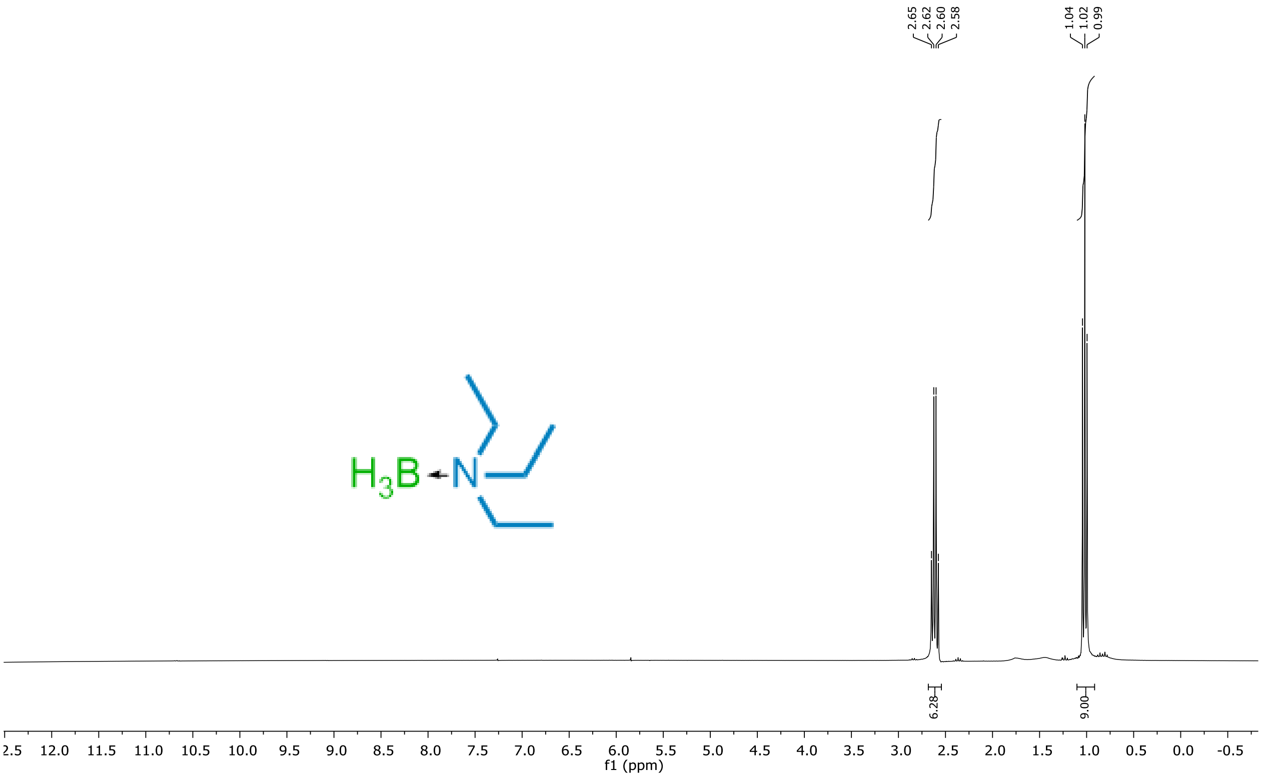

^{1}H NMR (300 MHz, $CDCl_3$) triethylamine-borane (**2j**)

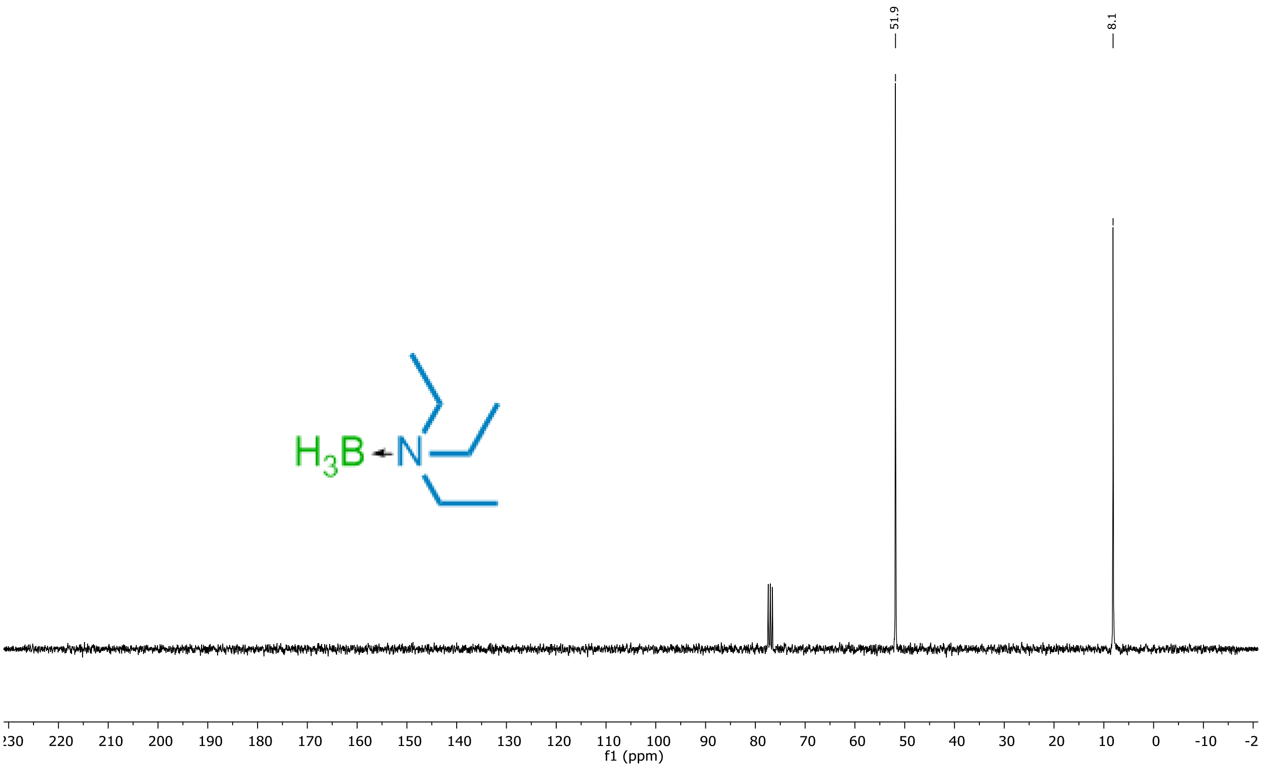

^{13}C NMR (75 MHz, $CDCl_3$) triethylamine-borane (**2j**)

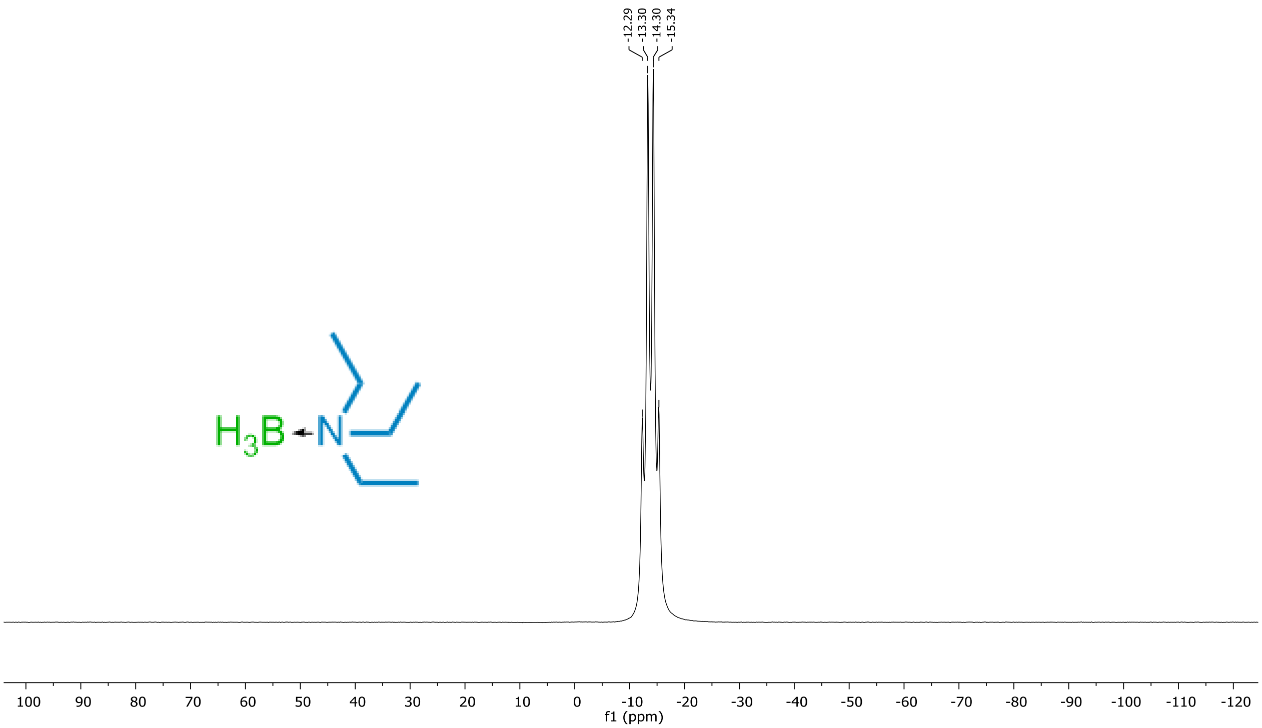

^{11}B NMR (96 MHz, $CDCl_3$) triethylamine-borane (**2j**)

<u>NMR spectra of alkynylborane-amines from terminal alkynes</u>

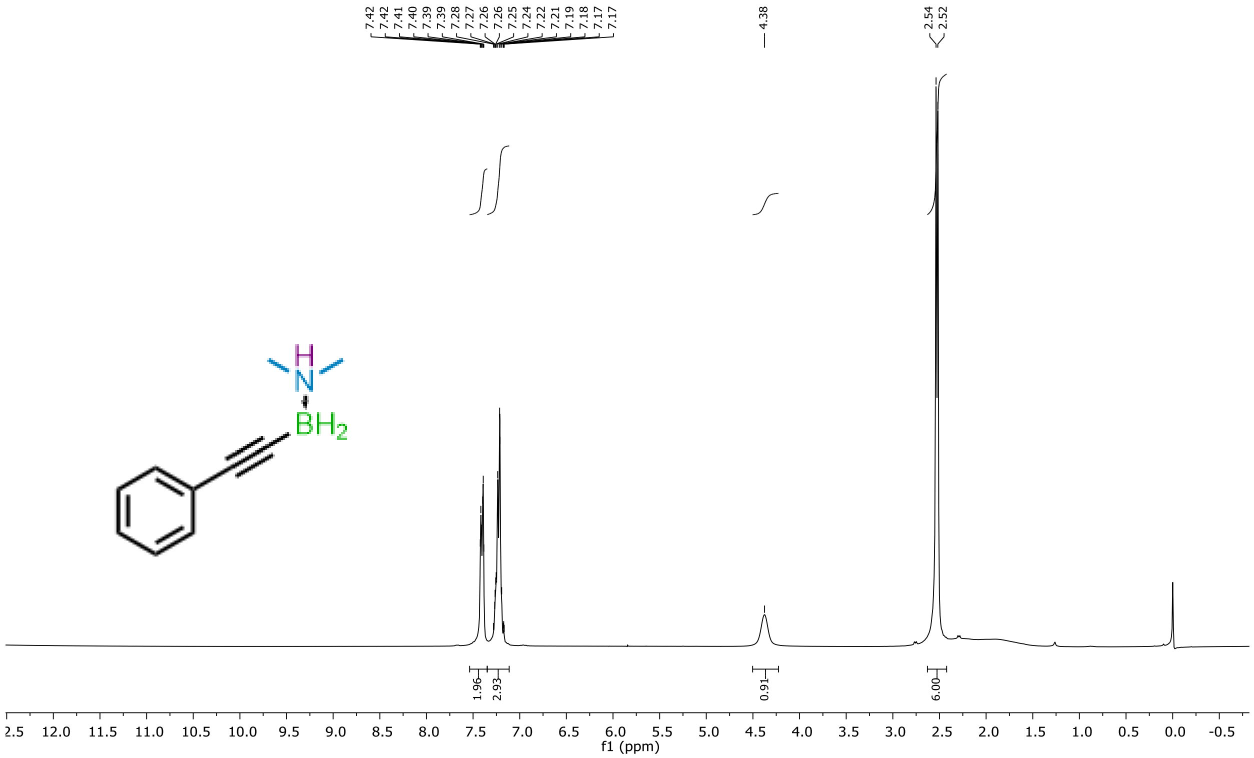

^{1}H NMR (300 MHz, $CDCl_3$) (phenylethynyl)borane-dimethylamine (**3ae**)

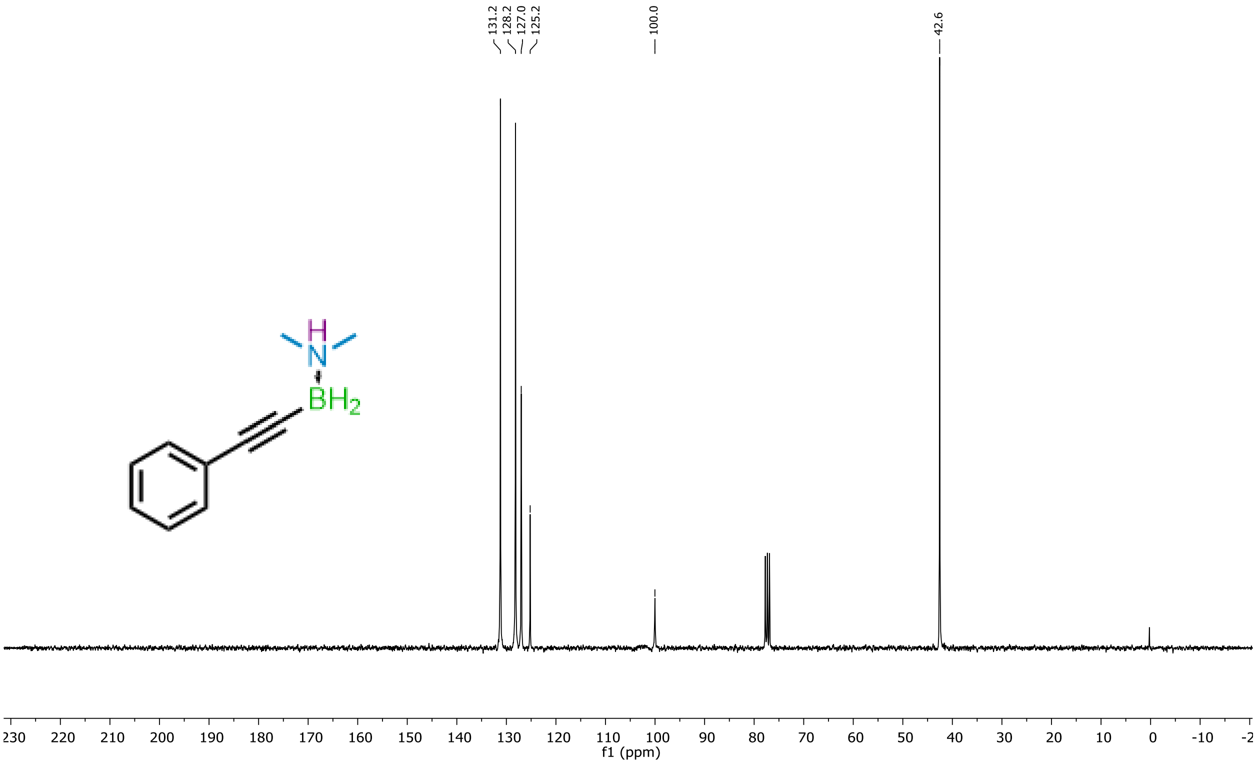

^{13}C NMR (75 MHz, $CDCl_3$) (phenylethynyl)borane-dimethylamine (**3ae**)

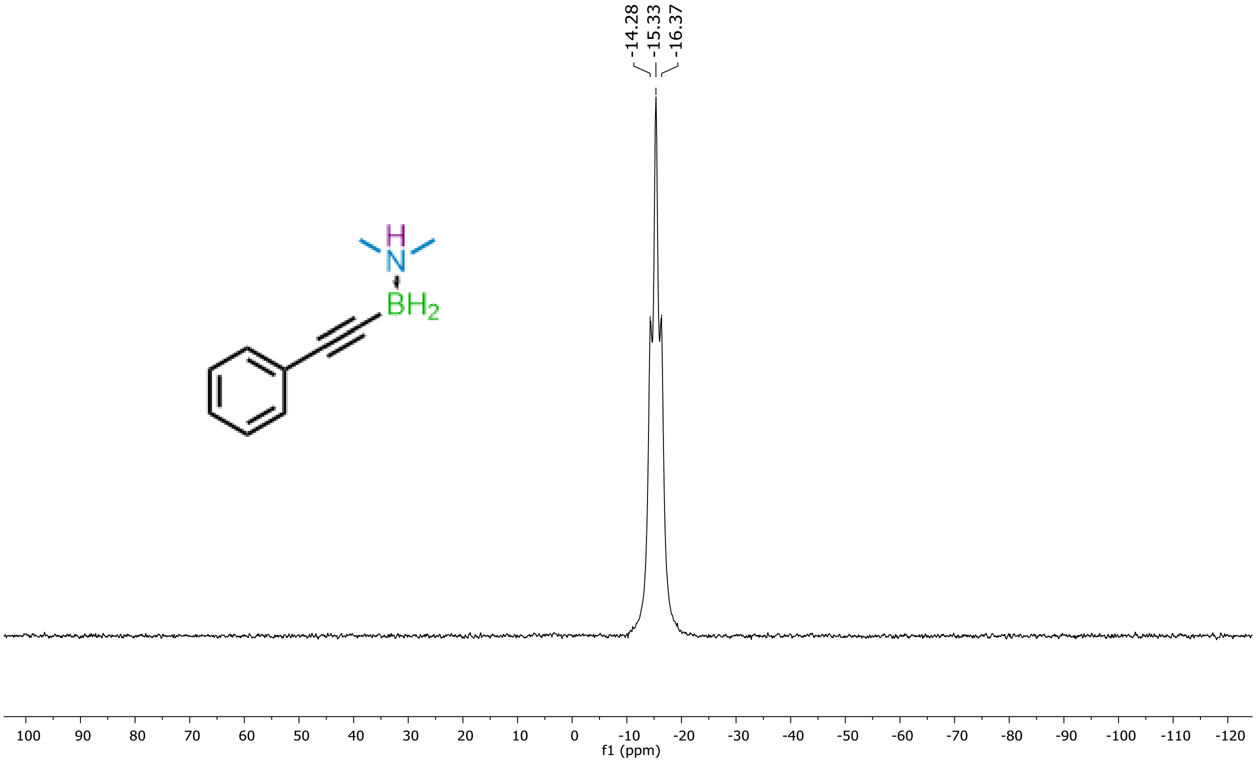

^{11}B NMR (96 MHz, $CDCl_3$) (phenylethynyl)borane-dimethylamine (**3ae**)

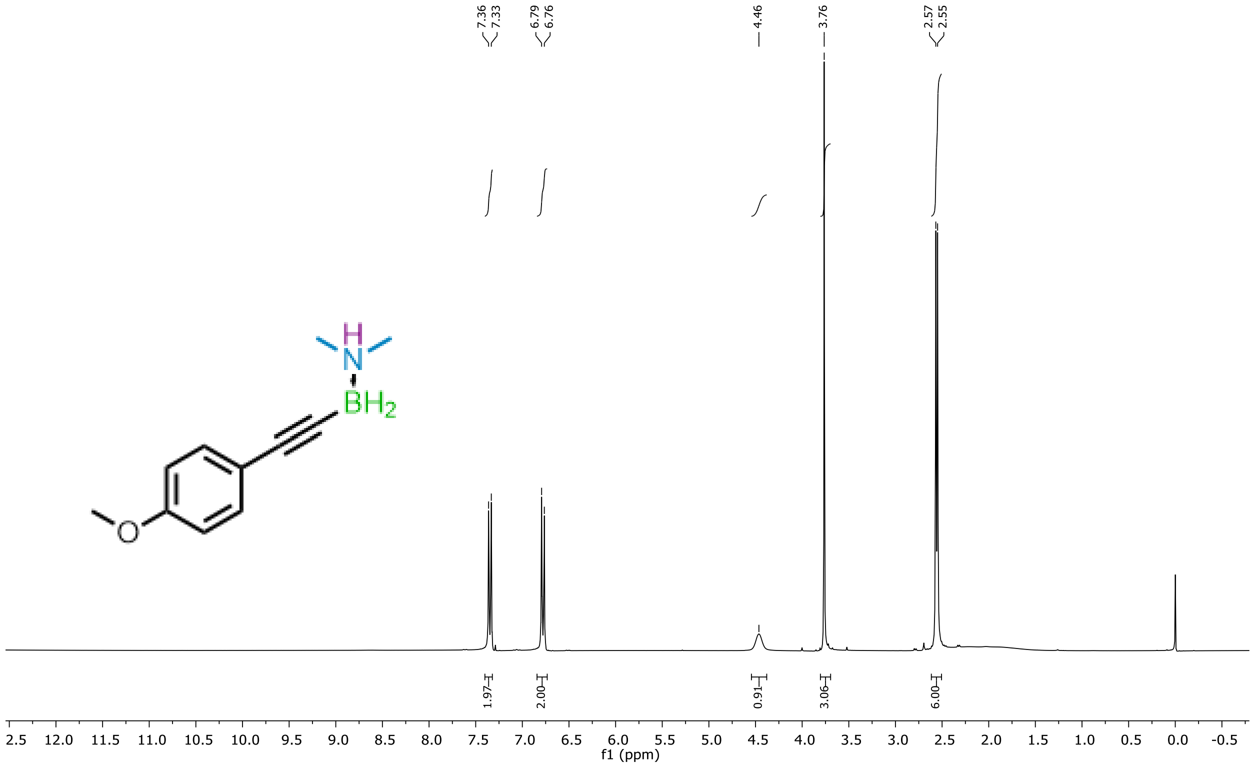

^{1}H NMR (300 MHz, $CDCl_3$) ((4-methoxyphenyl)ethynyl)borane-dimethylamine (**3be**)

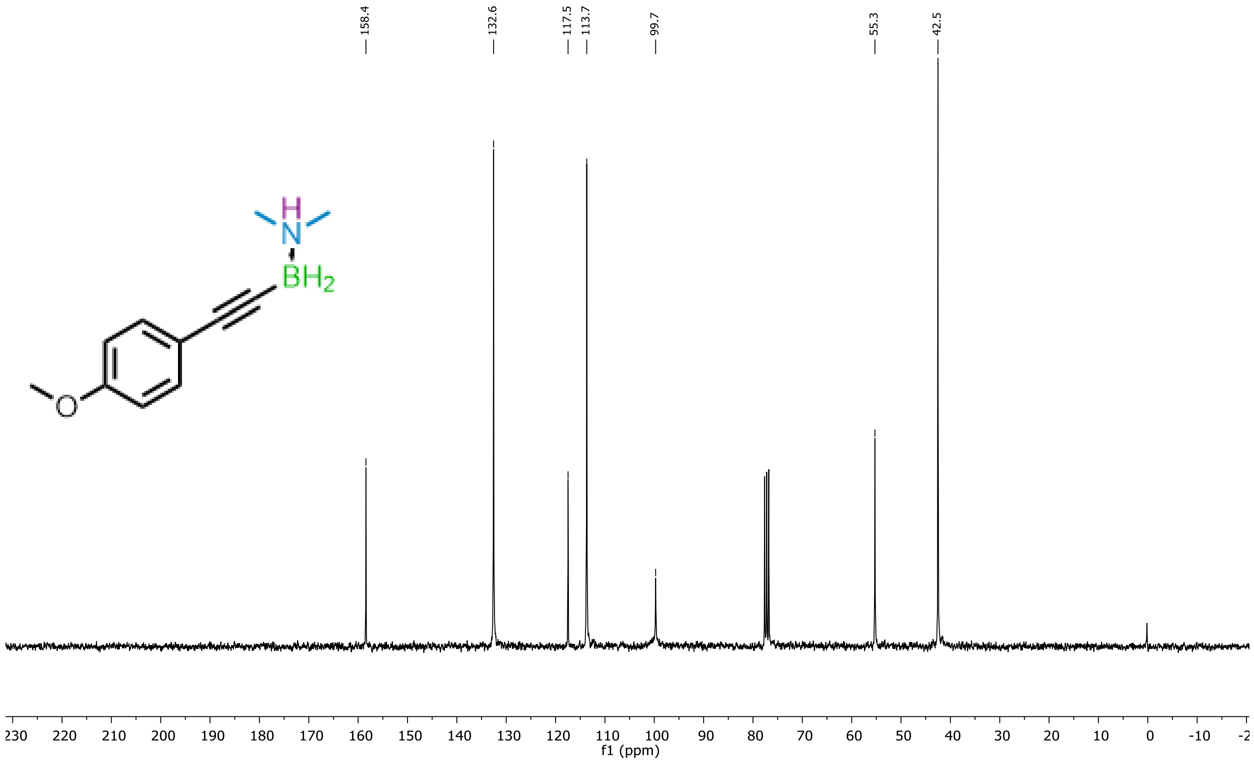

^{13}C NMR (75 MHz, $CDCl_3$) ((4-methoxyphenyl)ethynyl)borane-dimethylamine (**3be**)

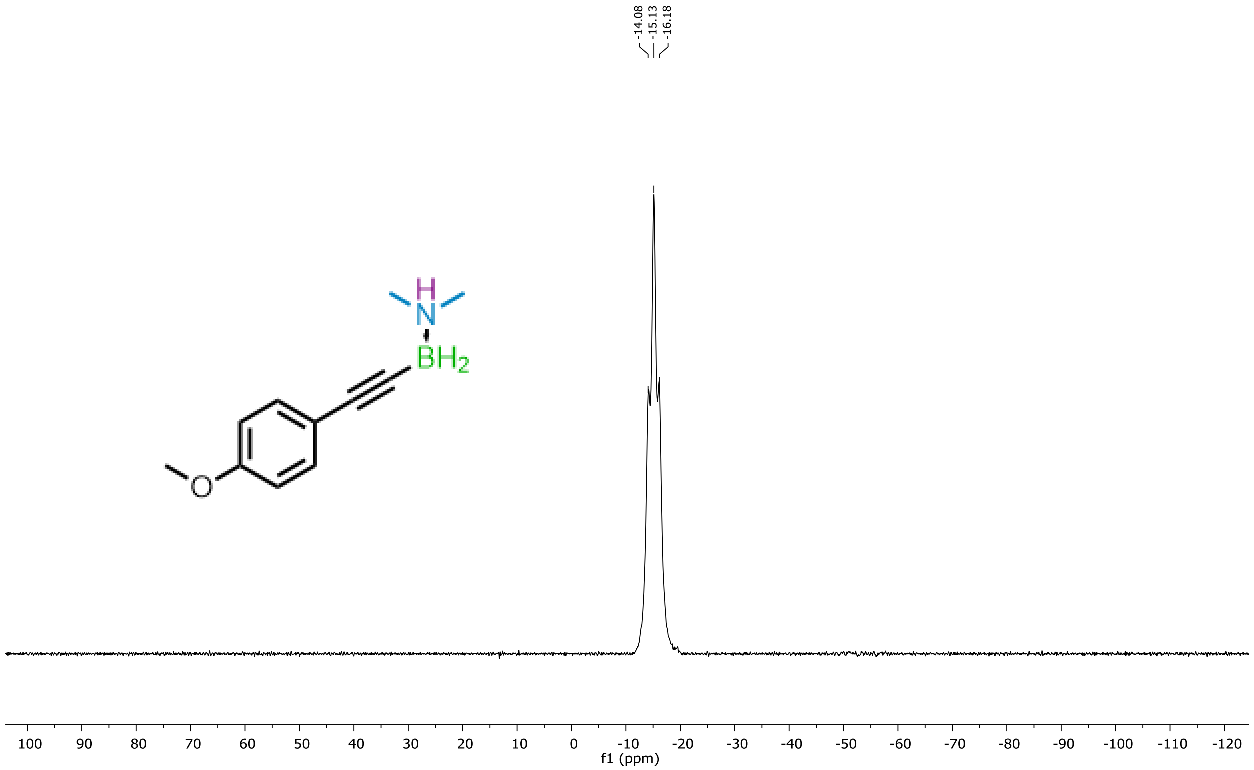

^{11}B NMR (96 MHz, $CDCl_3$) ((4-methoxyphenyl)ethynyl)borane-dimethylamine (**3be**)

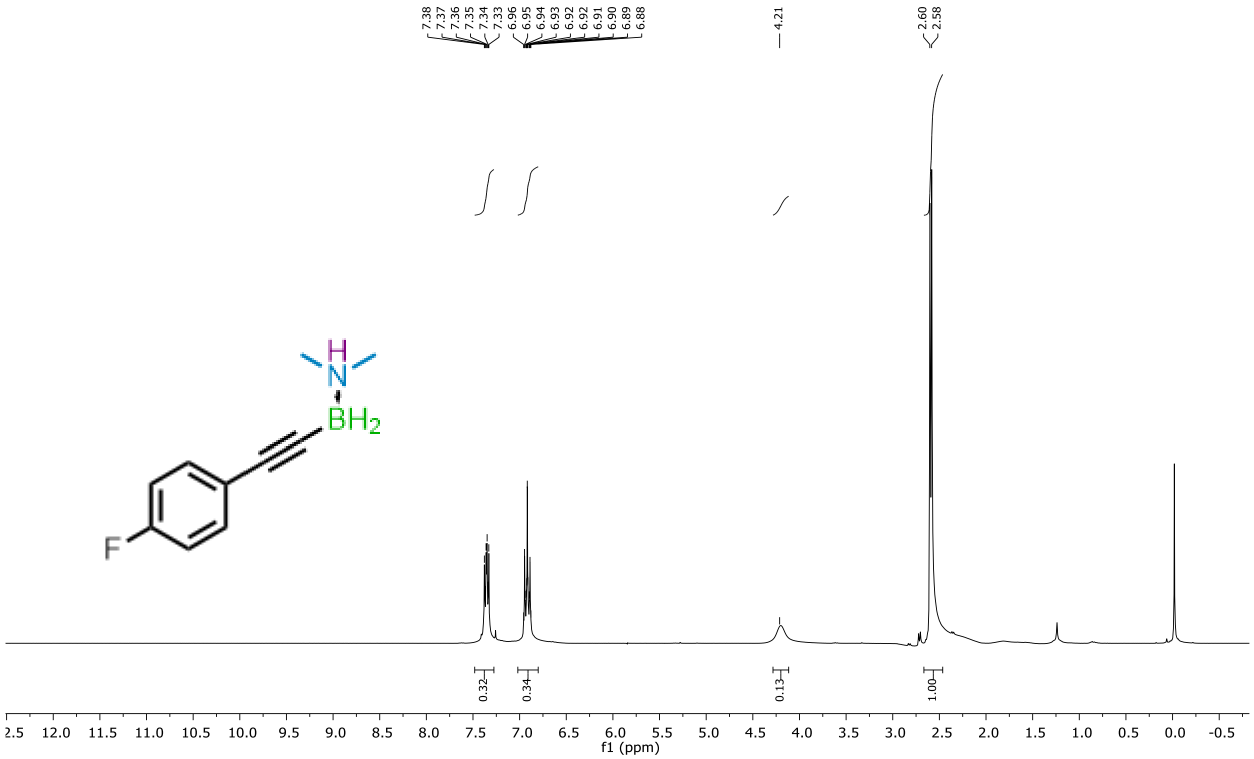

^{1}H NMR (300 MHz, $CDCl_3$) ((4-fluorophenyl)ethynyl)borane-dimethylamine (**3ce**)

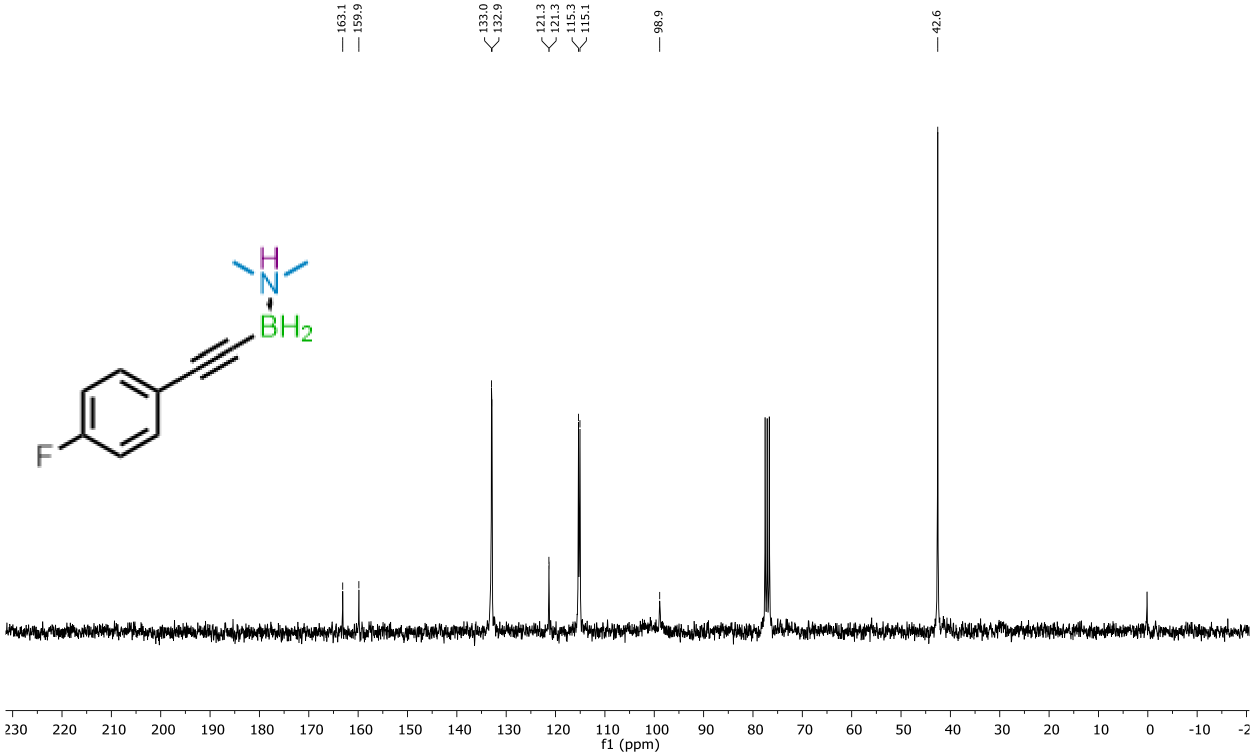

^{13}C NMR (75 MHz, $CDCl_3$) ((4-fluorophenyl)ethynyl)borane-dimethylamine (**3ce**)

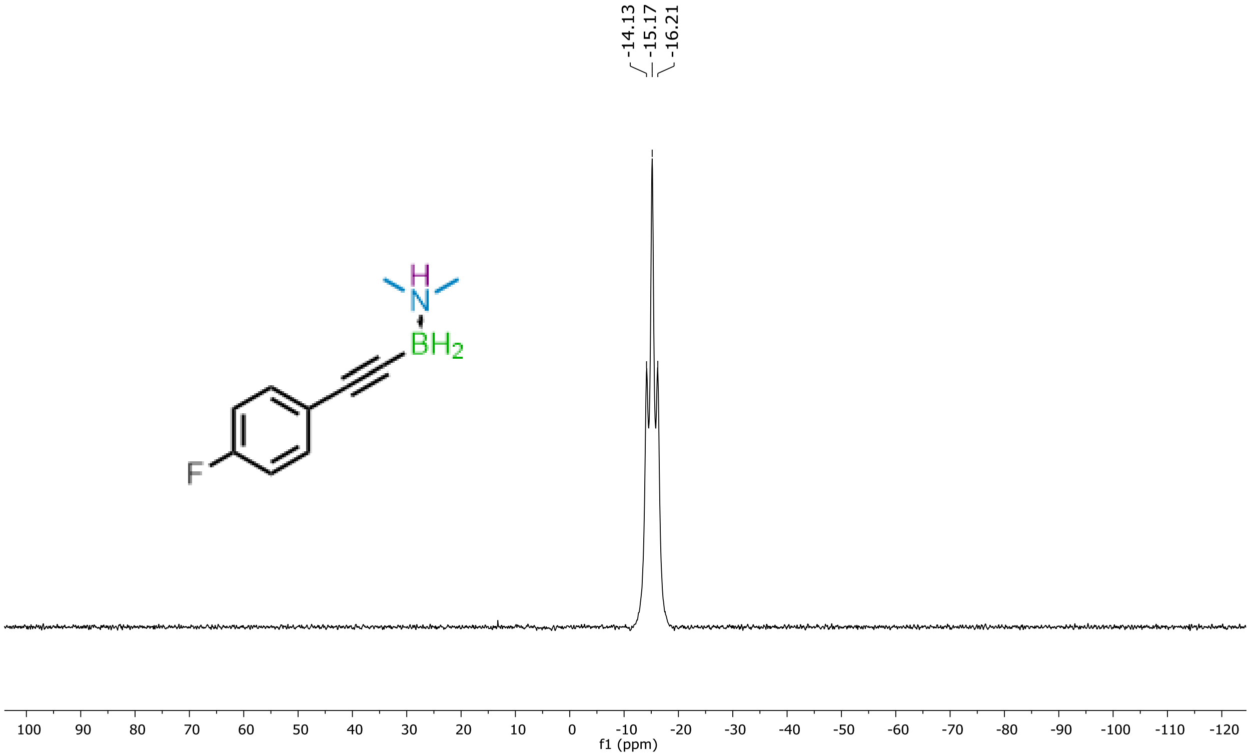

^{11}B NMR (96 MHz, $CDCl_3$) ((4-fluorophenyl)ethynyl)borane-dimethylamine (**3ce**)

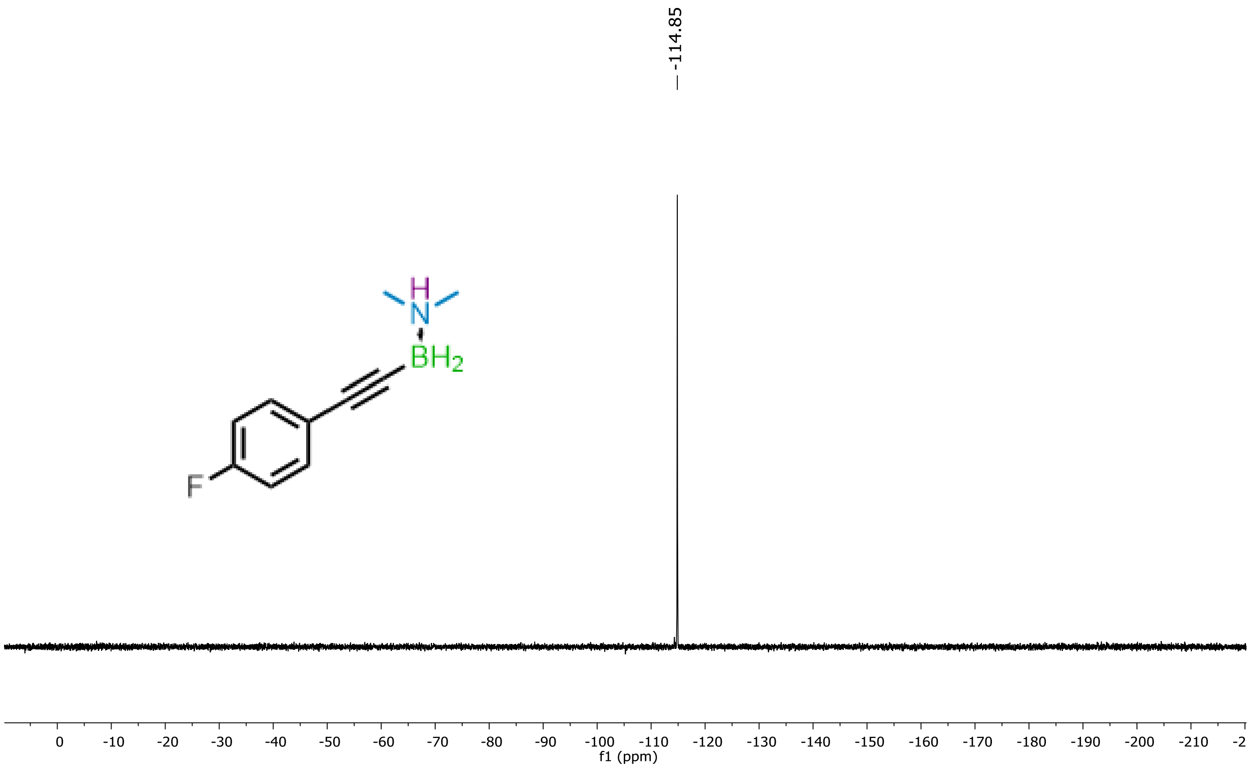

^{19}F NMR (282 MHz, $CDCl_3$) ((4-fluorophenyl)ethynyl)borane-dimethylamine (**3ce**)

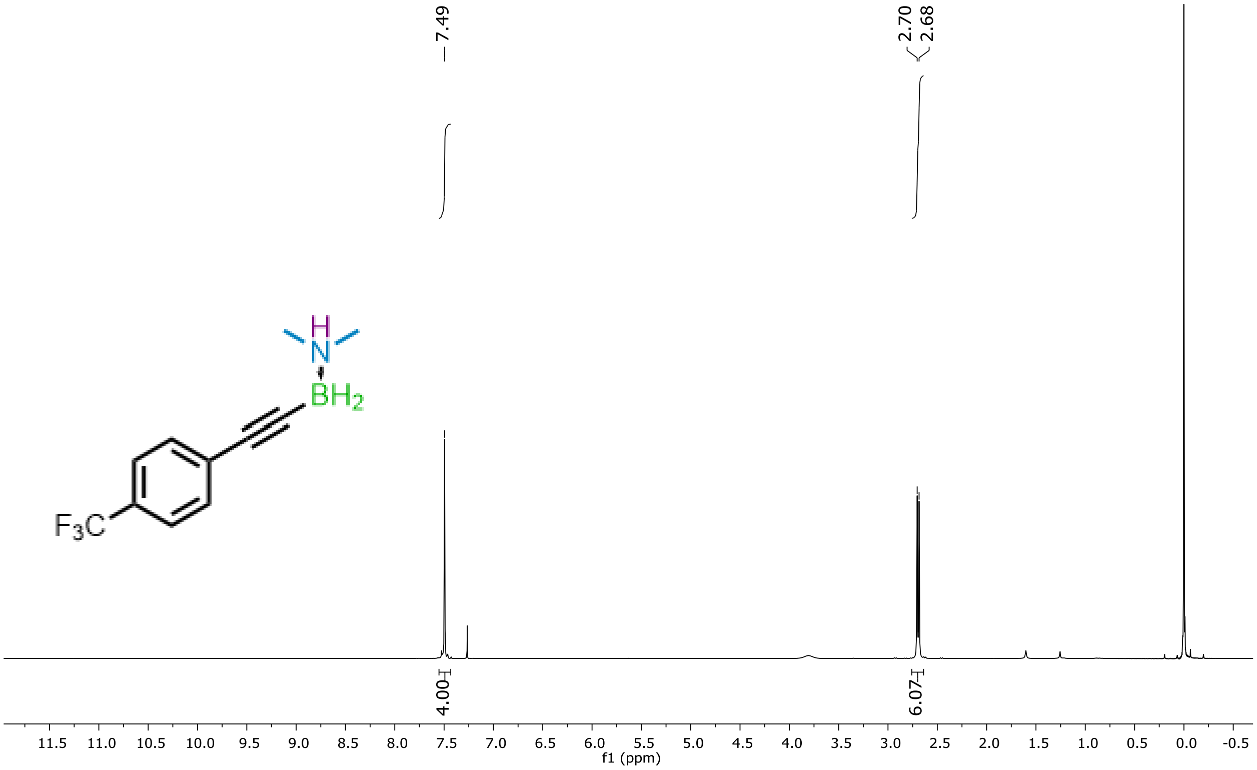

^{1}H NMR (300 MHz, $CDCl_3$) ((4-(trifluoromethyl)phenyl)ethynyl)borane-dimethylamine (**3de**)

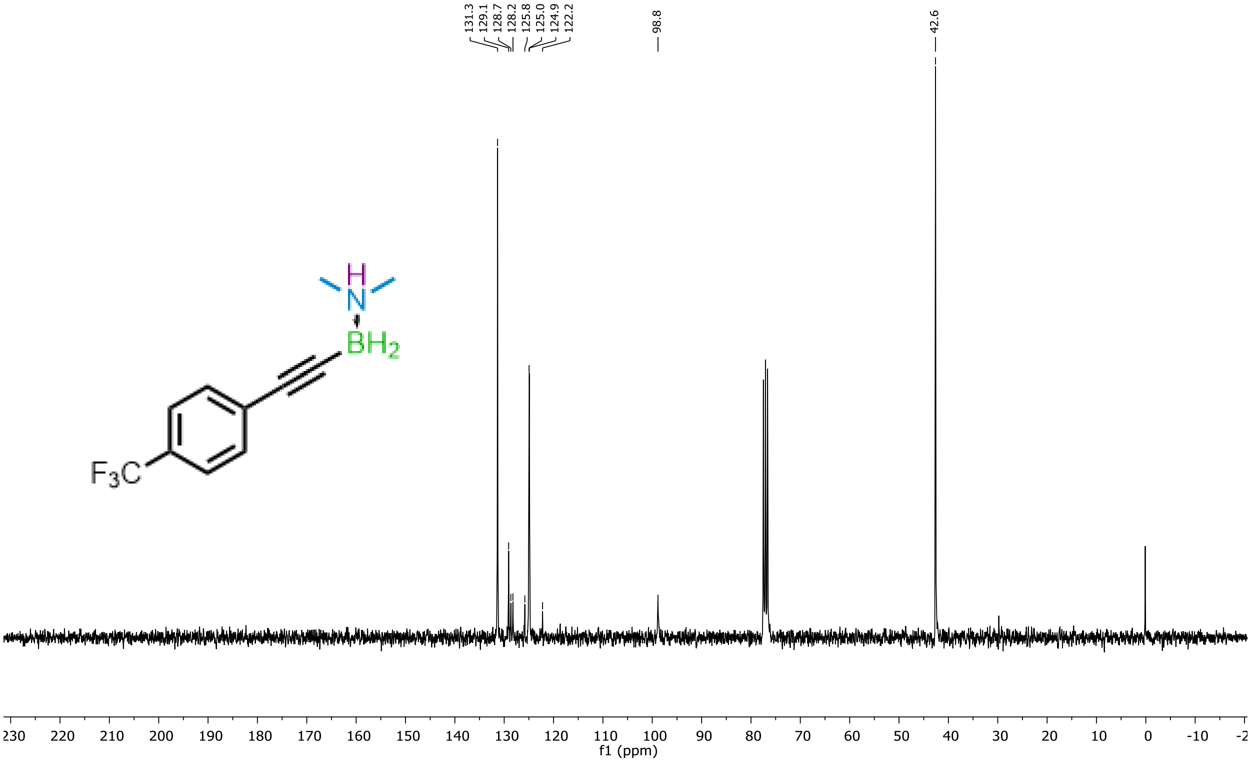

^{13}C NMR (75 MHz, $CDCl_3$) ((4-(trifluoromethyl)phenyl)ethynyl)borane-dimethylamine (**3de**)

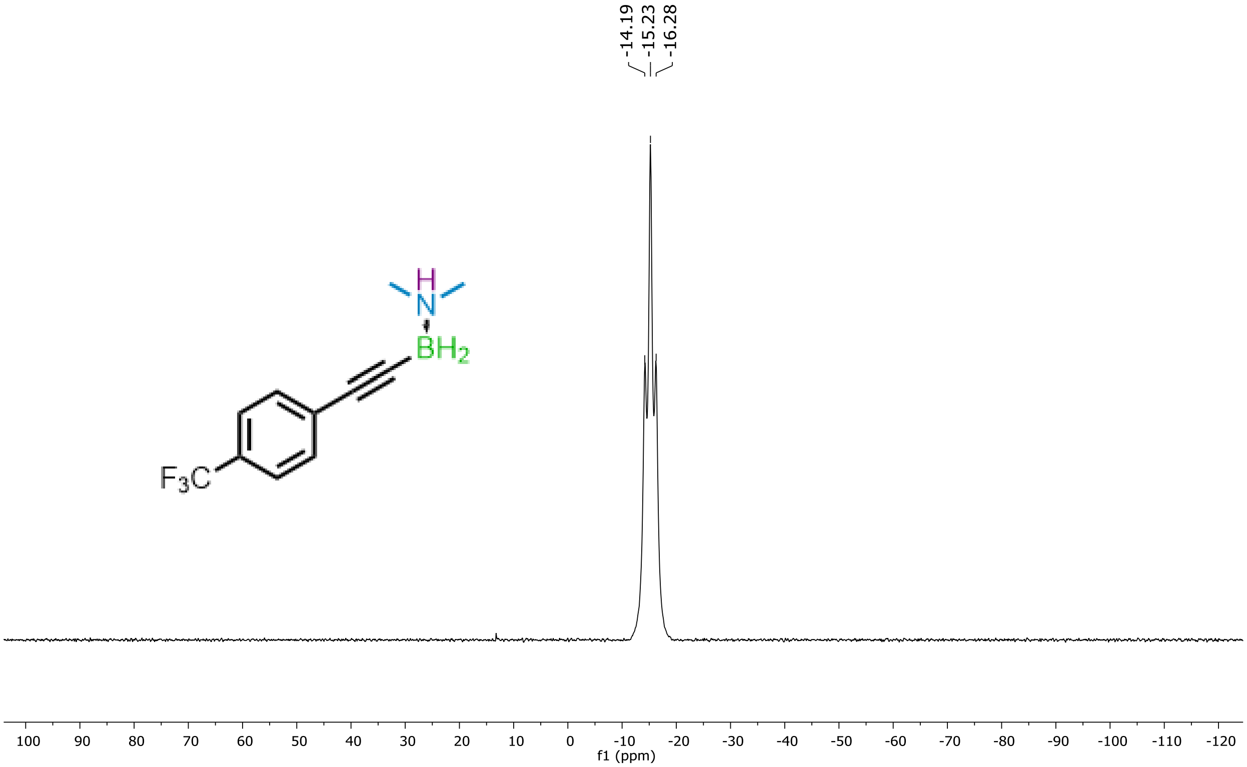

^{11}B NMR (96 MHz, $CDCl_3$) ((4-(trifluoromethyl)phenyl)ethynyl)borane-dimethylamine (**3de**)

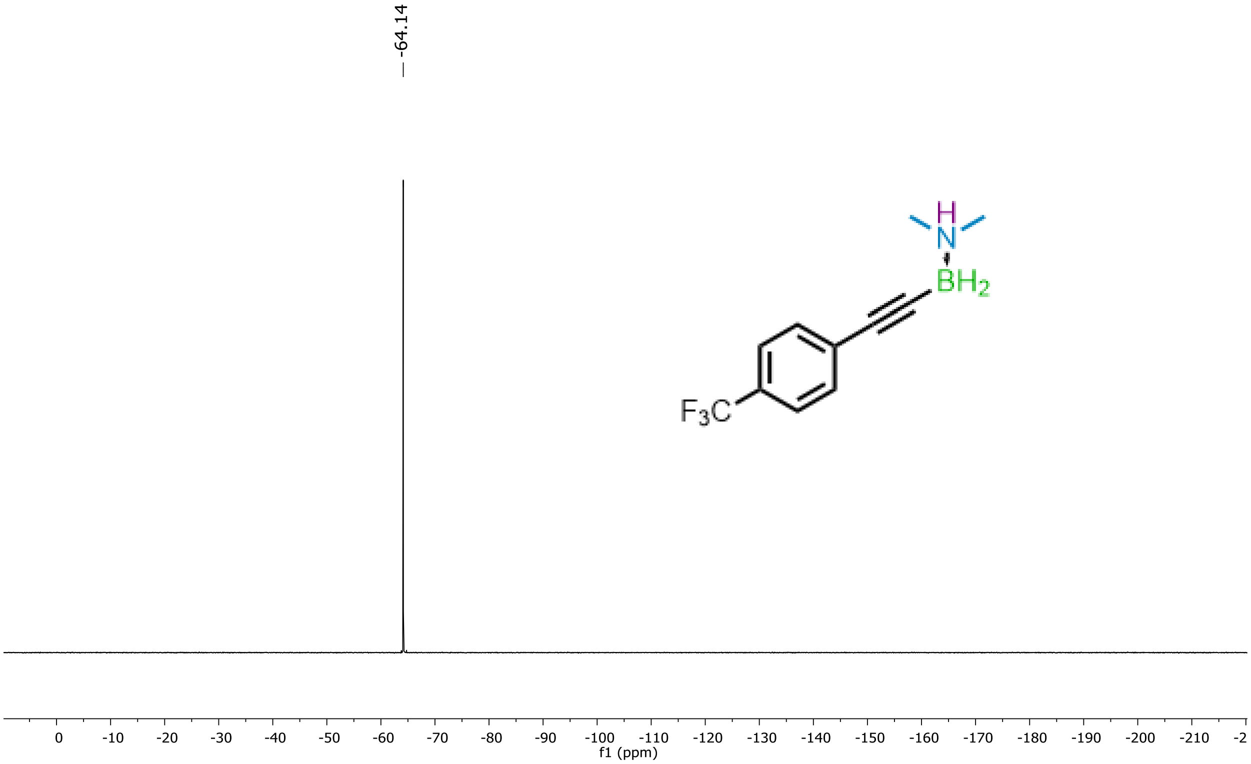

^{19}F NMR (282 MHz, $CDCl_3$) ((4-(trifluoromethyl)phenyl)ethynyl)borane-dimethylamine (**3de**)

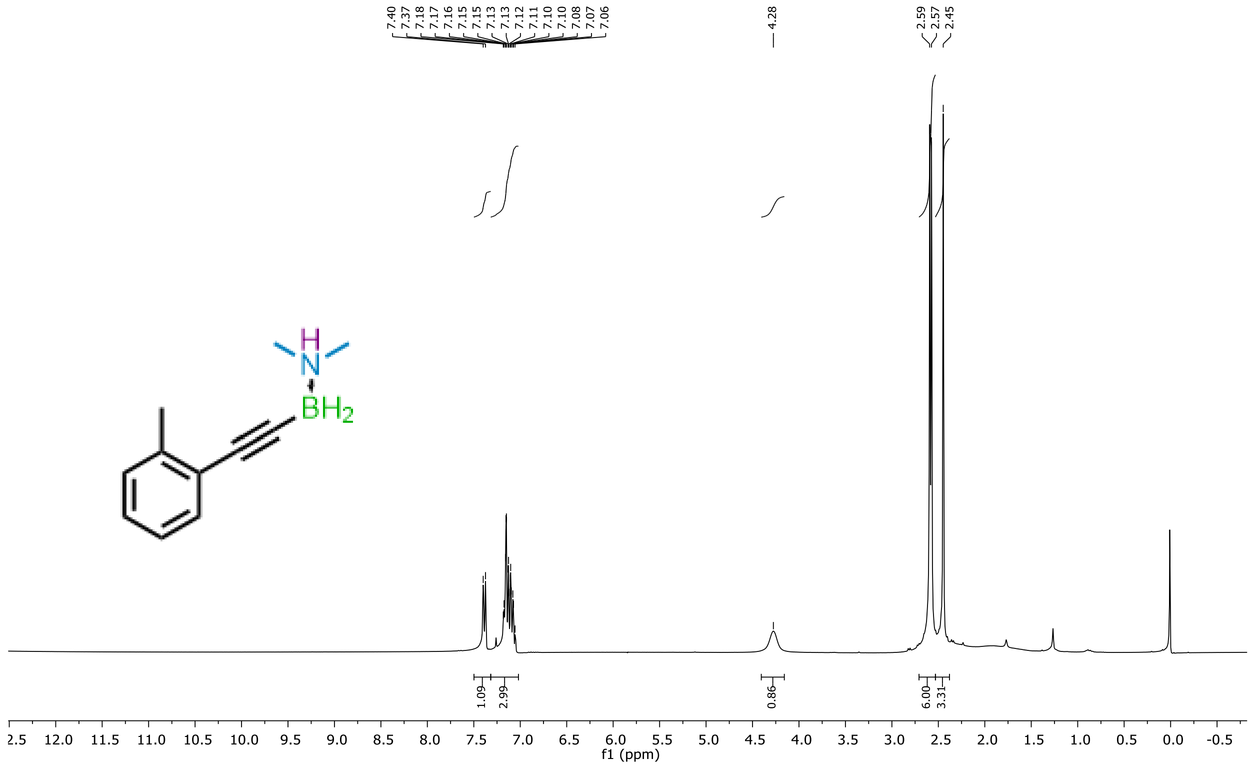

^{1}H NMR (300 MHz, $CDCl_3$) ((2-methylphenyl)ethynyl)borane-dimethylamine (**3ee**)

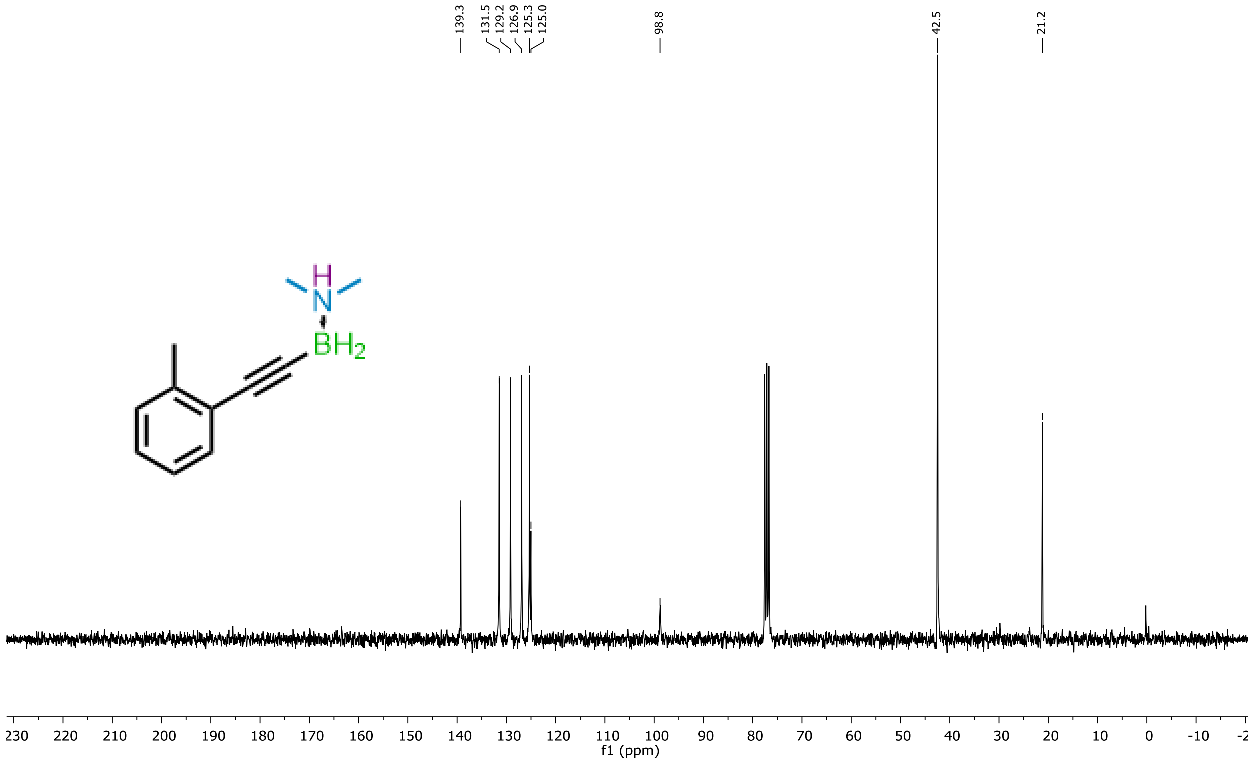

^{13}C NMR (75 MHz, $CDCl_3$) ((2-methylphenyl)ethynyl)borane-dimethylamine (**3ee**)

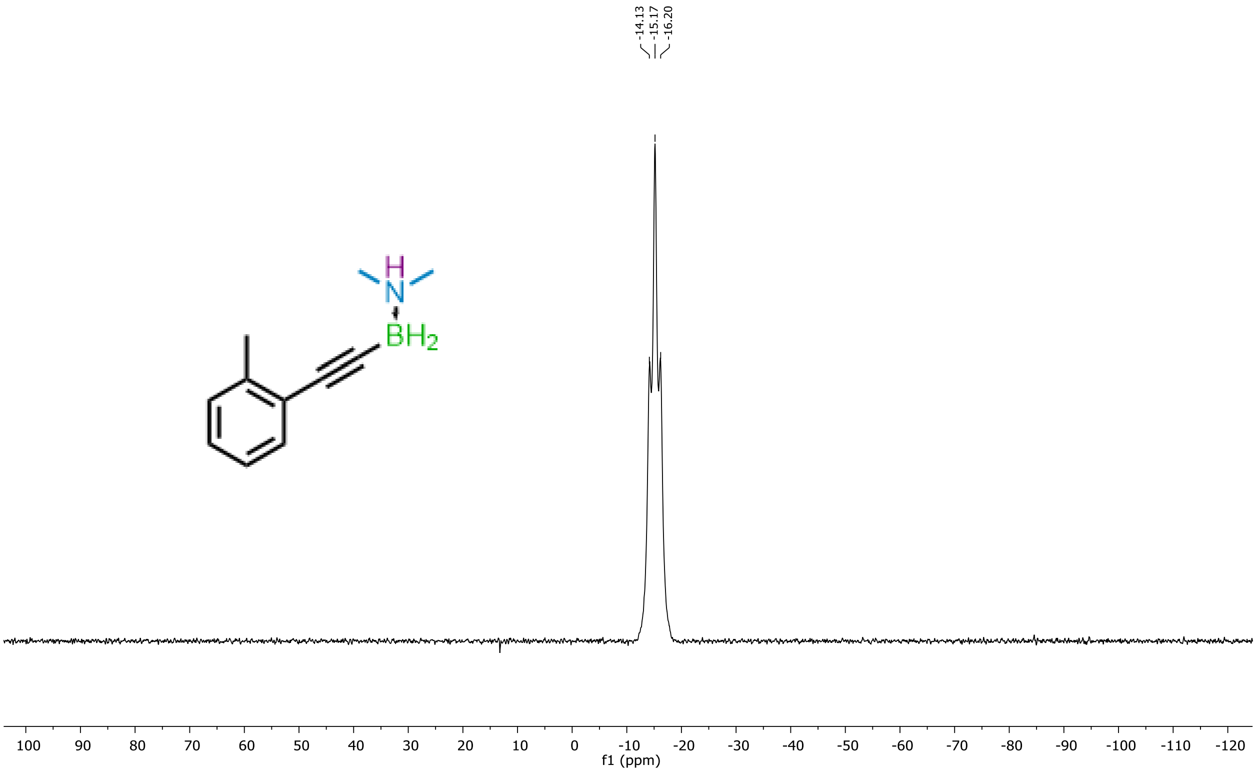

^{11}B NMR (96 MHz, $CDCl_3$) ((2-methylphenyl)ethynyl)borane-dimethylamine (**3ee**)

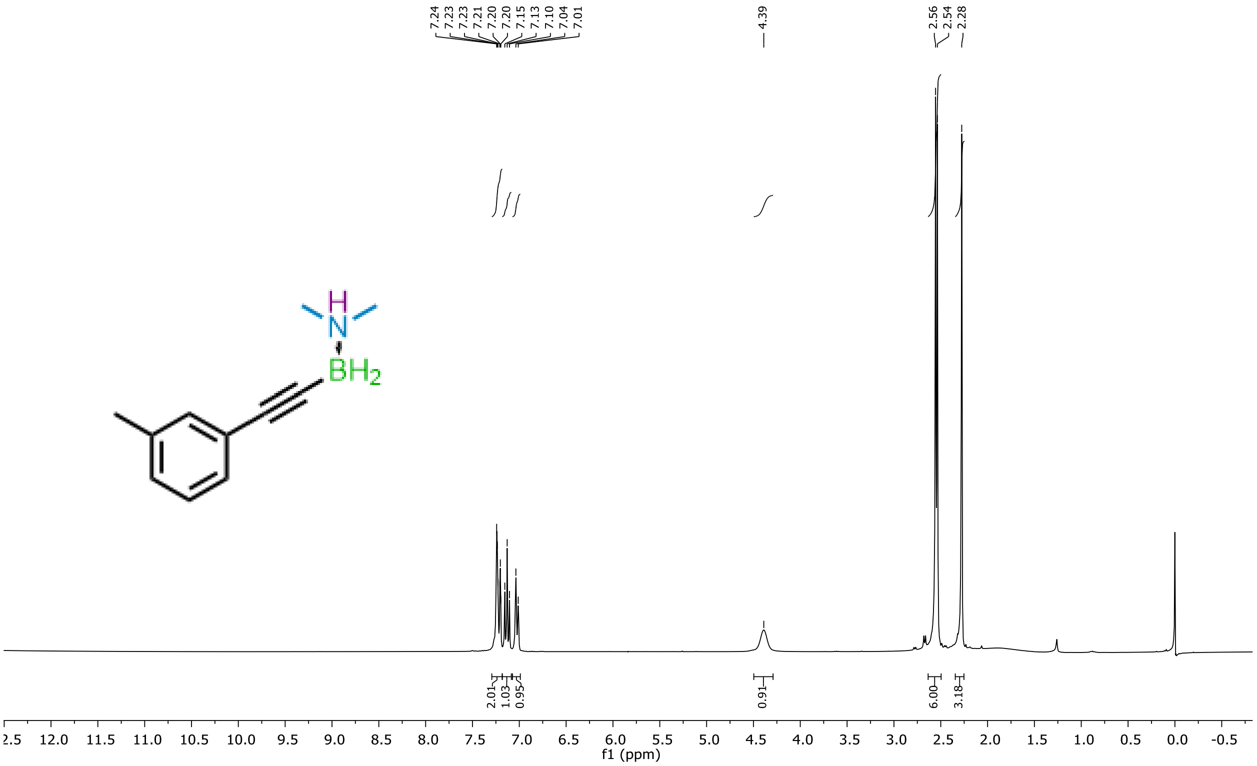

^{1}H NMR (300 MHz, $CDCl_3$) ((3-methylphenyl)ethynyl)borane-dimethylamine (**3fe**)

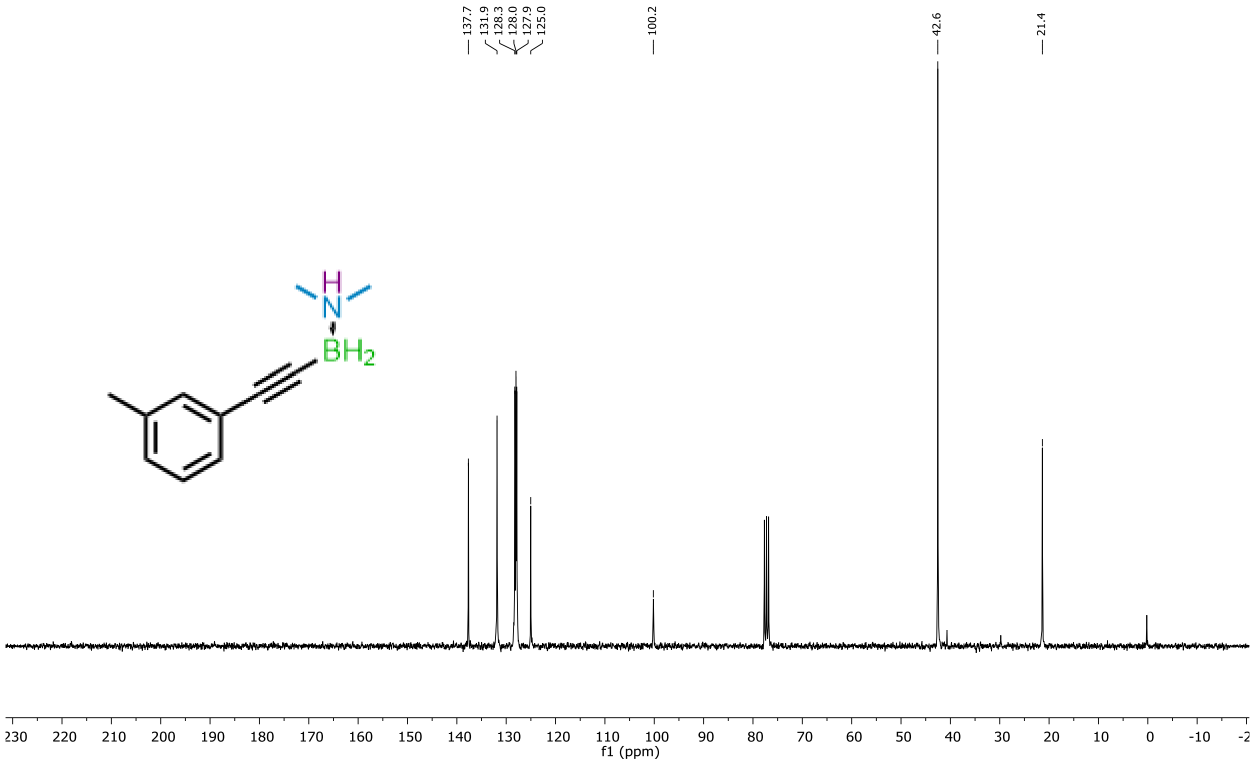

^{13}C NMR (75 MHz, $CDCl_3$) ((3-methylphenyl)ethynyl)borane-dimethylamine (**3fe**)

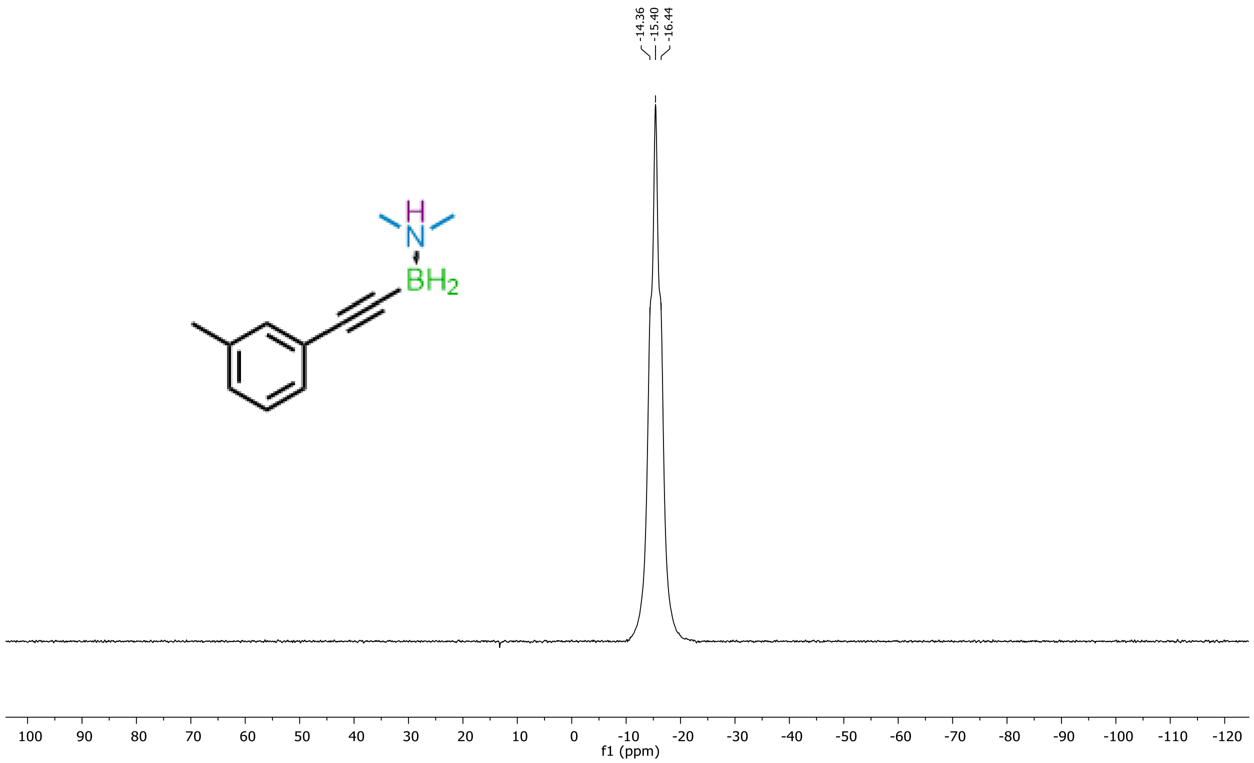

^{11}B NMR (96 MHz, $CDCl_3$) ((3-methylphenyl)ethynyl)borane-dimethylamine (**3fe**)

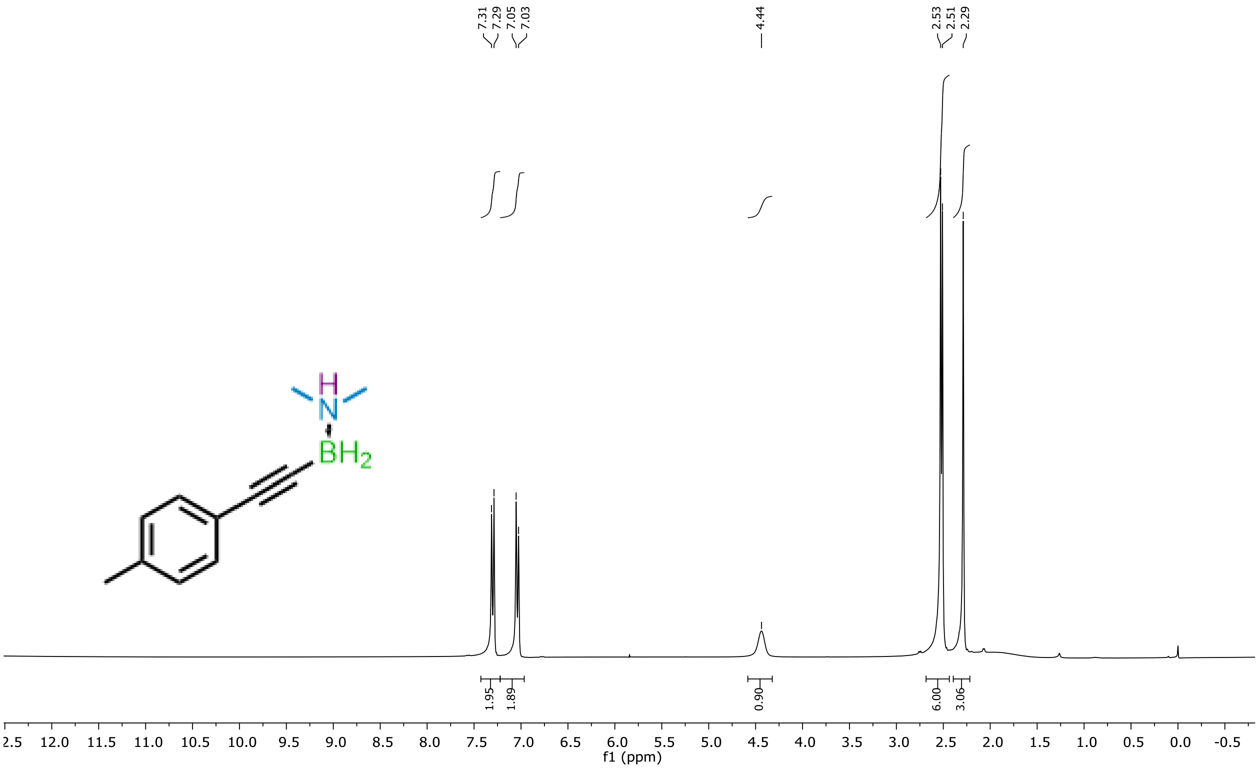

^{1}H NMR (300 MHz, $CDCl_3$) ((4-methylphenyl)ethyny)borane-dimethylamine (**3ge**)

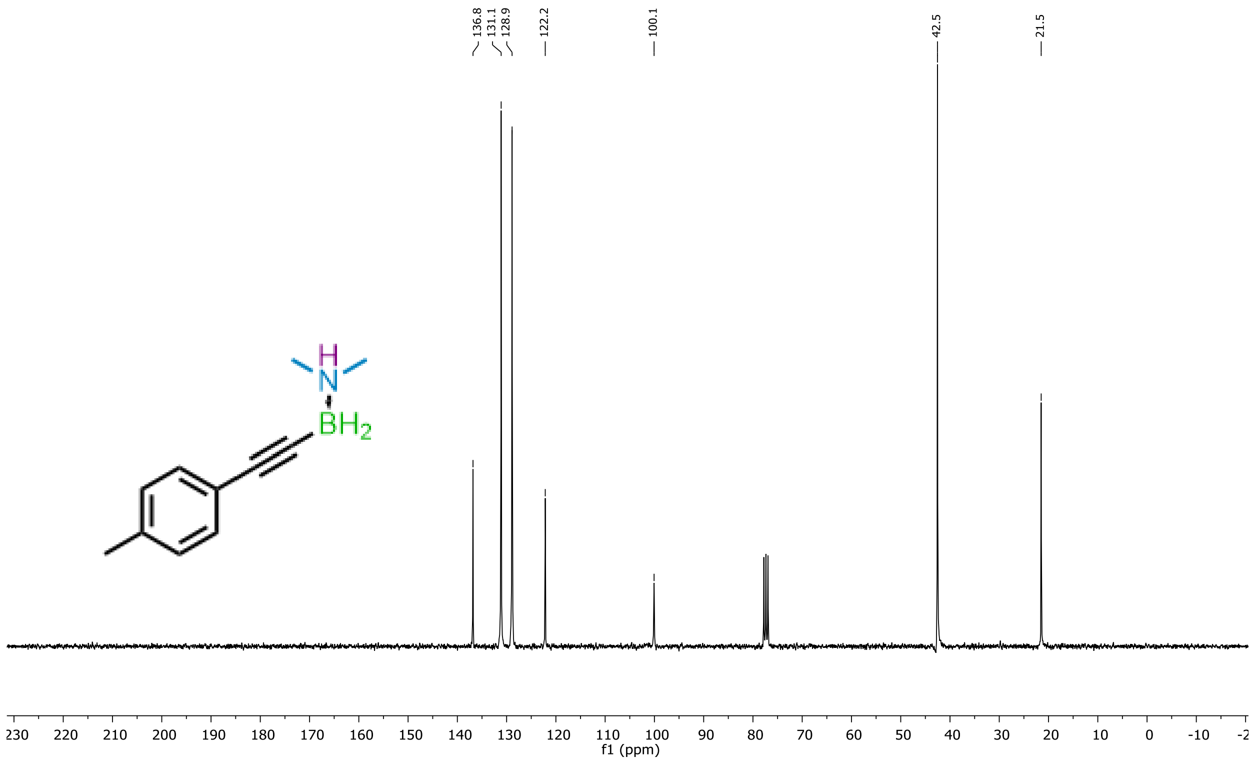

^{13}C NMR (75 MHz, $CDCl_3$) ((4-methylphenyl)ethyny)borane-dimethylamine (**3ge**)

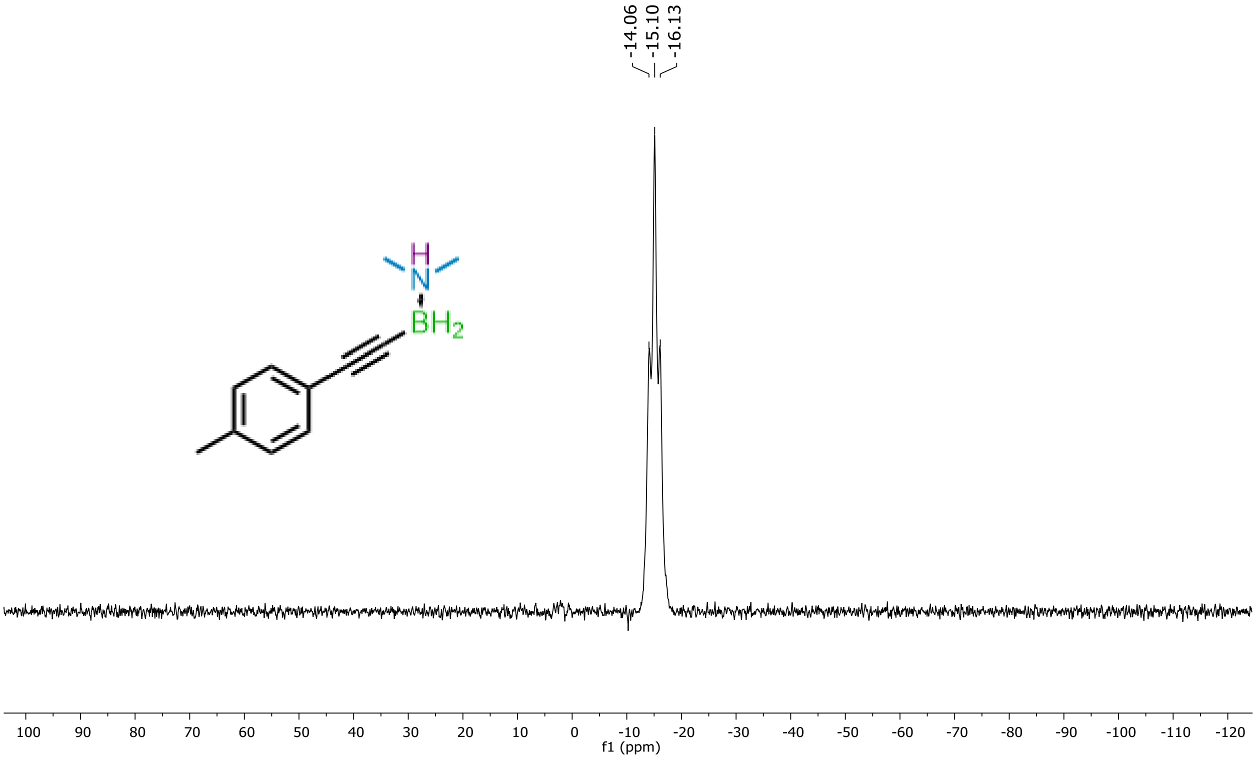

^{11}B NMR (96 MHz, $CDCl_3$) ((4-methylphenyl)ethyny)borane-dimethylamine (**3ge**)

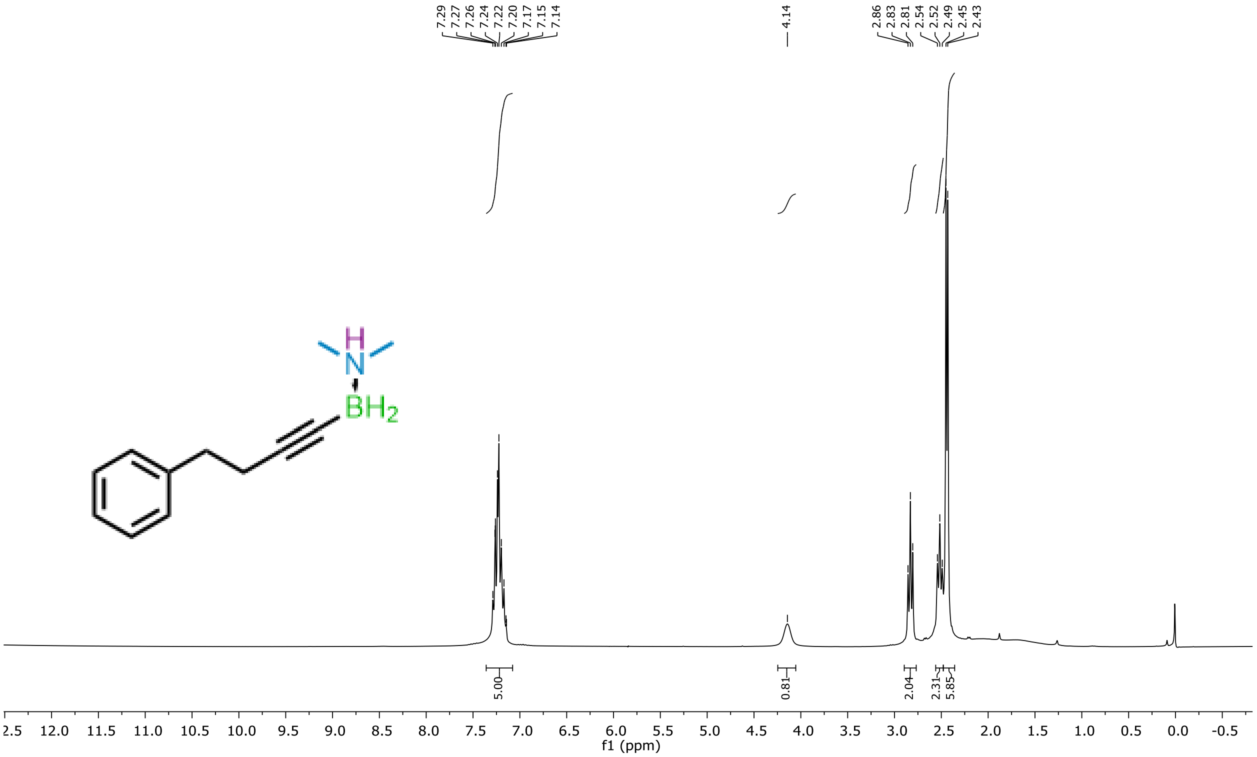

^{1}H NMR (300 MHz, $CDCl_3$) (4-phenylbut-1-yn-1-yl)borane-dimethylamine (**3he**)

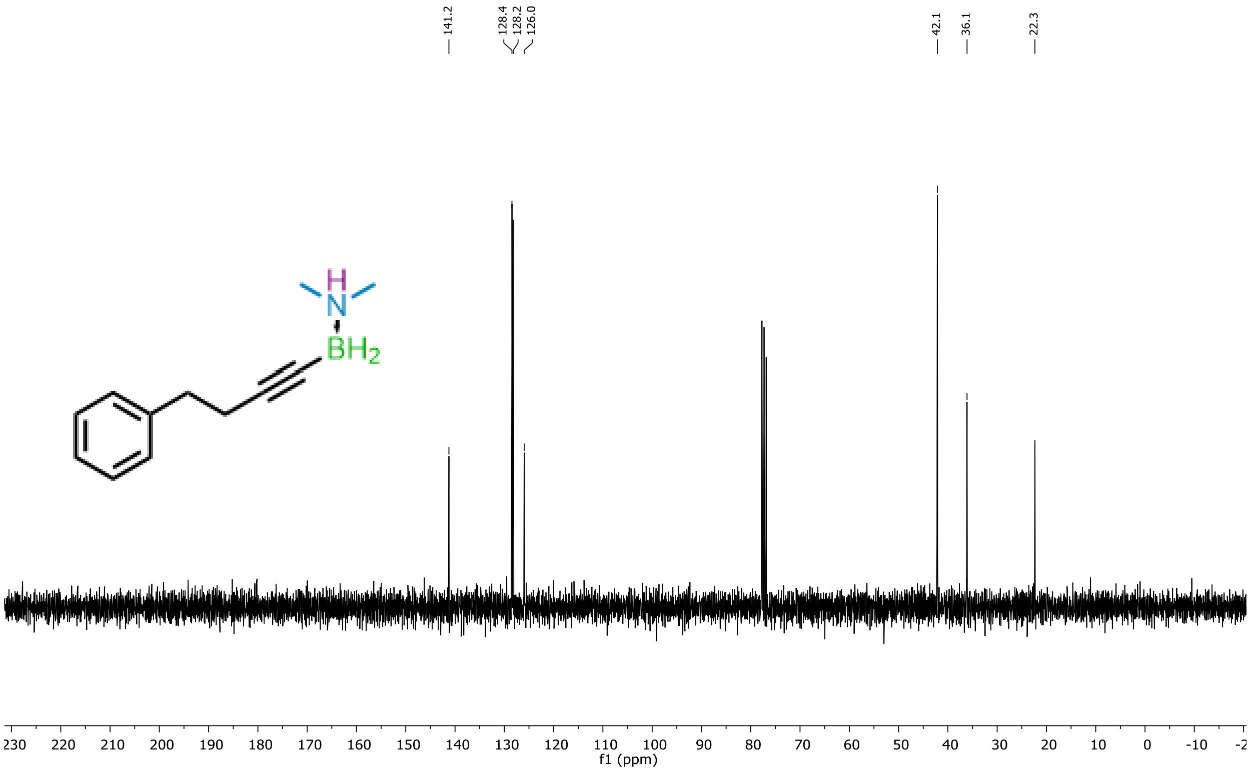

^{13}C NMR (75 MHz, $CDCl_3$) (4-phenylbut-1-yn-1-yl)borane-dimethylamine (**3he**)

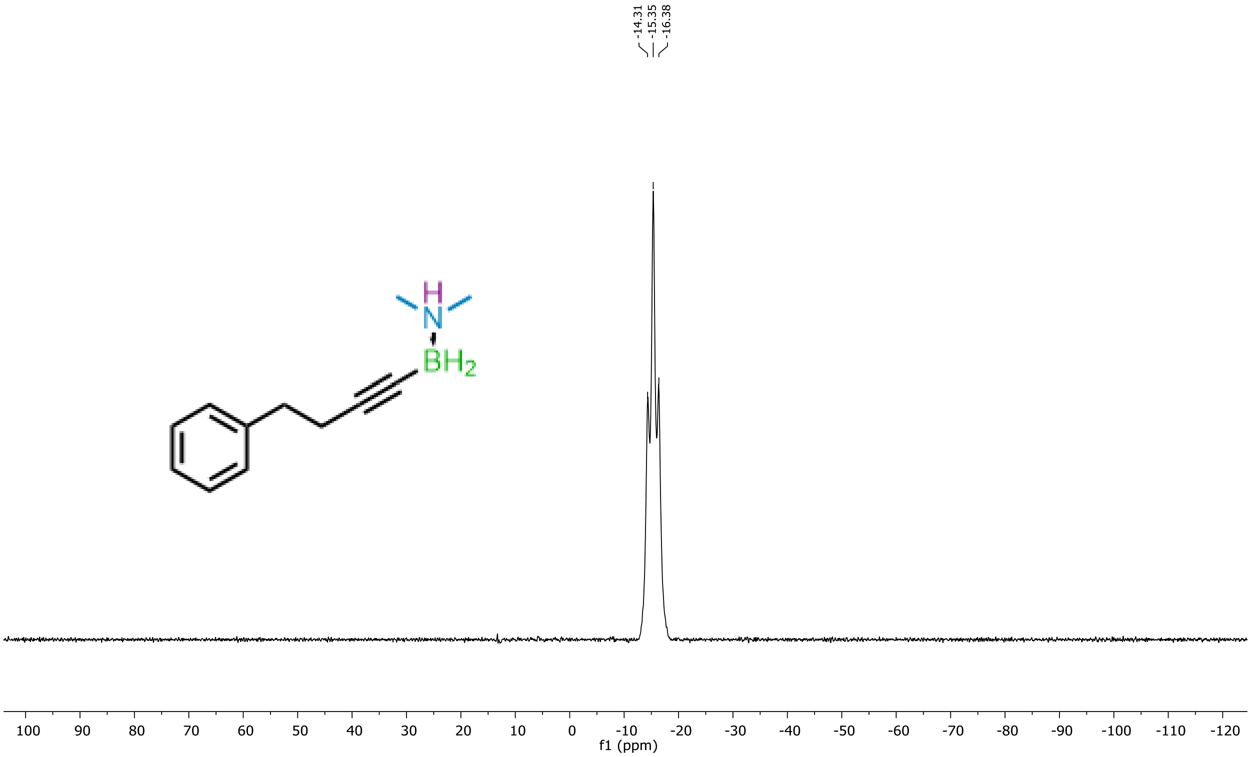

^{11}B NMR (96 MHz, $CDCl_3$) (4-phenylbut-1-yn-1-yl)borane-dimethylamine (**3he**)

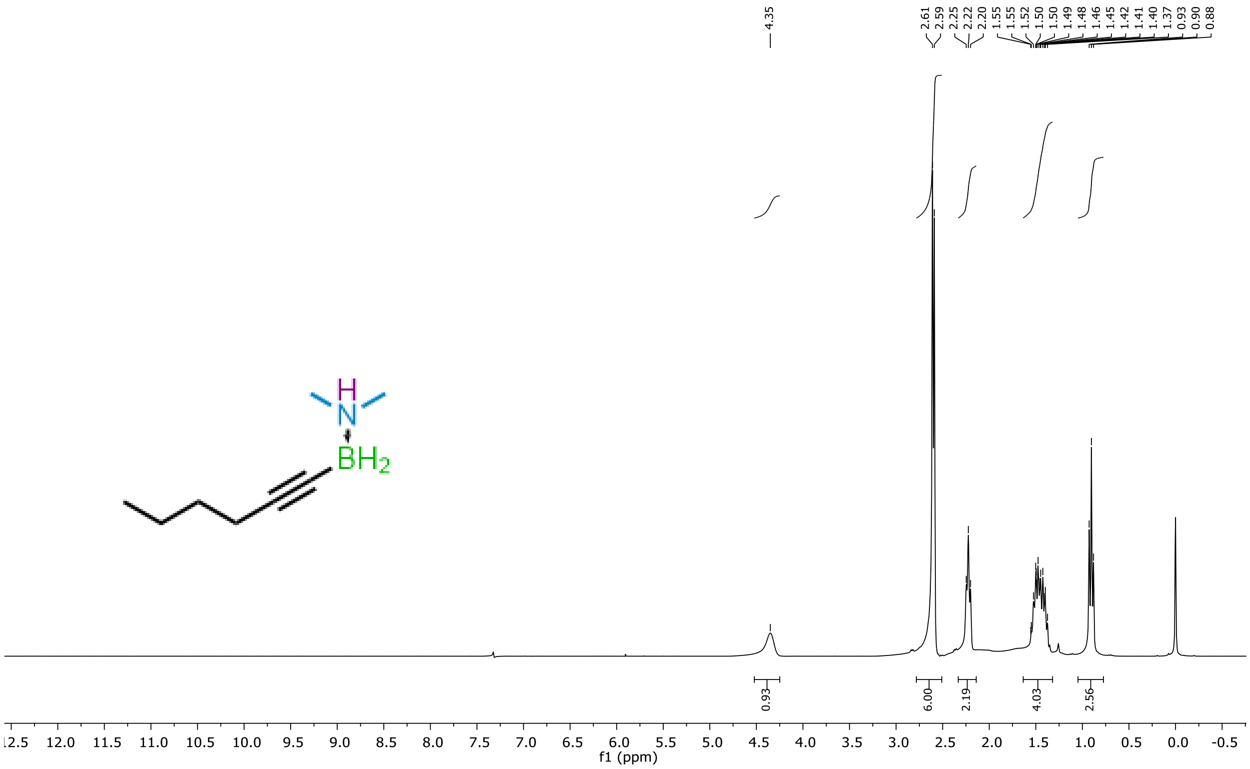

^{1}H NMR (300 MHz, $CDCl_3$) (hexynyl)borane-dimethylamine (**3ie**)

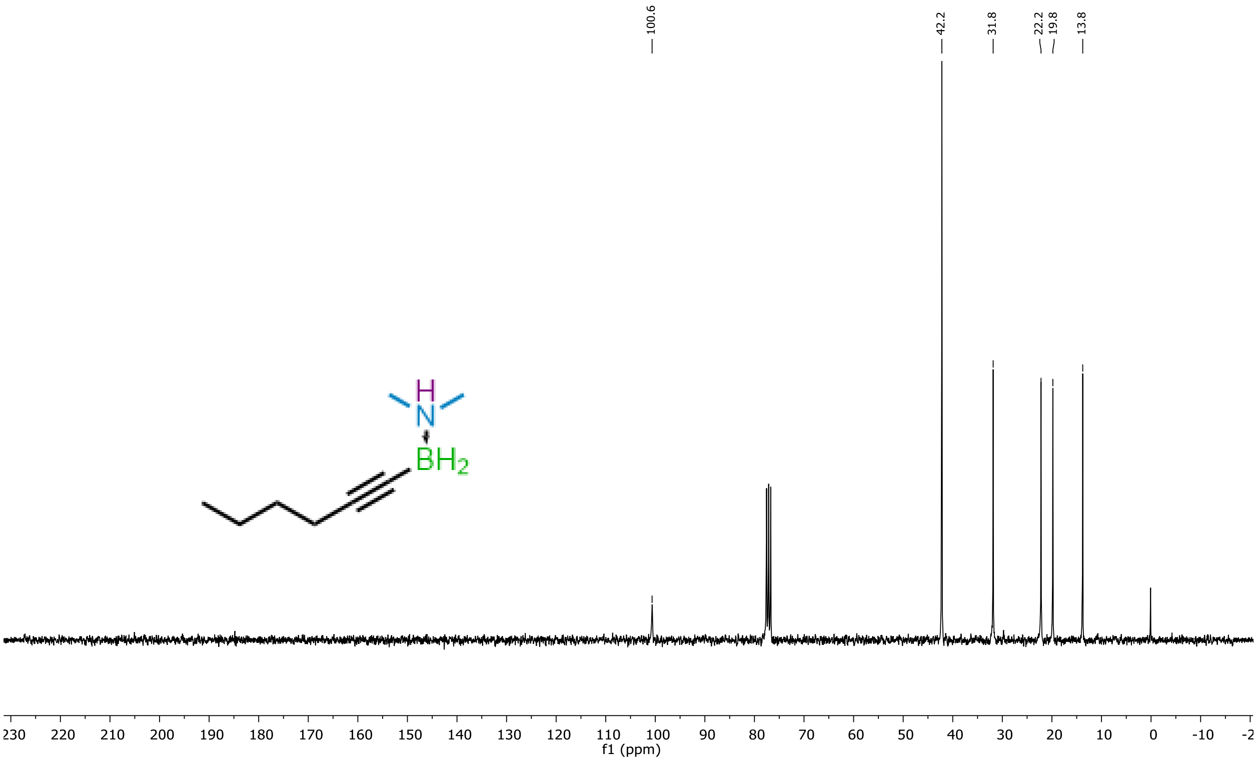

^{13}C NMR (75 MHz, $CDCl_3$) (hexynyl)borane-dimethylamine (**3ie**)

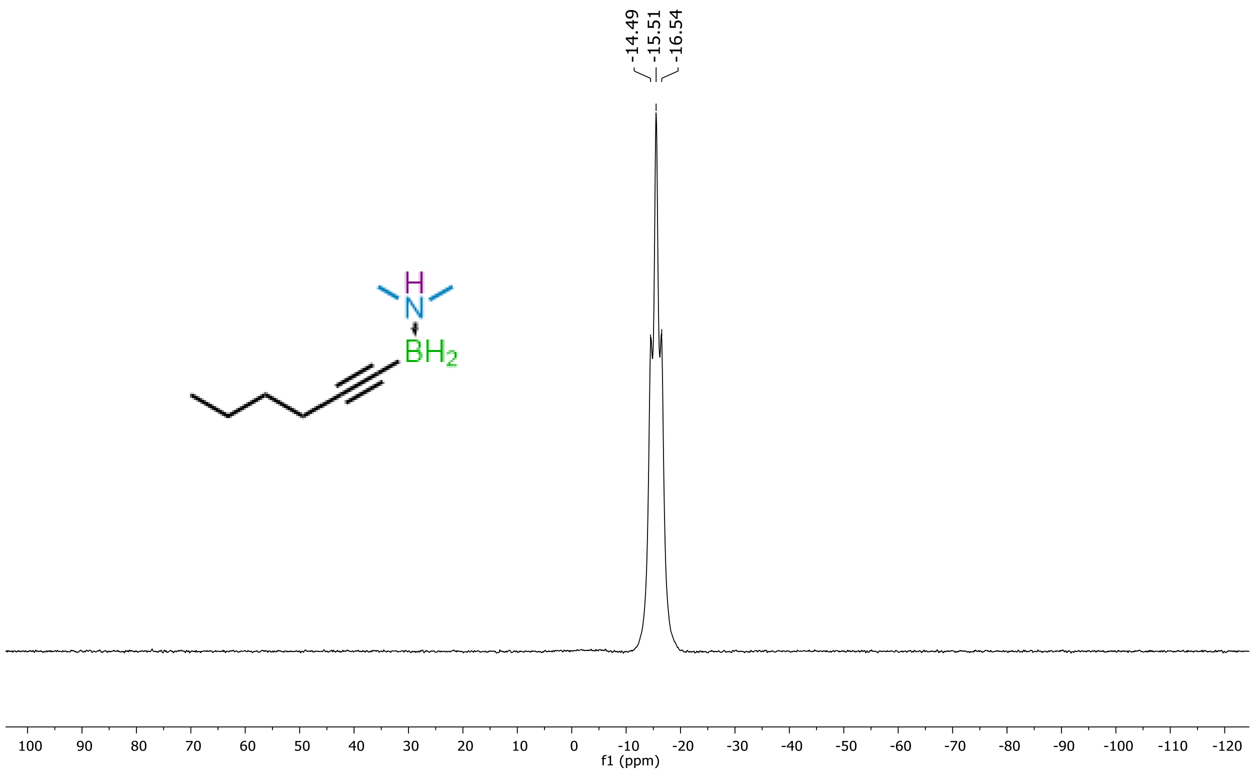

^{11}B NMR (96 MHz, $CDCl_3$) (hexynyl)borane-dimethylamine (**3ie**)

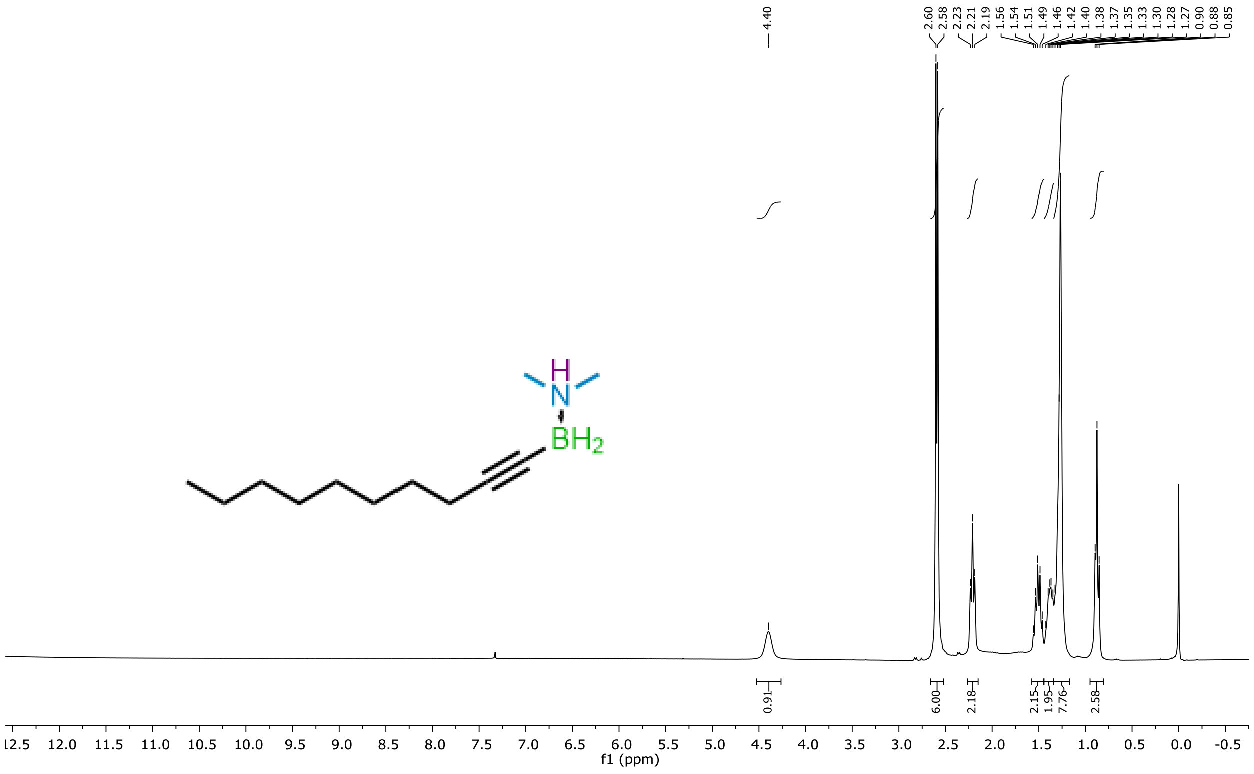

^{1}H NMR (300 MHz, $CDCl_3$) (decynyl)borane-dimethylamine (**3je**)

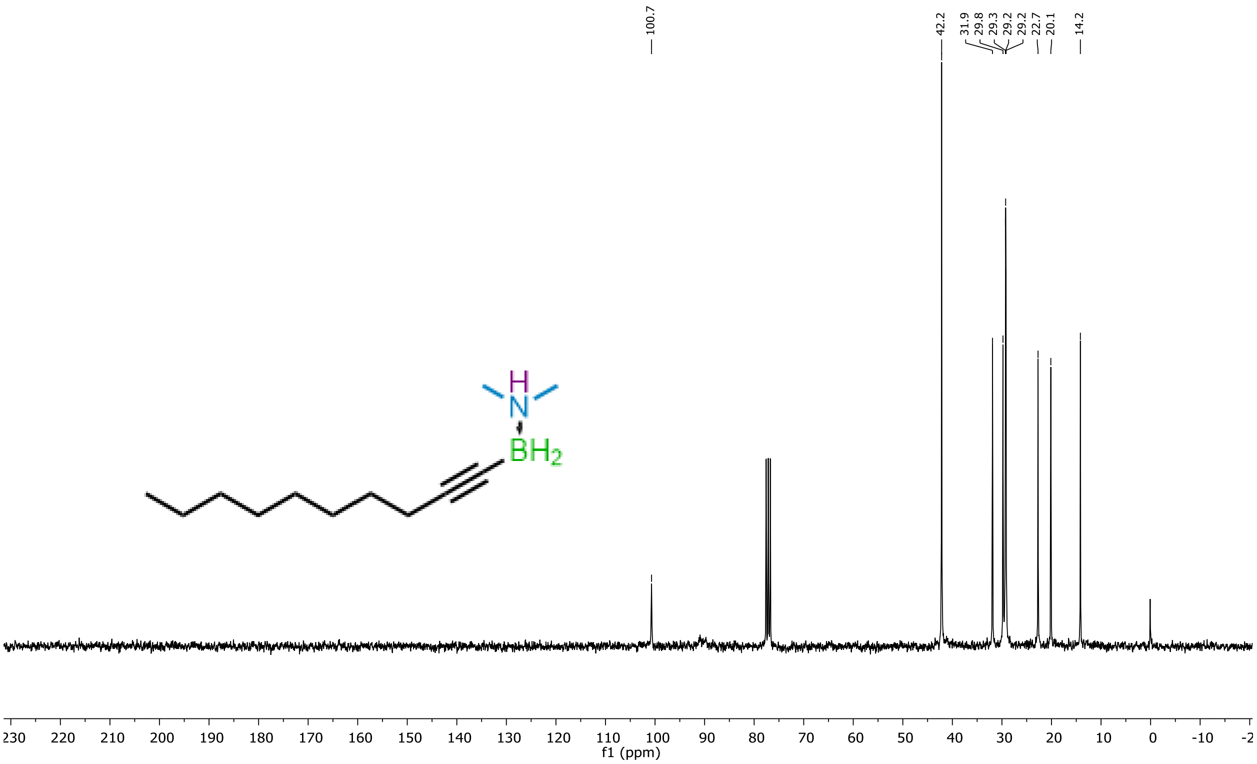

^{13}C NMR (75 MHz, $CDCl_3$) (decynyl)borane-dimethylamine (**3je**)

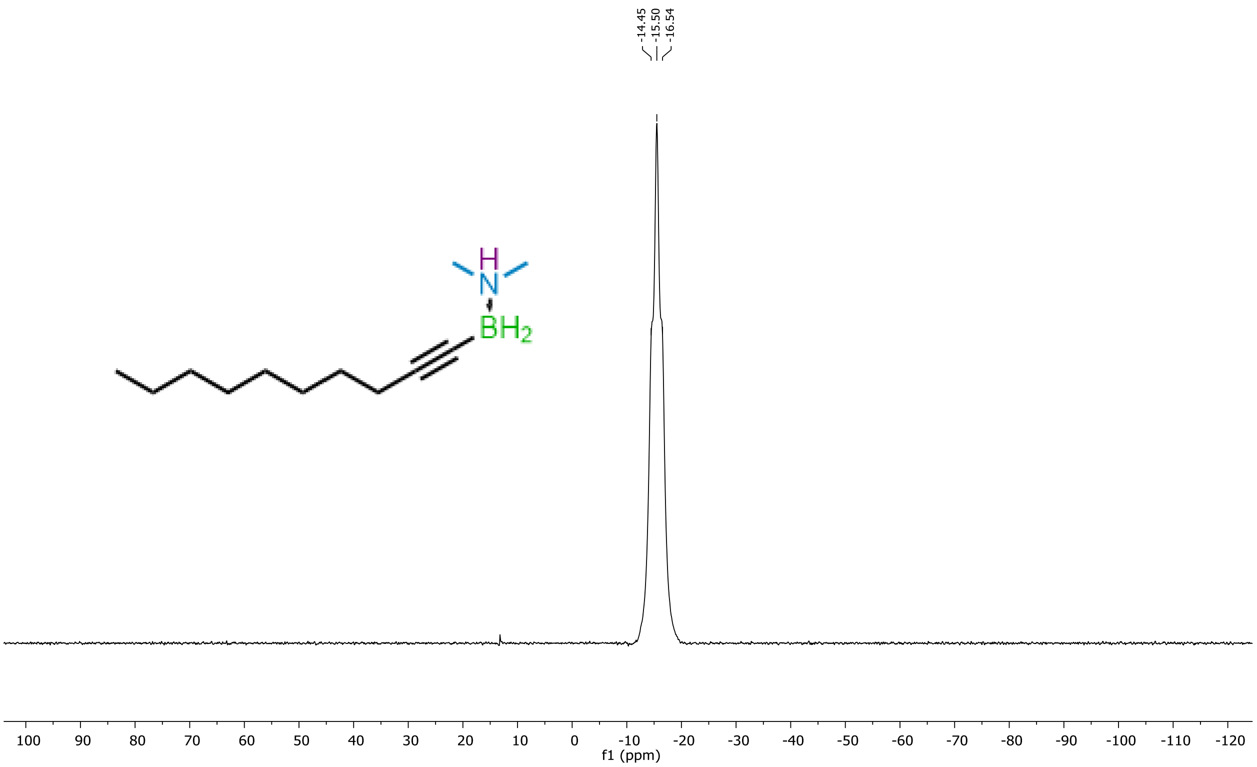

^{11}B NMR (96 MHz, $CDCl_3$) (decynyl)borane-dimethylamine (**3je**)

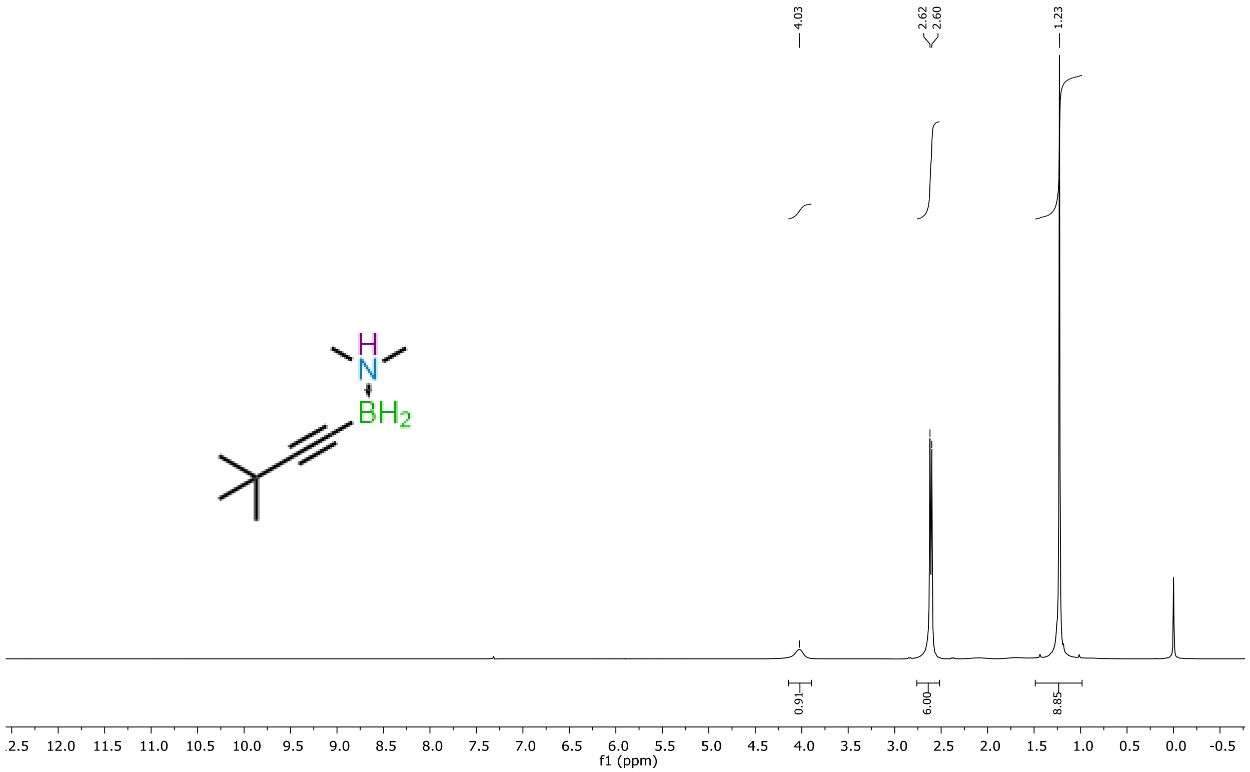

^{1}H NMR (300 MHz, $CDCl_3$) (3,3-dimethylbut-1-yn-1-yl)borane-dimethylamine (**3ke**)

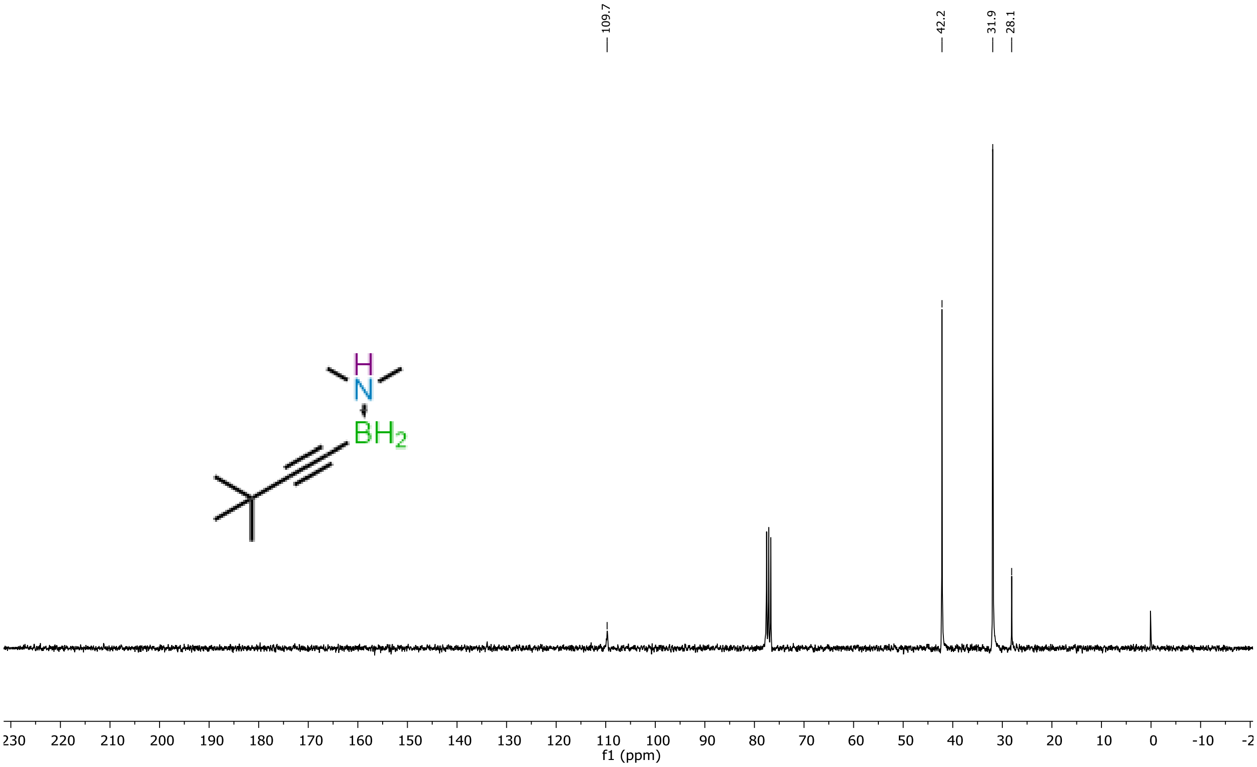

^{13}C NMR (75 MHz, $CDCl_3$) (3,3-dimethylbut-1-yn-1-yl)borane-dimethylamine (**3ke**)

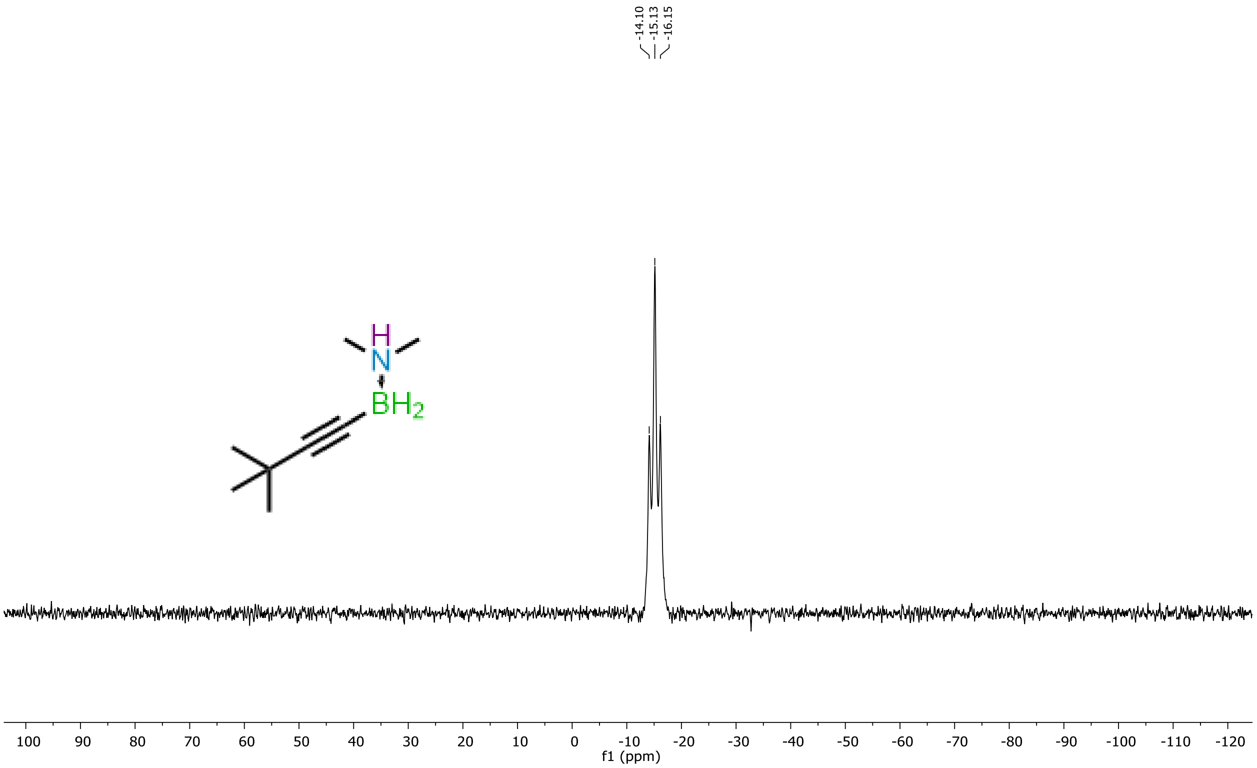

^{11}B NMR (96 MHz, $CDCl_3$) (3,3-dimethylbut-1-yn-1-yl)borane-dimethylamine (**3ke**)

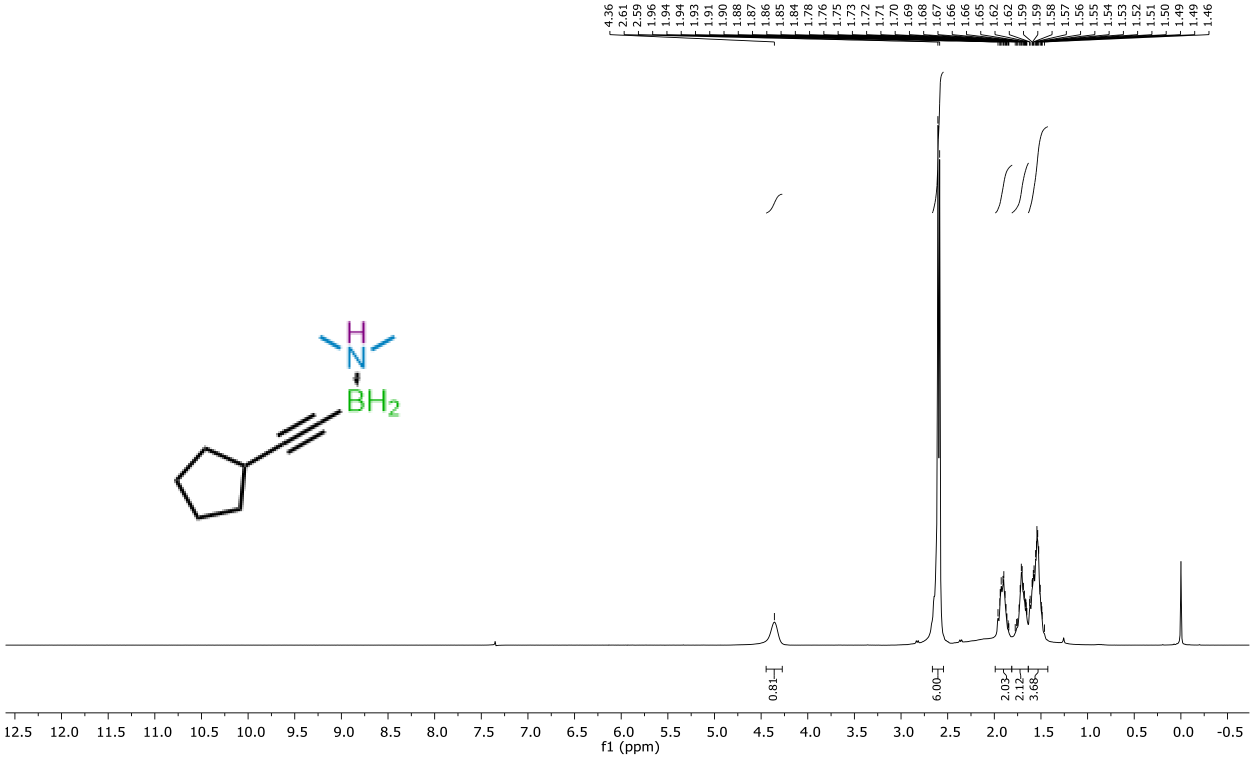

^{1}H NMR (300 MHz, $CDCl_3$) ((cyclopentyl)ethynyl)borane-dimethylamine (**3le**)

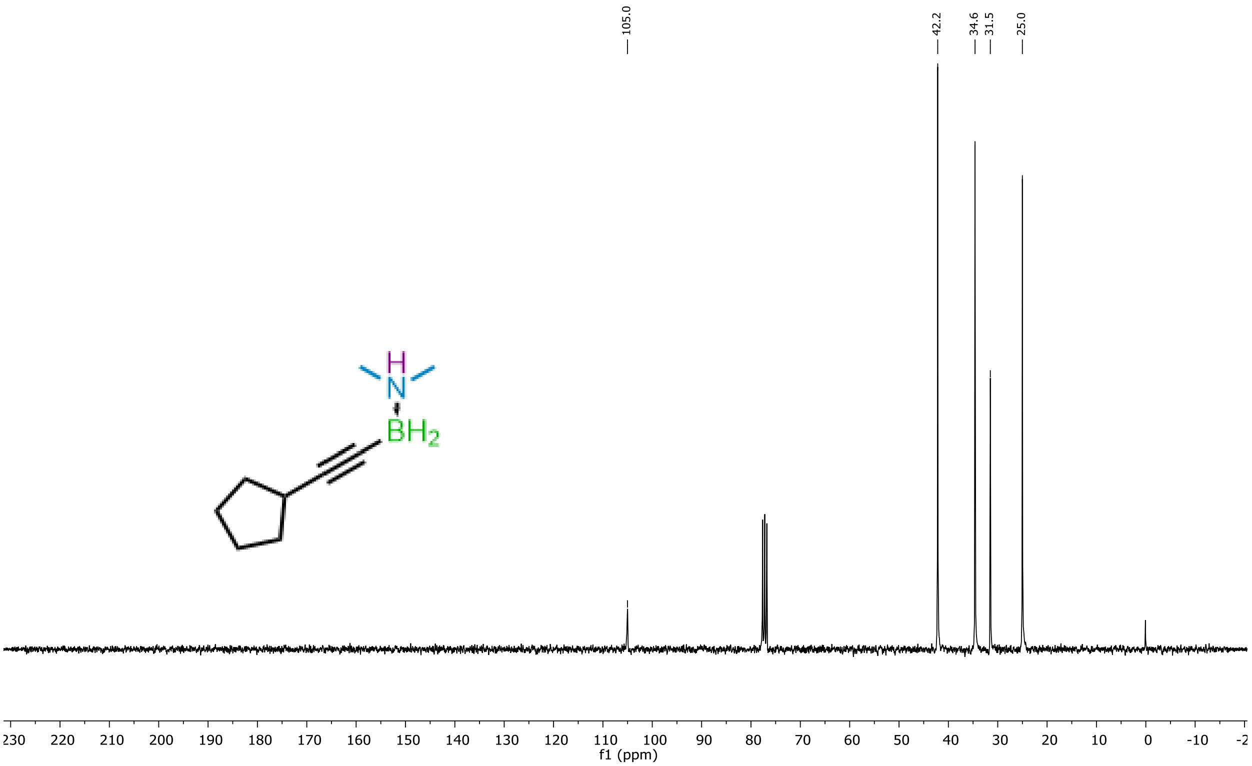

^{13}C NMR (75 MHz, $CDCl_3$) ((cyclopentyl)ethynyl)borane-dimethylamine (**3le**)

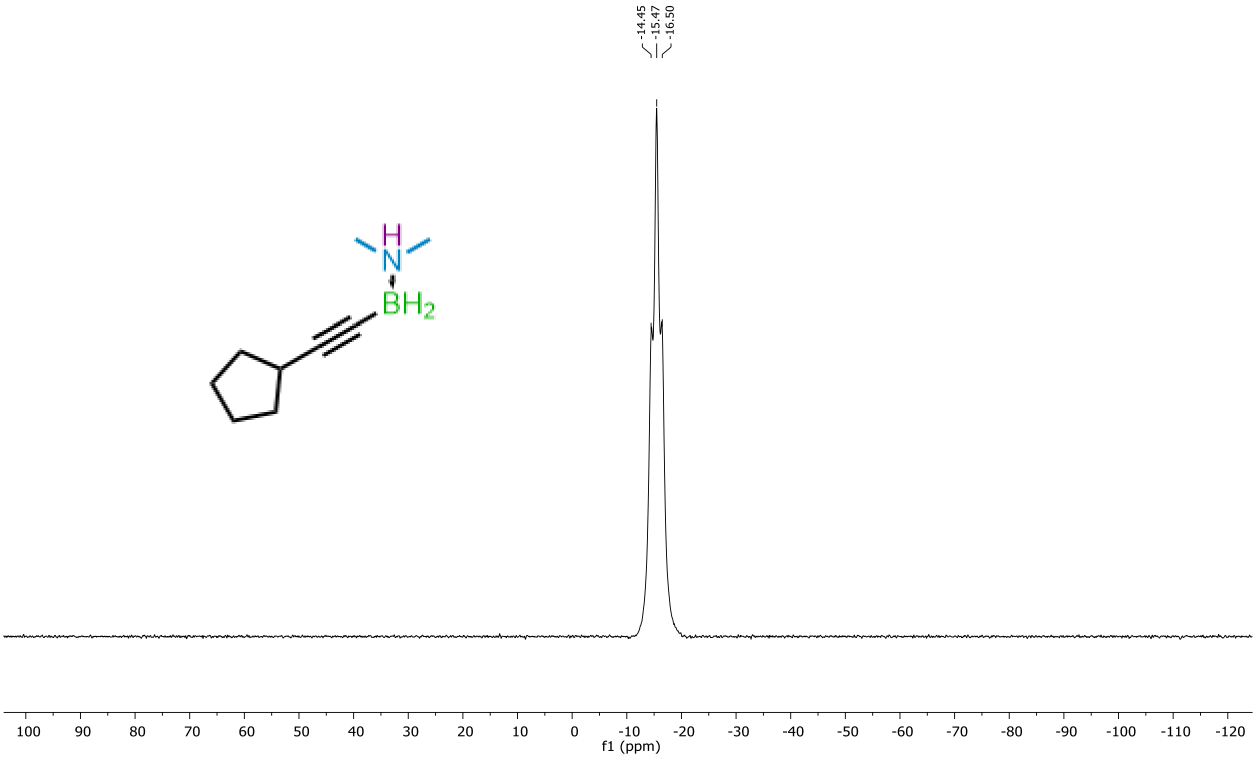

^{11}B NMR (96 MHz, $CDCl_3$) ((cyclopentyl)ethynyl)borane-dimethylamine (**3le**)

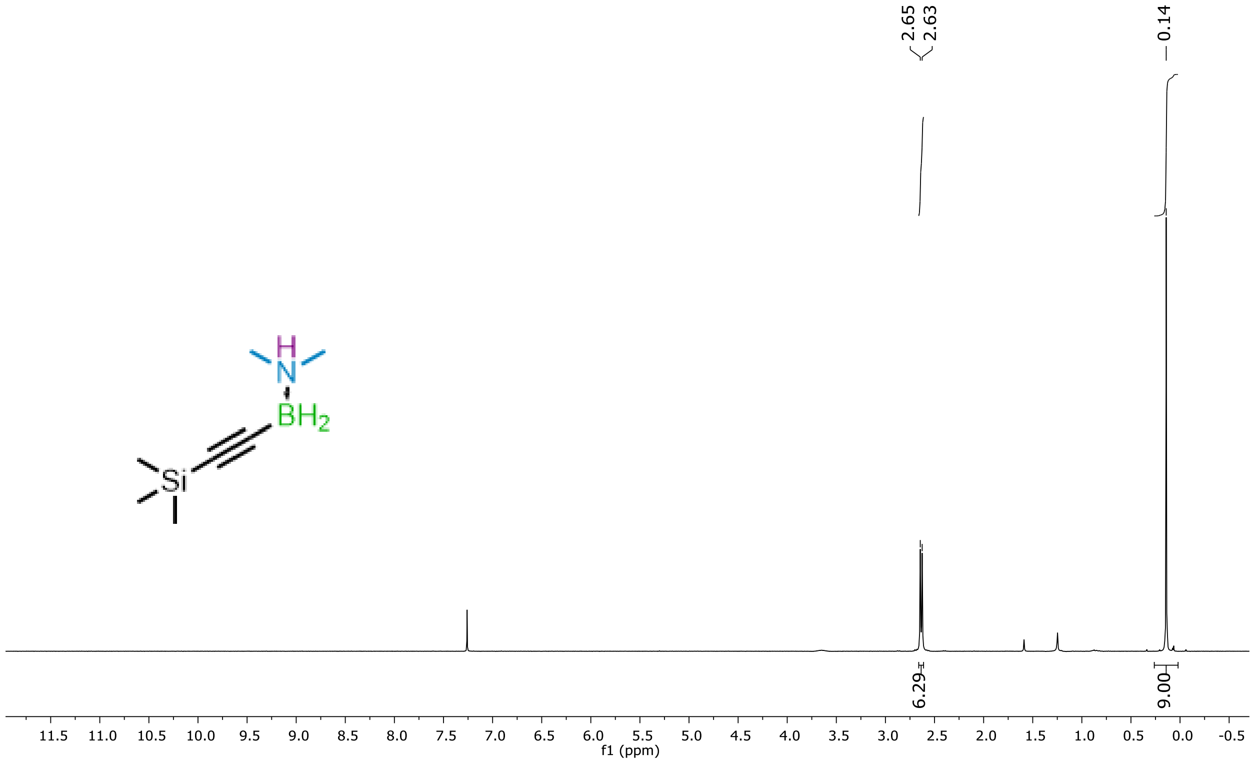

^{1}H NMR (300 MHz, $CDCl_3$) ((trimethylsilyl)ethynyl)borane-dimethylamine (**3me**)

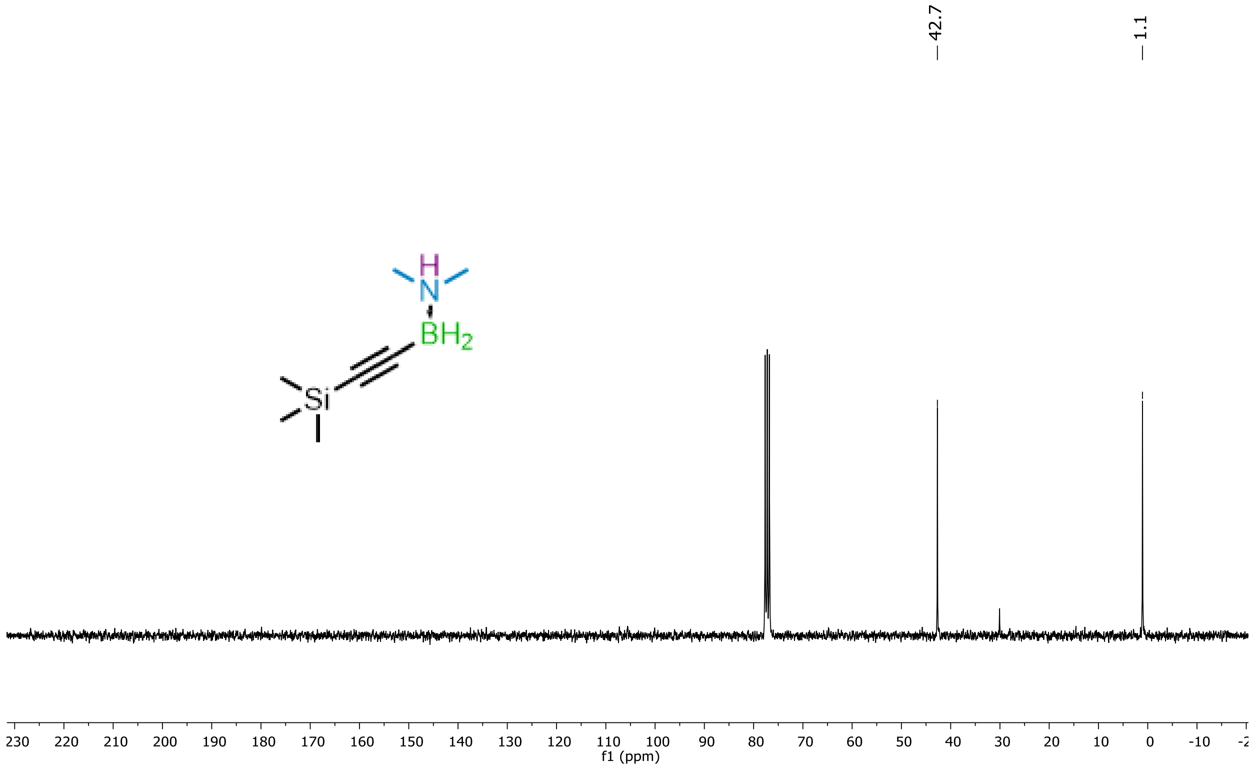

^{13}C NMR (75 MHz, $CDCl_3$) ((trimethylsilyl)ethynyl)borane-dimethylamine (**3me**)

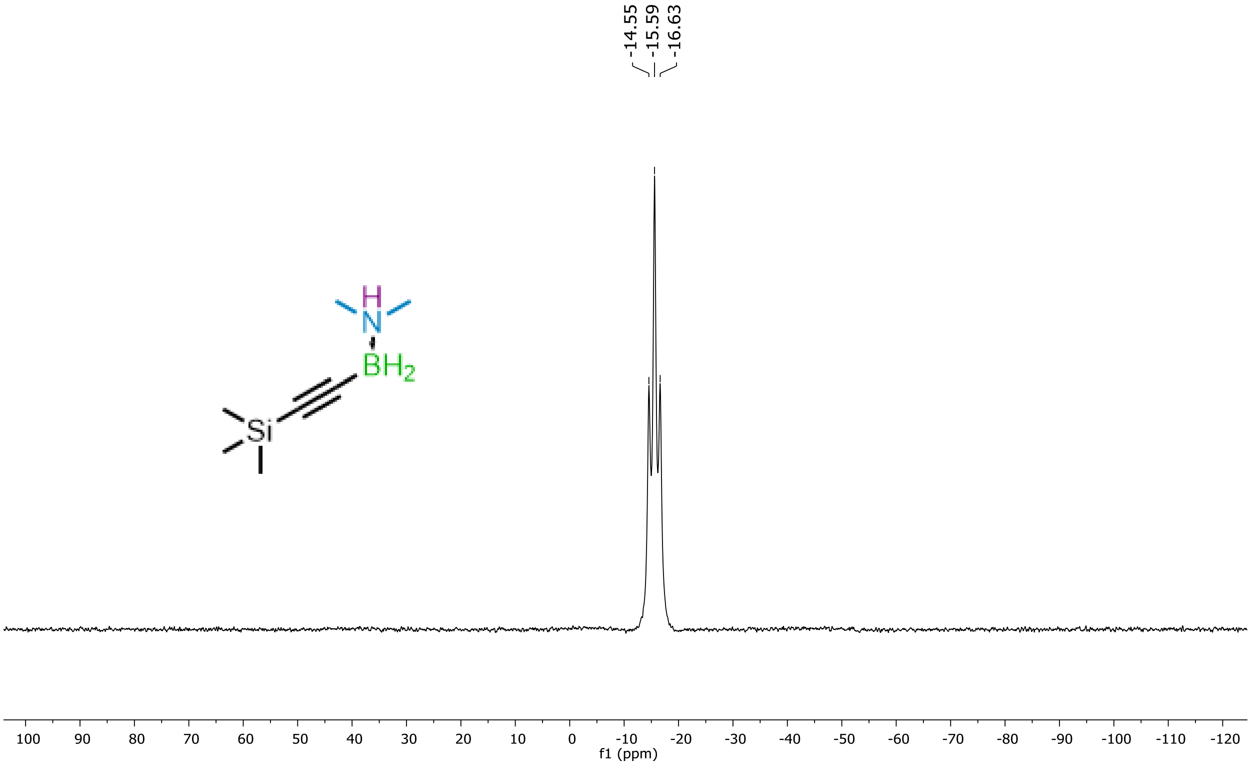

^{11}B NMR (96 MHz, $CDCl_3$) ((trimethylsilyl)ethynyl)borane-dimethylamine (**3me**)

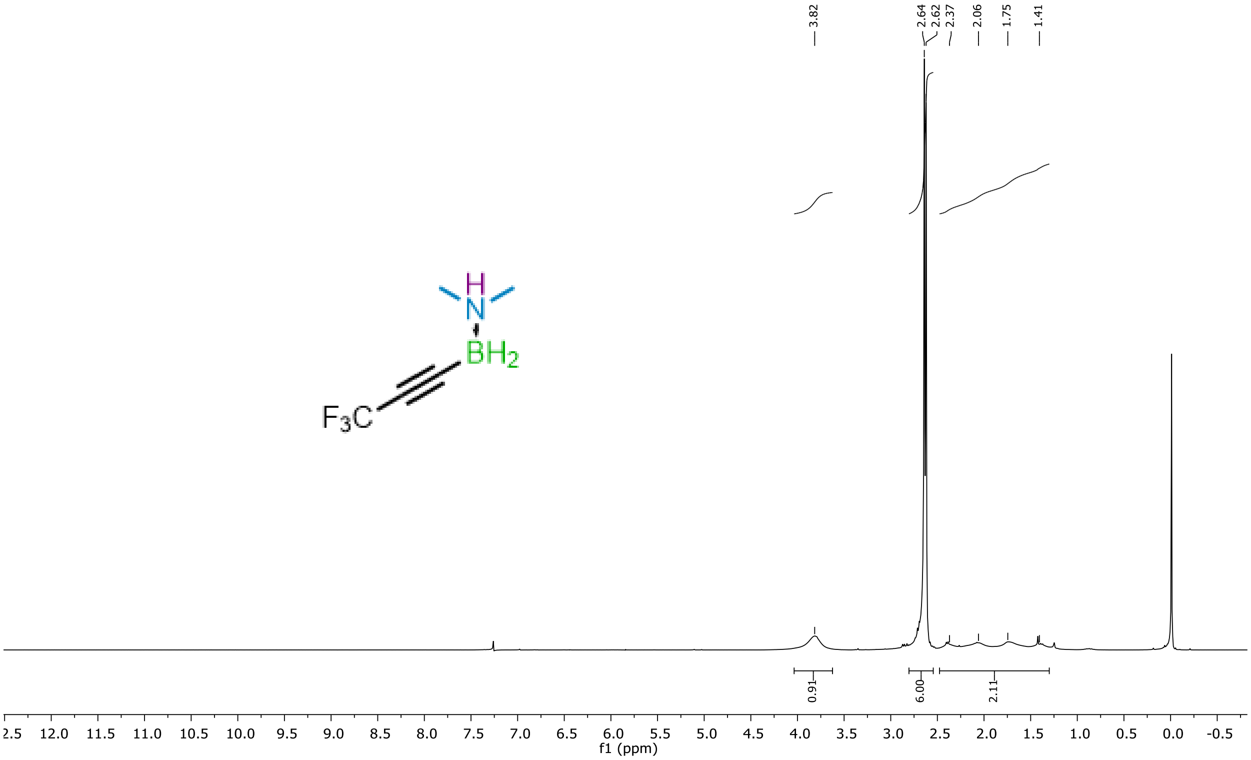

^{1}H NMR (300 MHz, $CDCl_3$) (3, 3, 3-trifluoroprop-1-yn-1-yl)borane-dimethylamine (**3ne**)

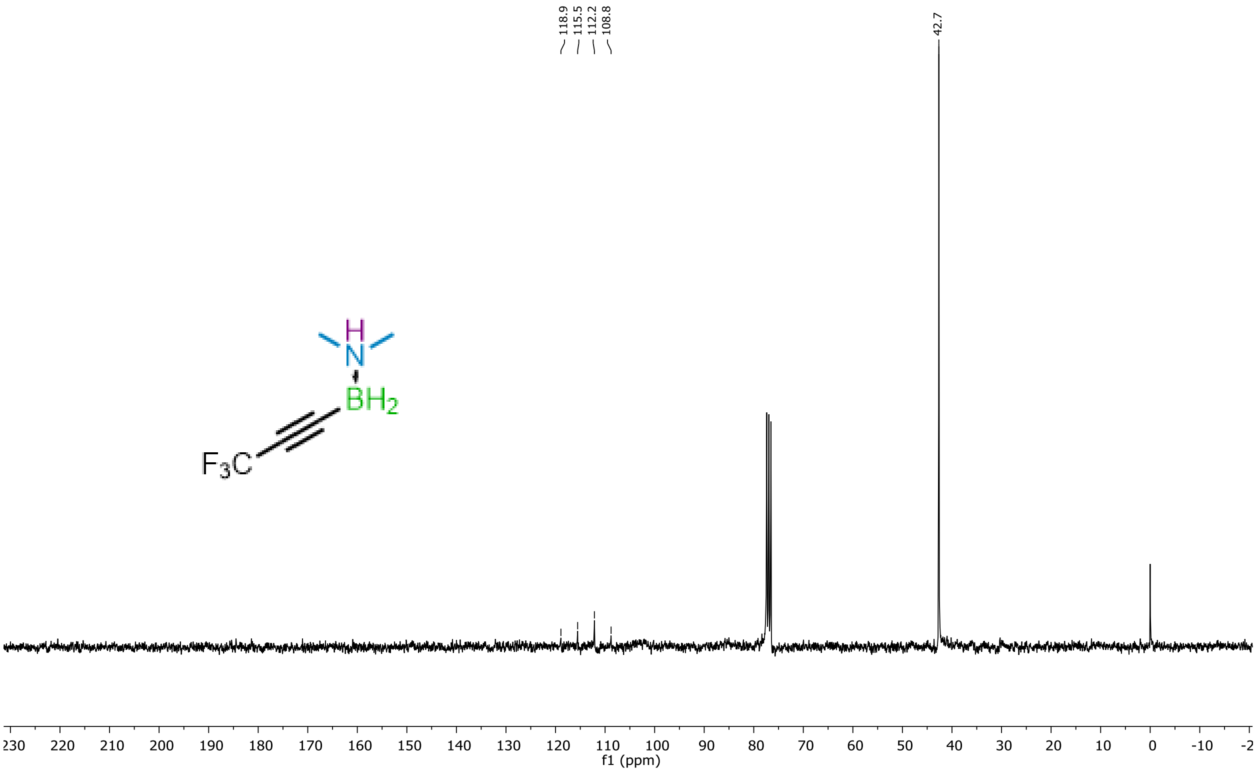

^{13}C NMR (75 MHz, $CDCl_3$) (3, 3, 3-trifluoroprop-1-yn-1-yl)borane-dimethylamine (**3ne**)

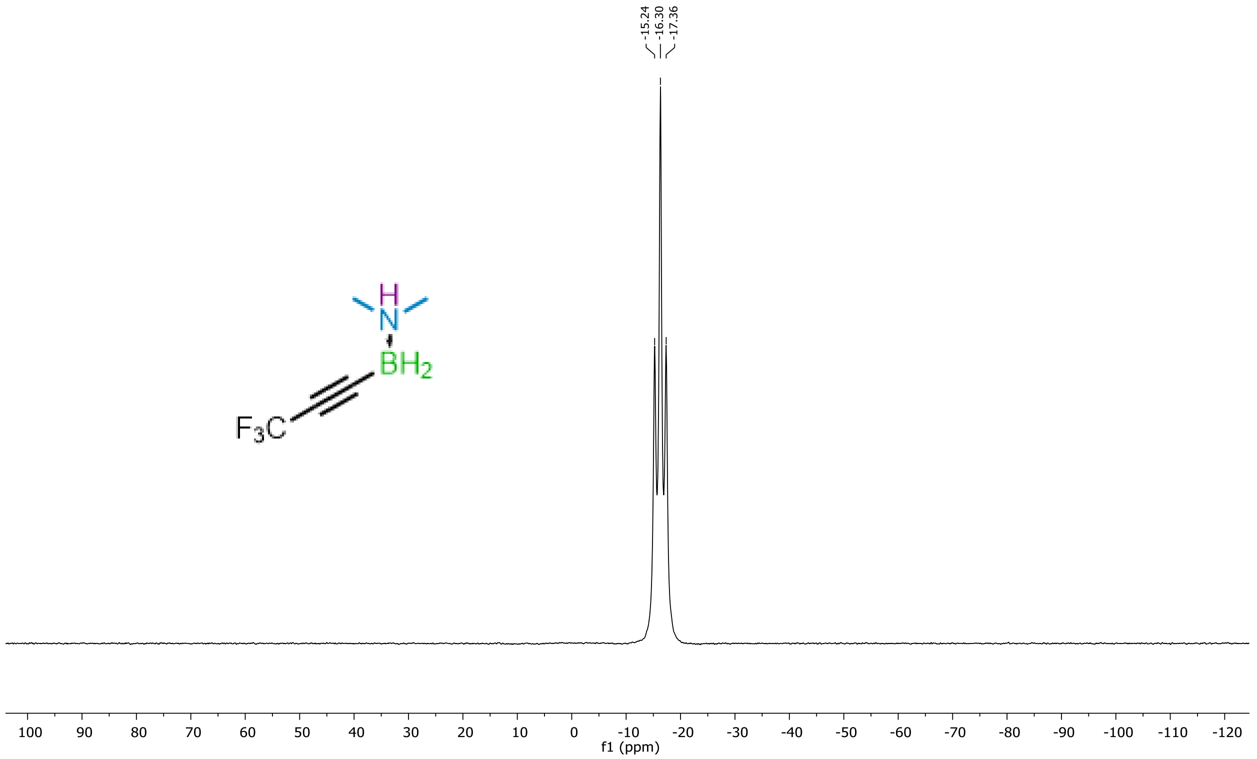

^{11}B NMR (96 MHz, $CDCl_3$) (3, 3, 3-trifluoroprop-1-yn-1-yl)borane-dimethylamine (**3ne**)

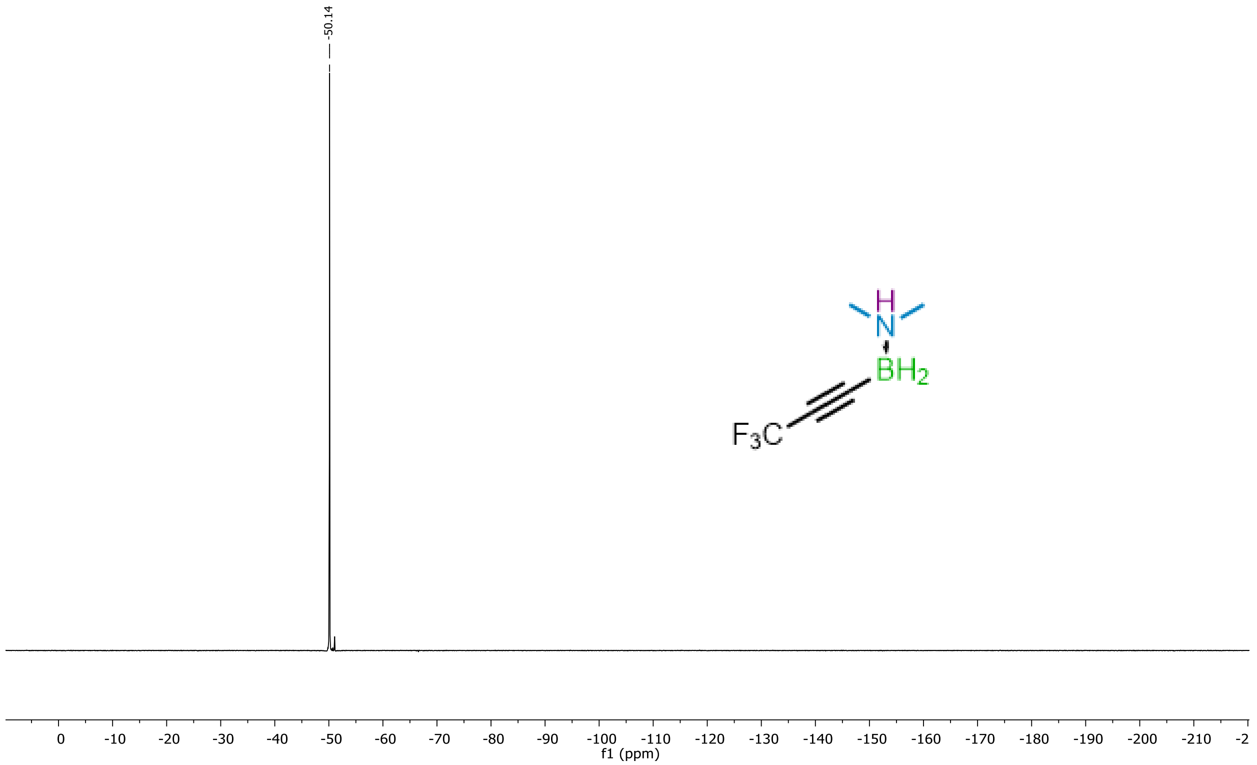

^{19}F NMR (282 MHz, $CDCl_3$) (3, 3, 3-trifluoroprop-1-yn-1-yl)borane-dimethylamine (**3ne**)

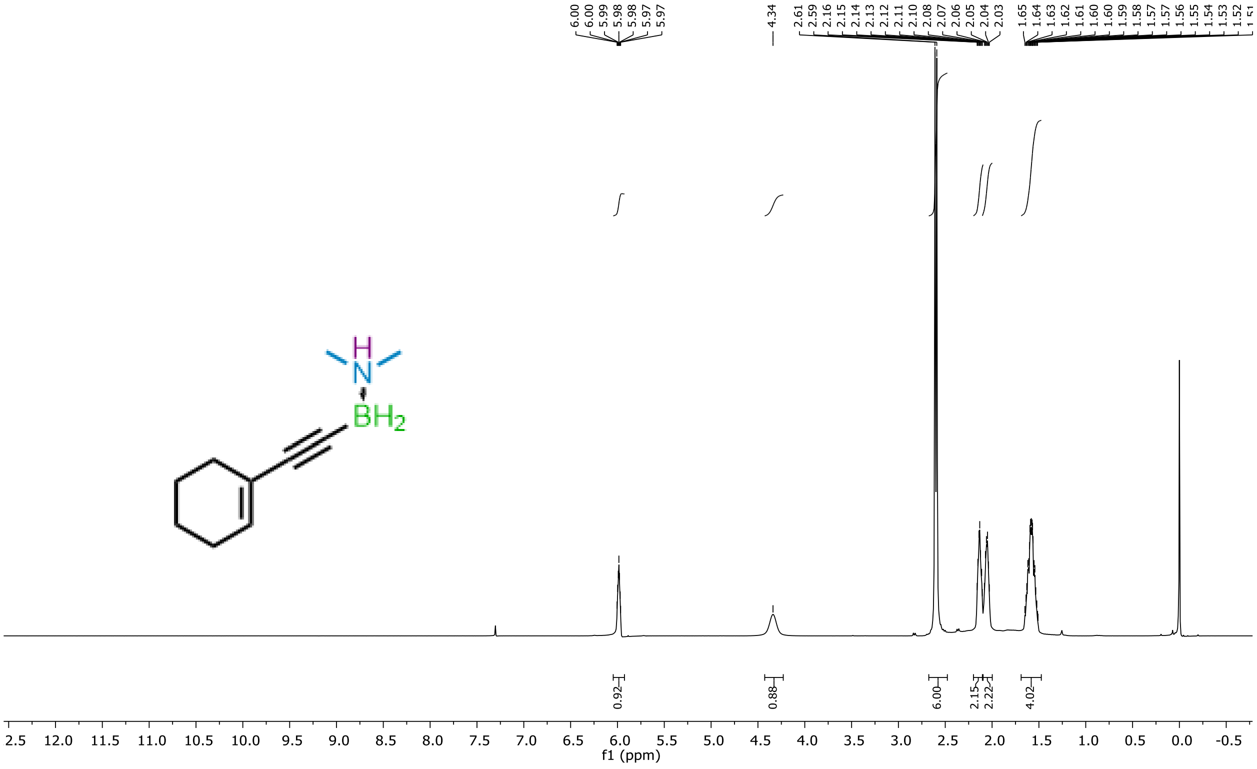

^{1}H NMR (300 MHz, $CDCl_3$) ((cyclohex-1-en-1-yl)ethynyl)borane-dimethylamine (**3oe**)

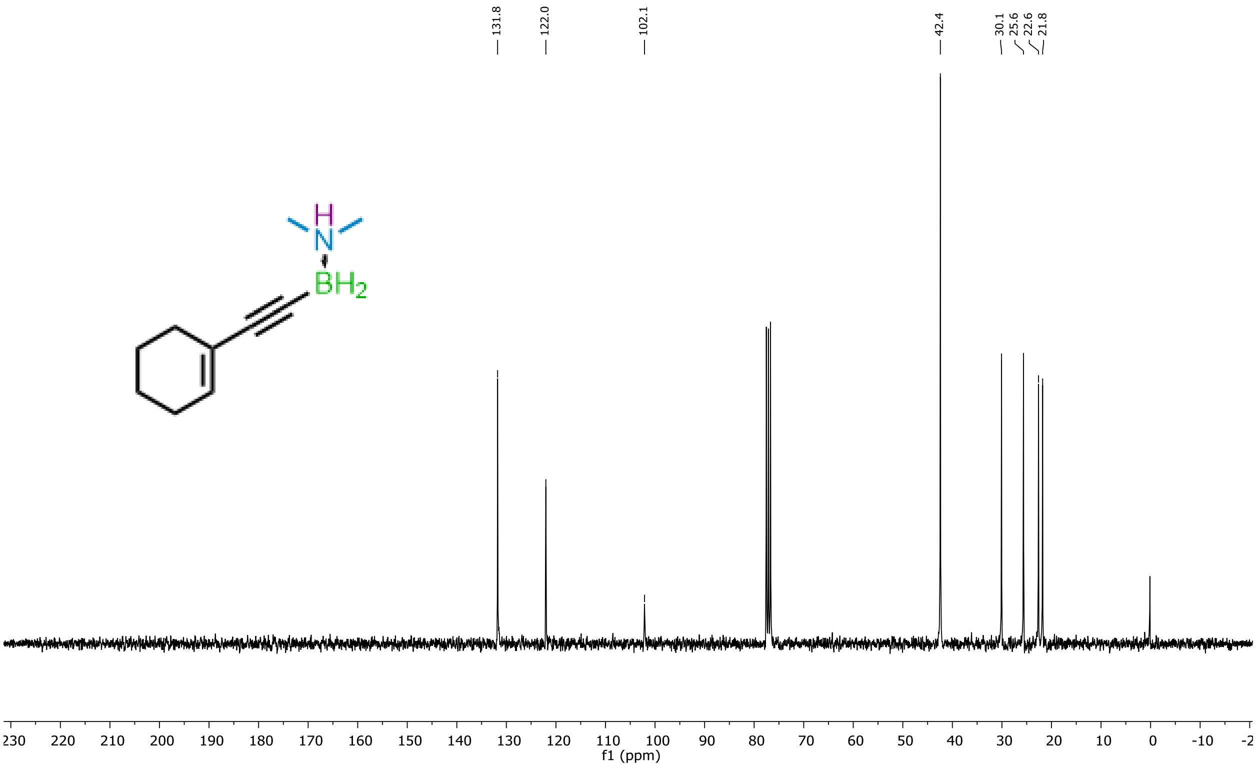

^{13}C NMR (75 MHz, $CDCl_3$) ((cyclohex-1-en-1-yl)ethynyl)borane-dimethylamine (**3oe**)

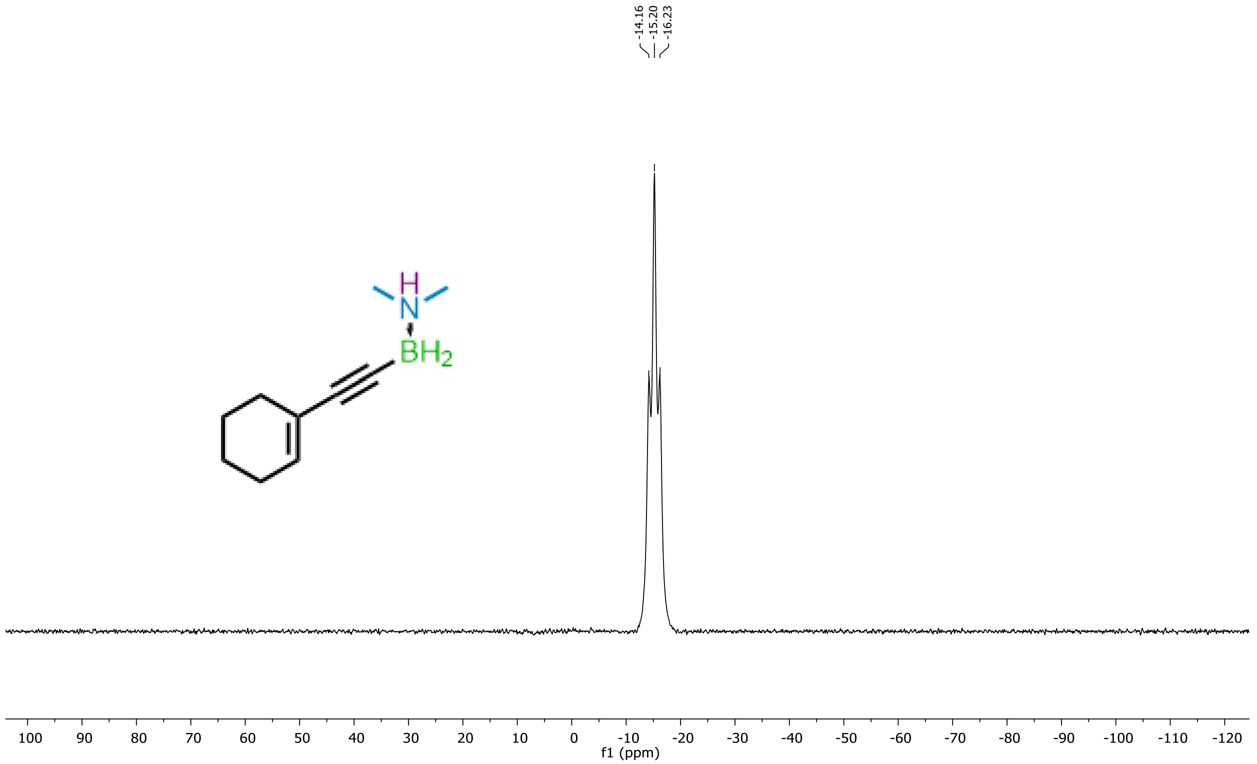

^{11}B NMR (96 MHz, $CDCl_3$) ((cyclohex-1-en-1-yl)ethynyl)borane-dimethylamine (**3oe**)

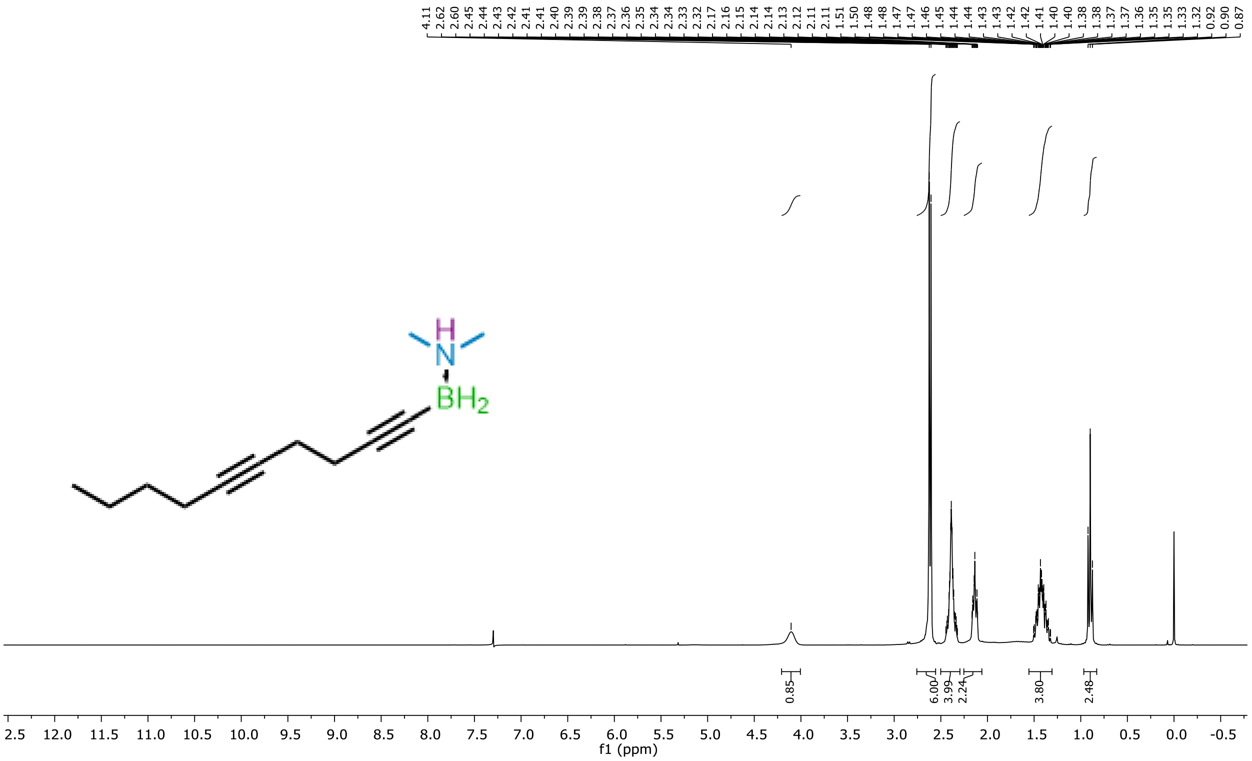

^{1}H NMR (300 MHz, $CDCl_3$) (deca-1, 5-diyn-1-yl)borane-dimethylamine (**3pe**)

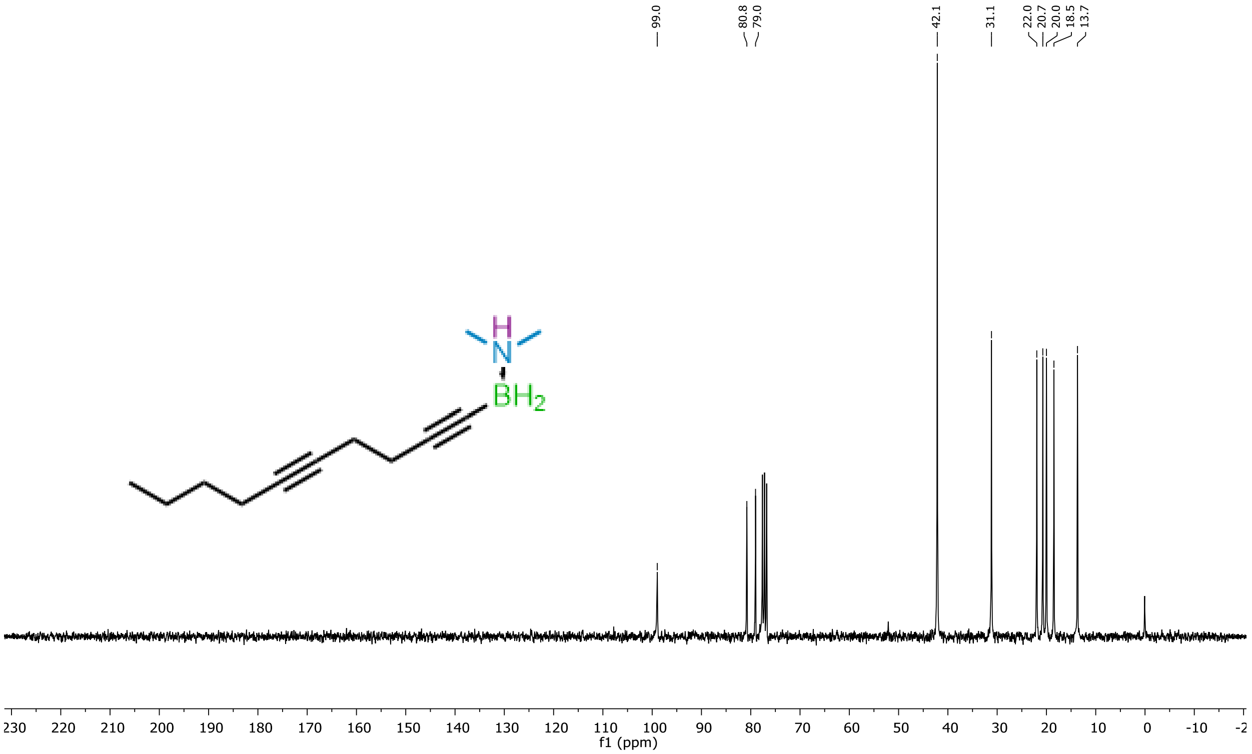

^{13}C NMR (75 MHz, $CDCl_3$) (deca-1, 5-diyn-1-yl)borane-dimethylamine (**3pe**)

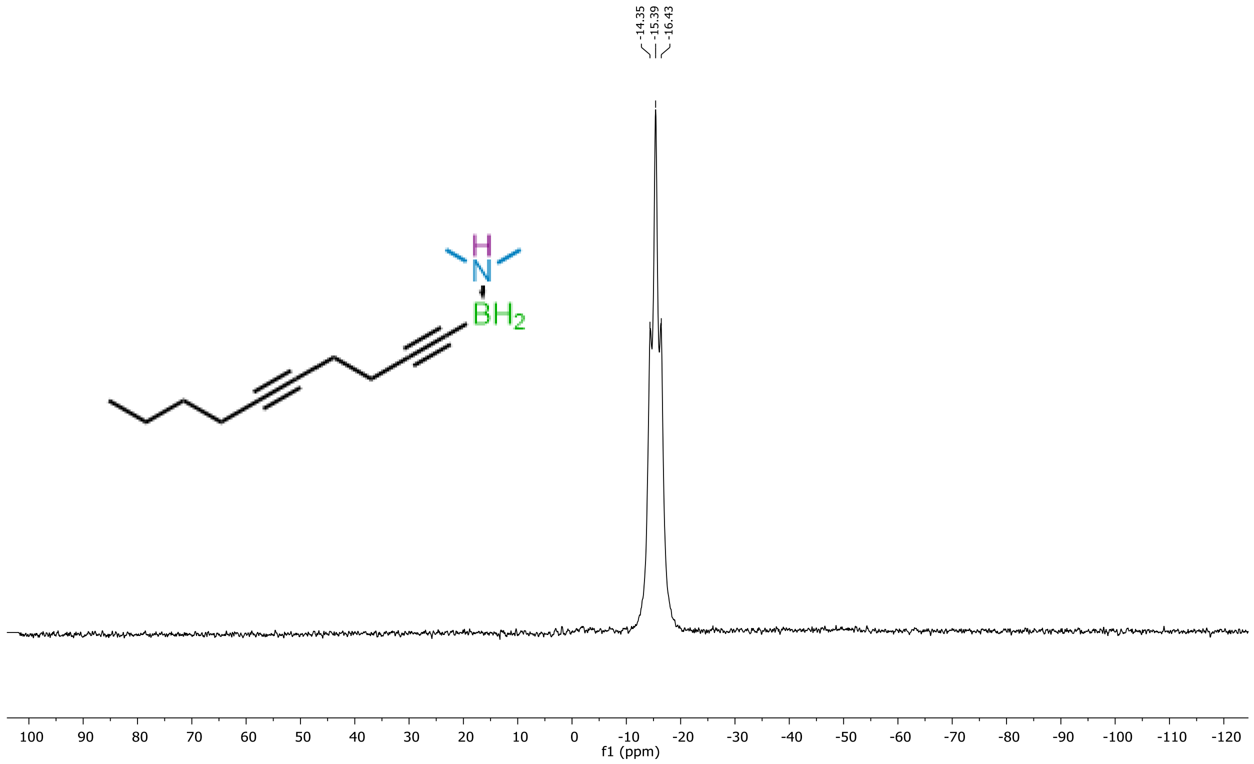

^{11}B NMR (96 MHz, $CDCl_3$) (deca-1, 5-diyn-1-yl)borane-dimethylamine (**3pe**)

<u>NMR spectra of dibromides</u>

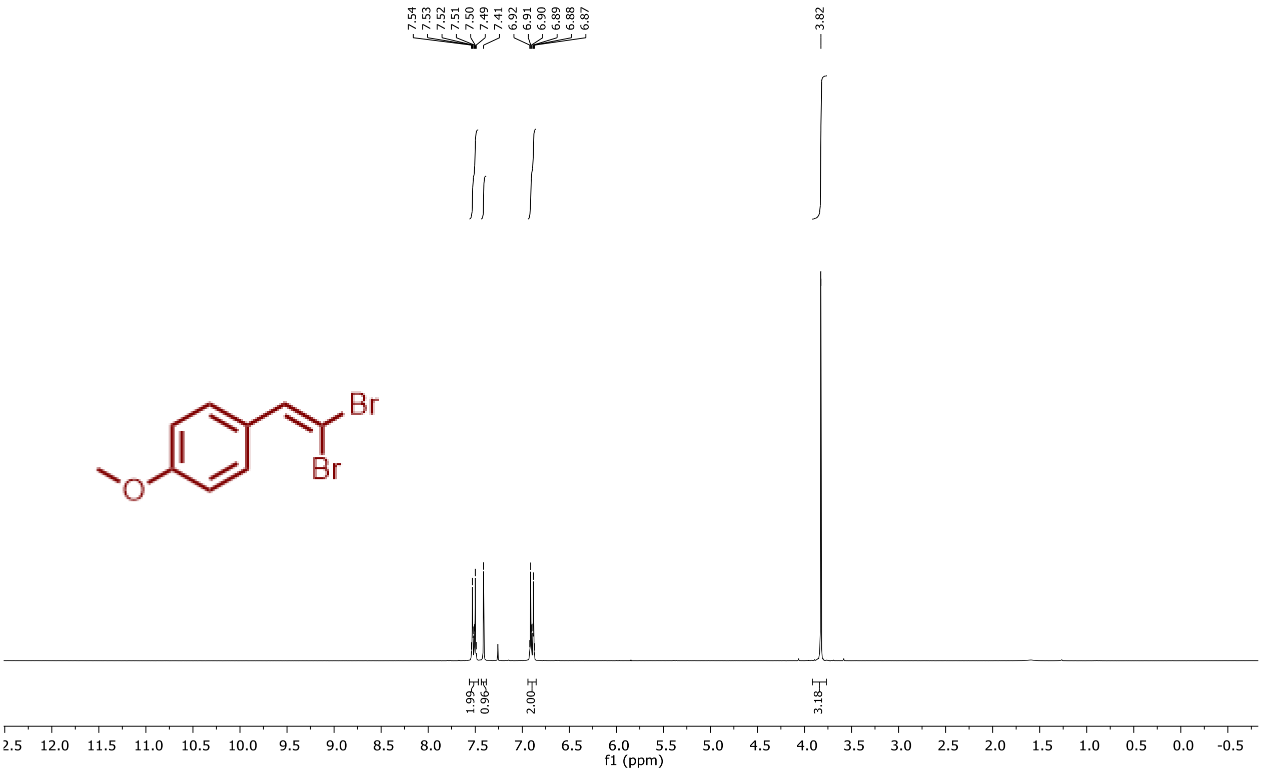

^{1}H NMR (300 MHz, $CDCl_3$) 1-(2,2-dibromovinyl)-4-methoxybenzene (**6a**)

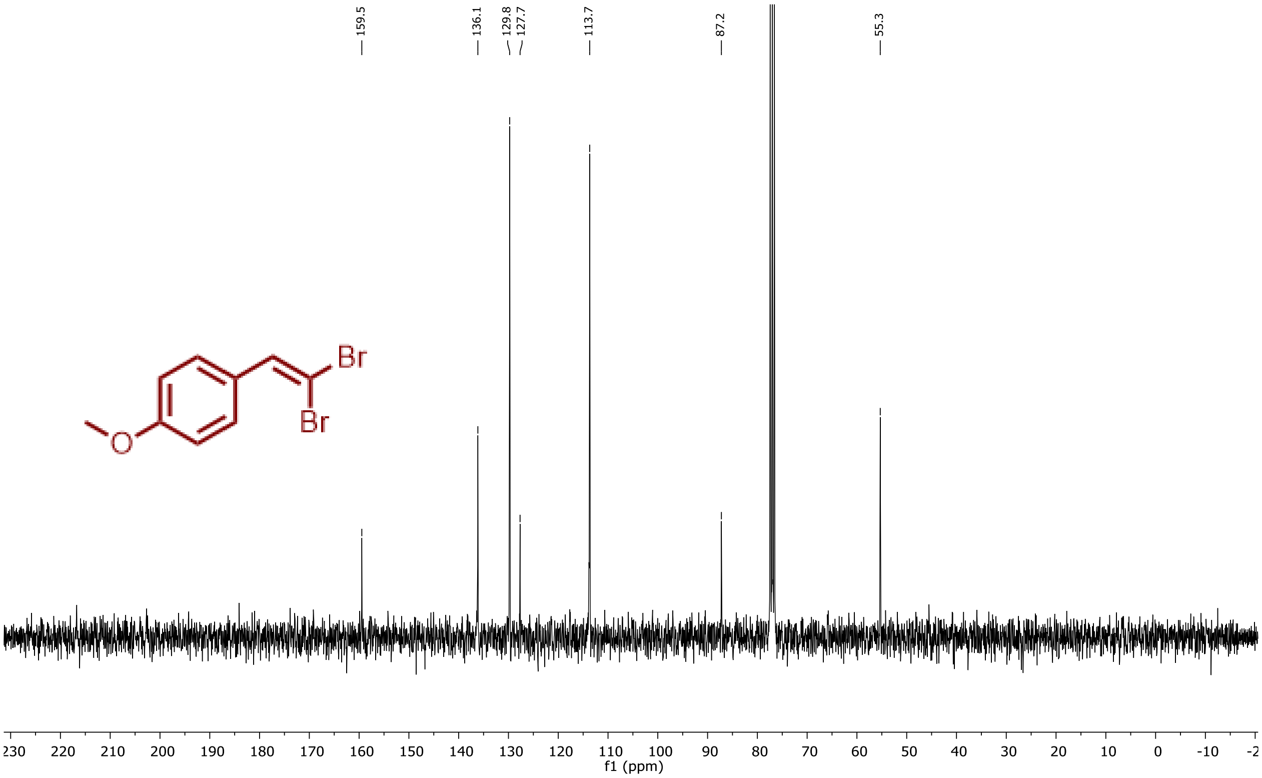

^{13}C NMR (75 MHz, $CDCl_3$) 1-(2,2-dibromovinyl)-4-methoxybenzene (**6a**)

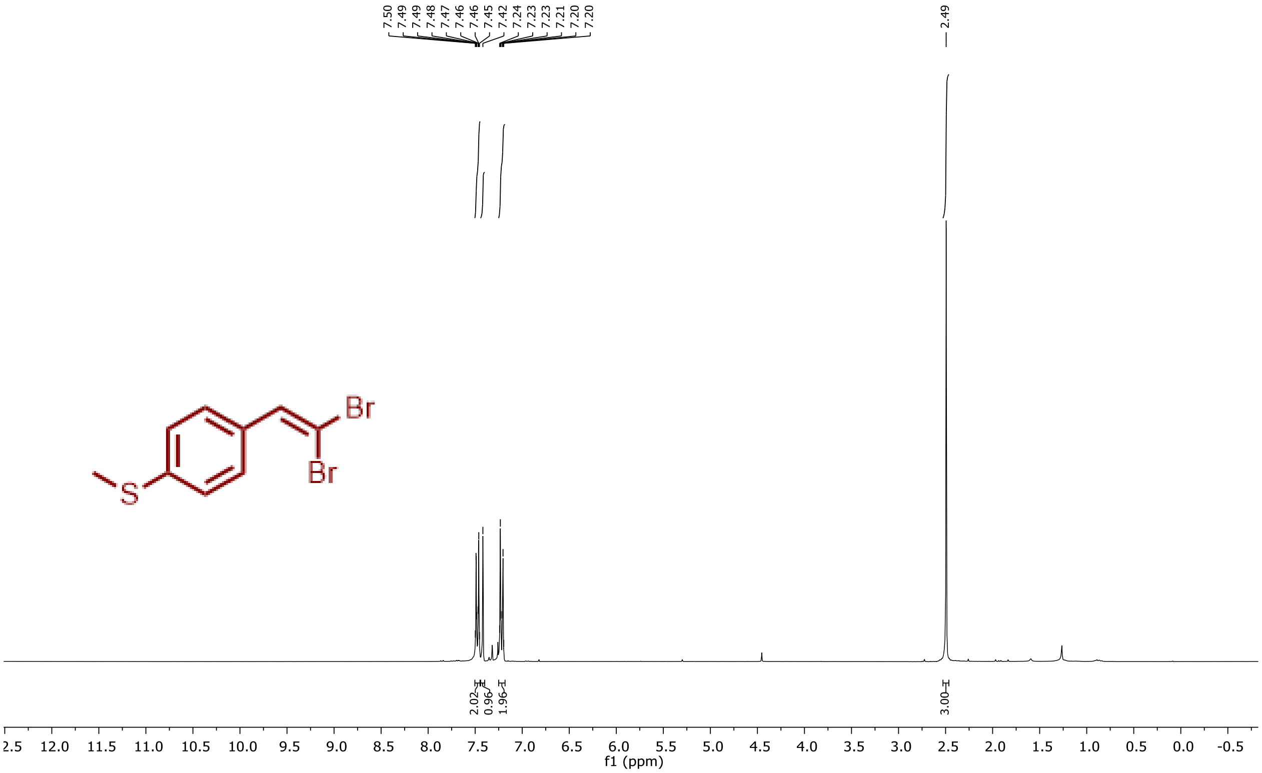

^{1}H NMR (300 MHz, $CDCl_3$) (4-(2,2-dibromovinyl)phenyl)(methyl)sulfane (**6b**)

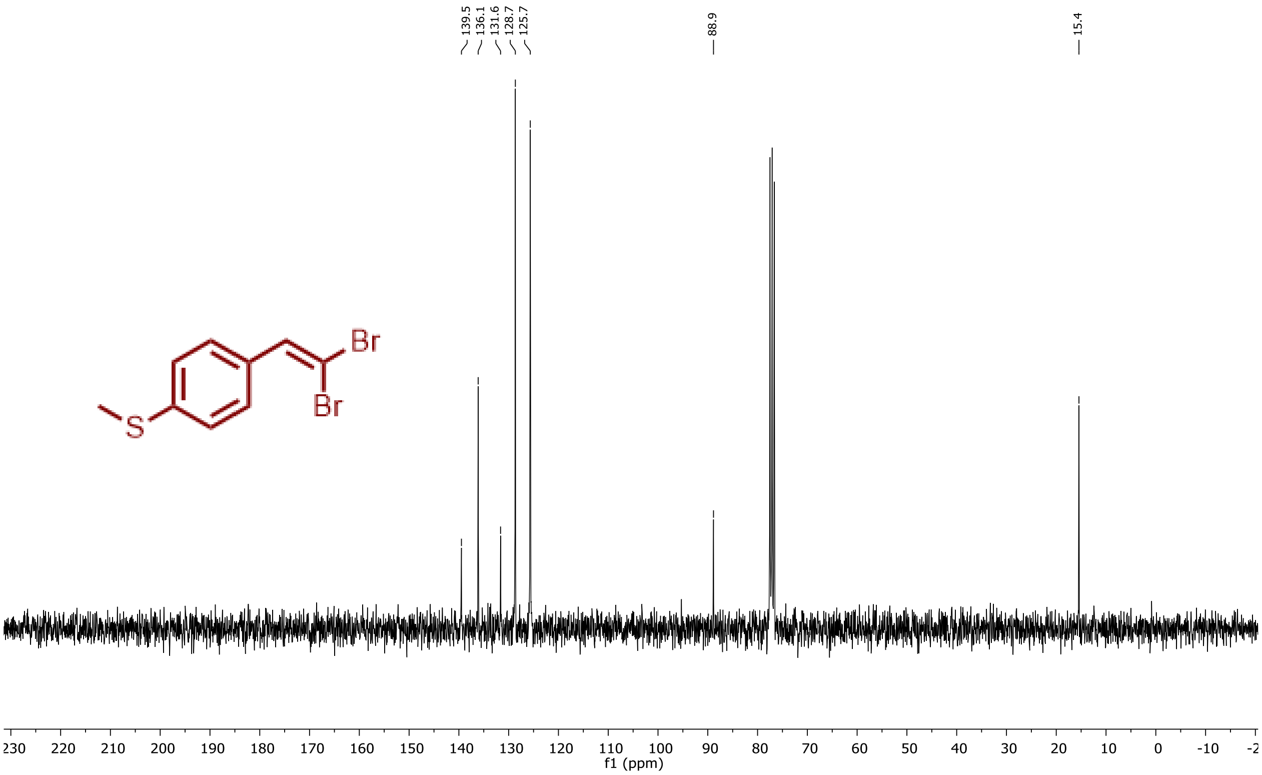

^{13}C NMR (75 MHz, $CDCl_3$) (4-(2,2-dibromovinyl)phenyl)(methyl)sulfane (**6b**)

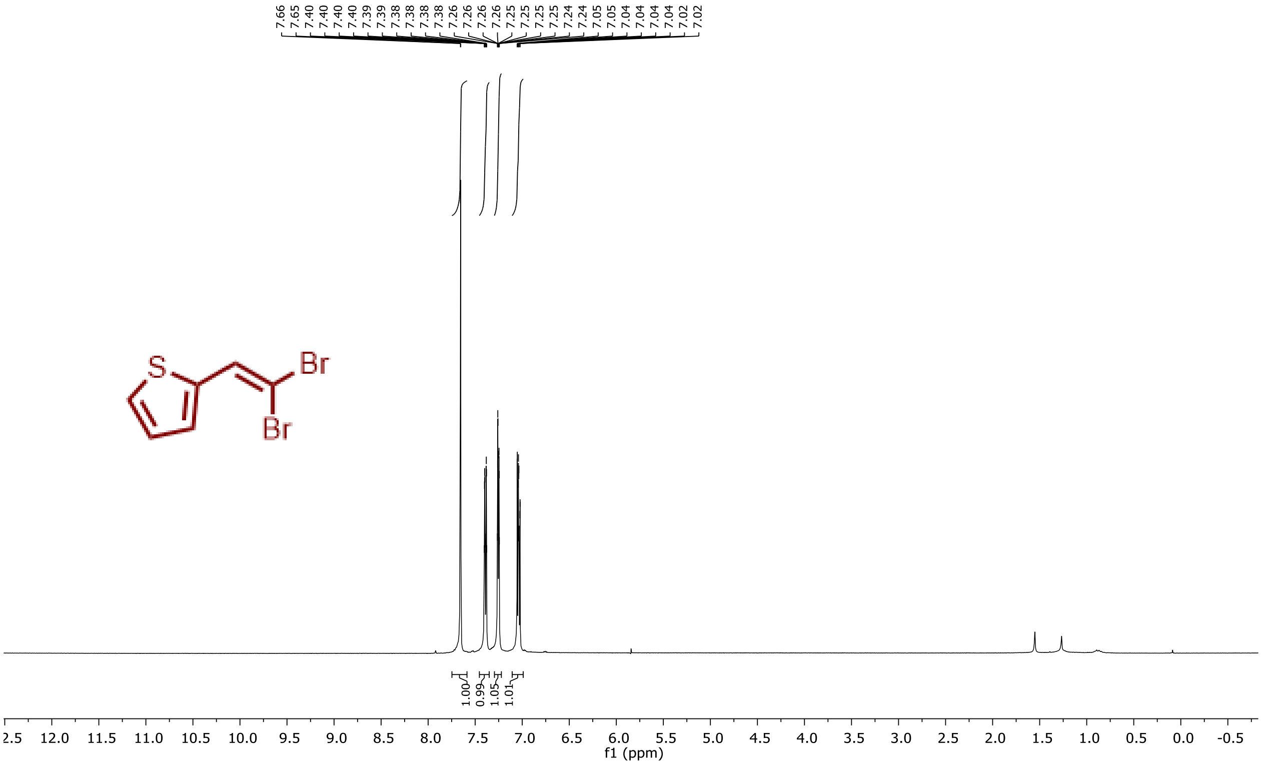

^{1}H NMR (300 MHz, $CDCl_3$) 2-(2,2-dibromovinyl)thiophene (**6c**)

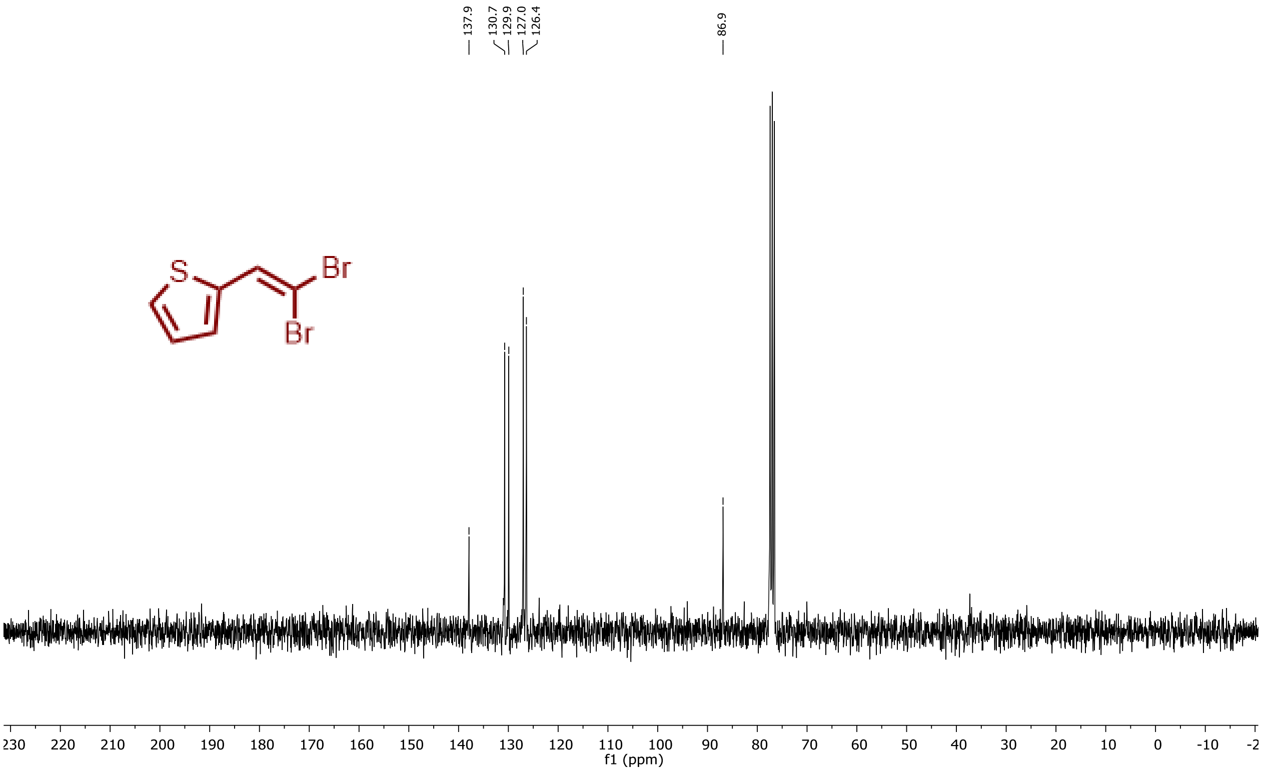

^{13}C NMR (75 MHz, $CDCl_3$) 2-(2,2-dibromovinyl)thiophene (**6c**)

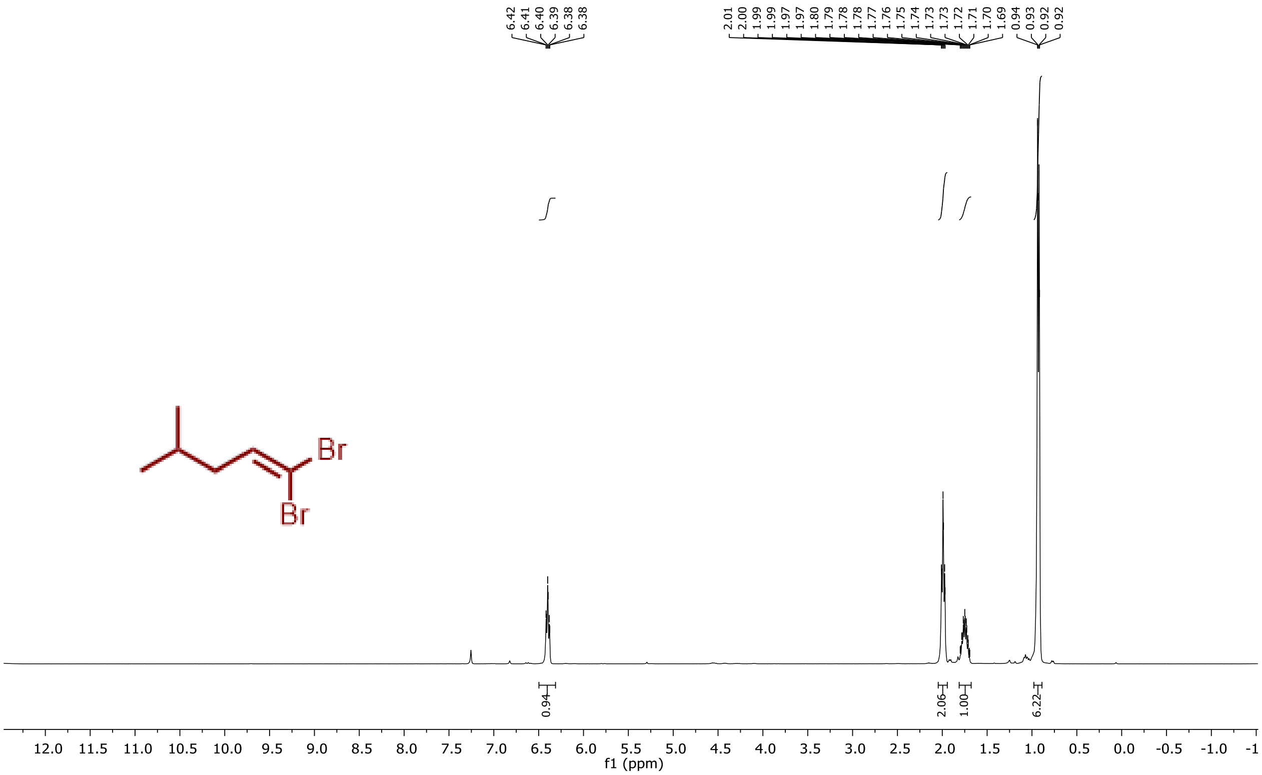

^{1}H NMR (400 MHz, $CDCl_3$) 1,1-dibromo-4-methylpent-1-ene (**6d**)

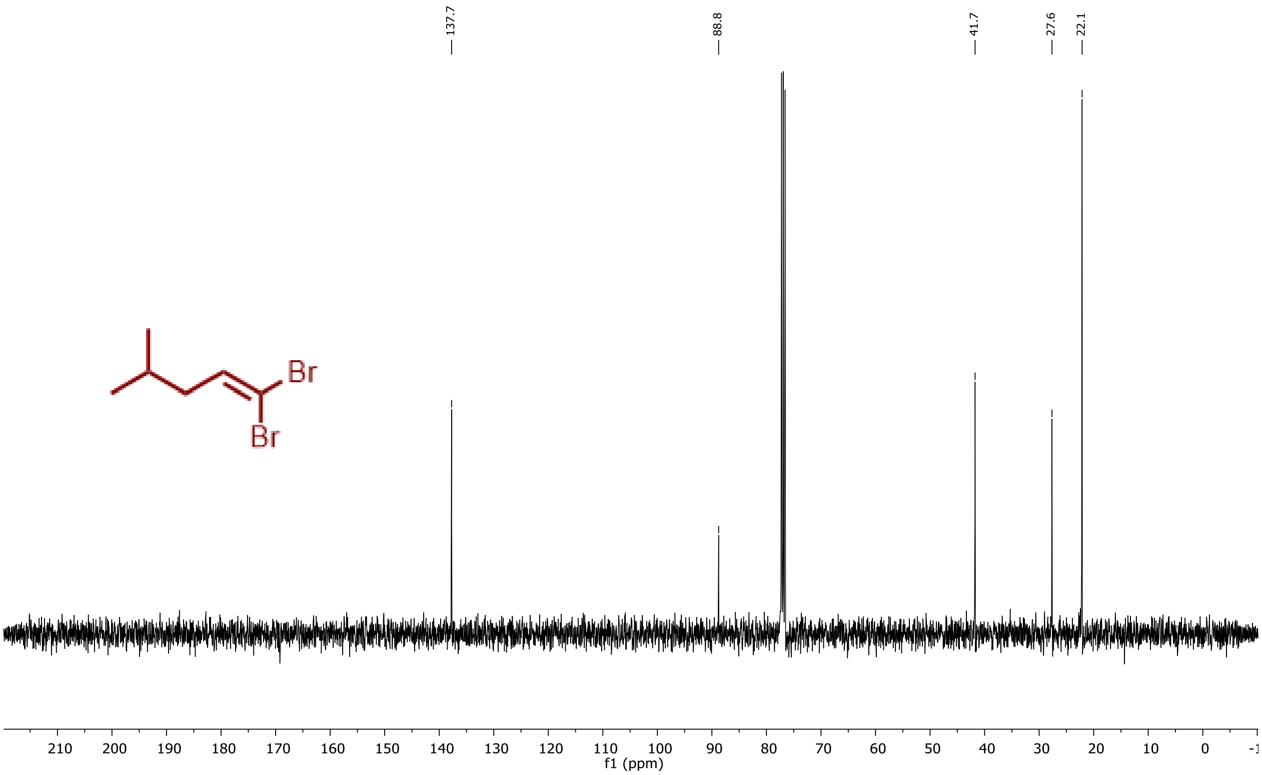

^{13}C NMR (101 MHz, $CDCl_3$) 1,1-dibromo-4-methylpent-1-ene (**6d**)

NMR spectra of alkynylborane-amines from dibromides

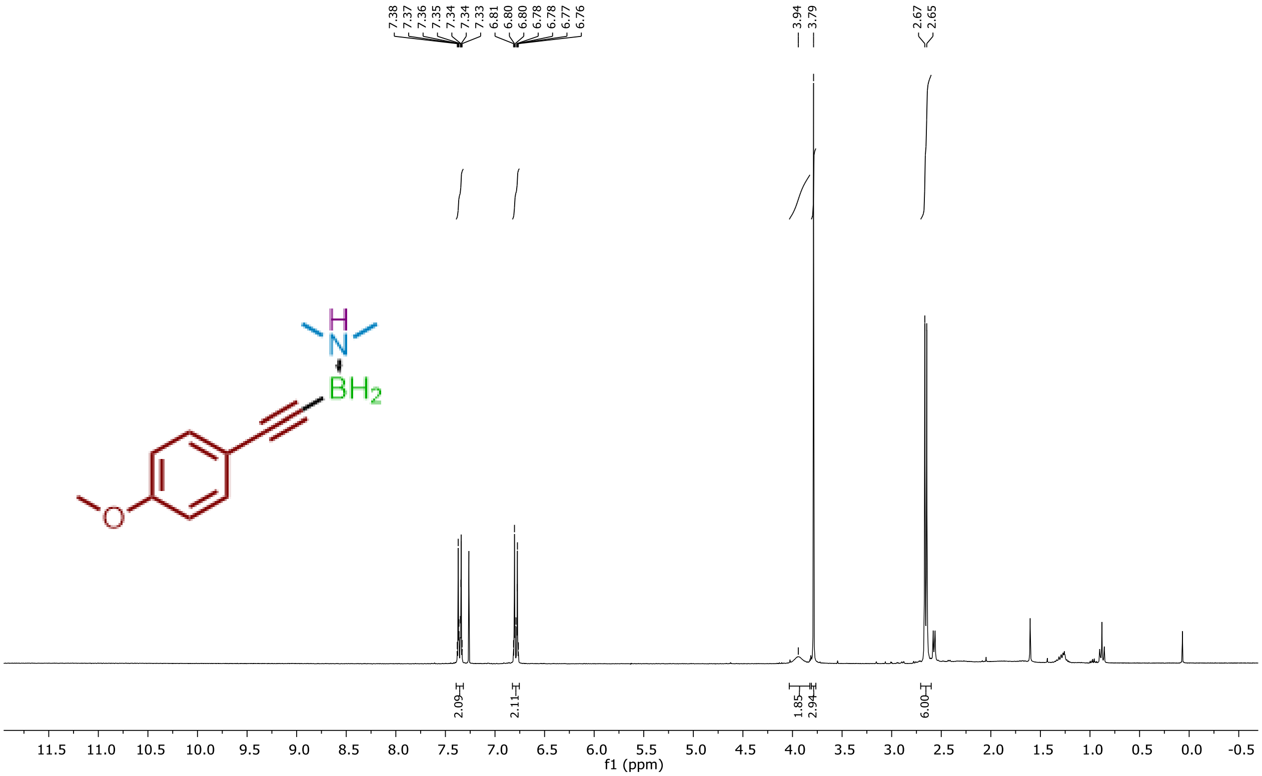

^{1}H NMR (300 MHz, $CDCl_3$) ((4-methoxyphenyl)ethynyl)borane-dimethylamine (**7ae**)

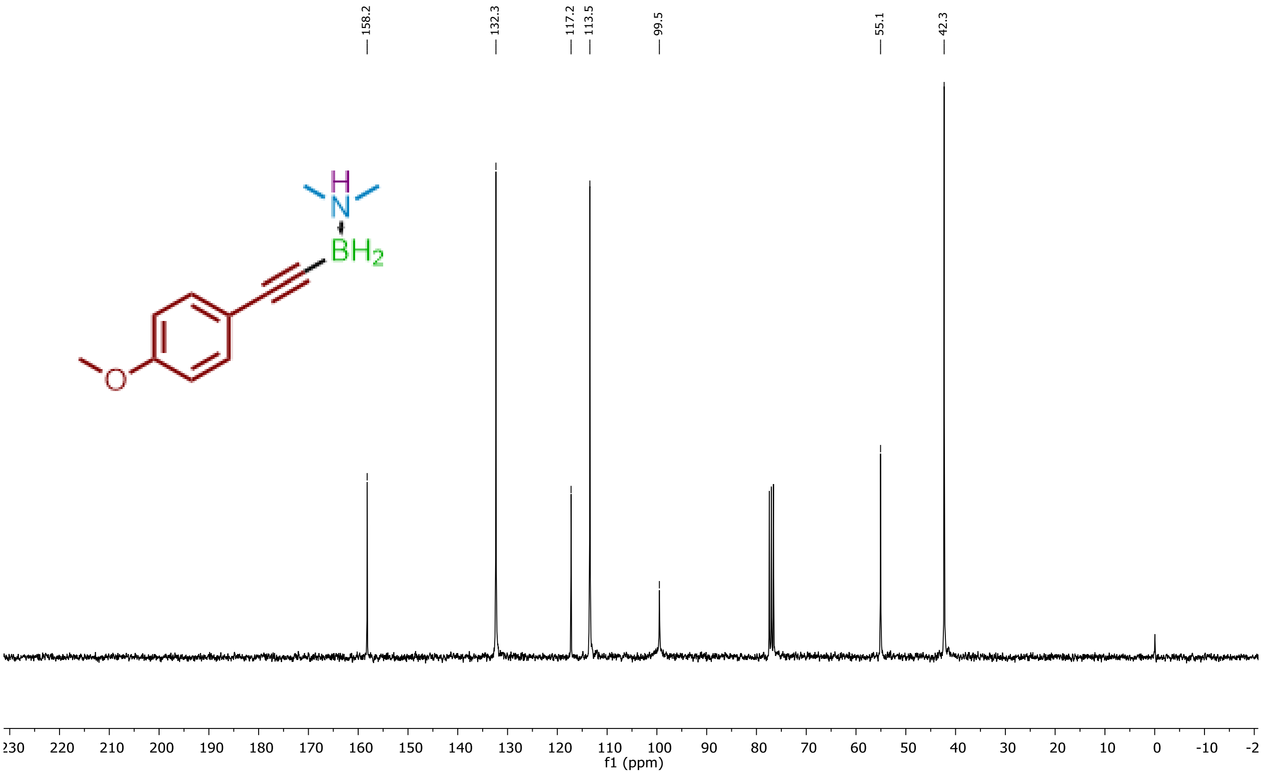

^{13}C NMR (75 MHz, $CDCl_3$) ((4-methoxyphenyl)ethynyl)borane-dimethylamine (**7ae**)

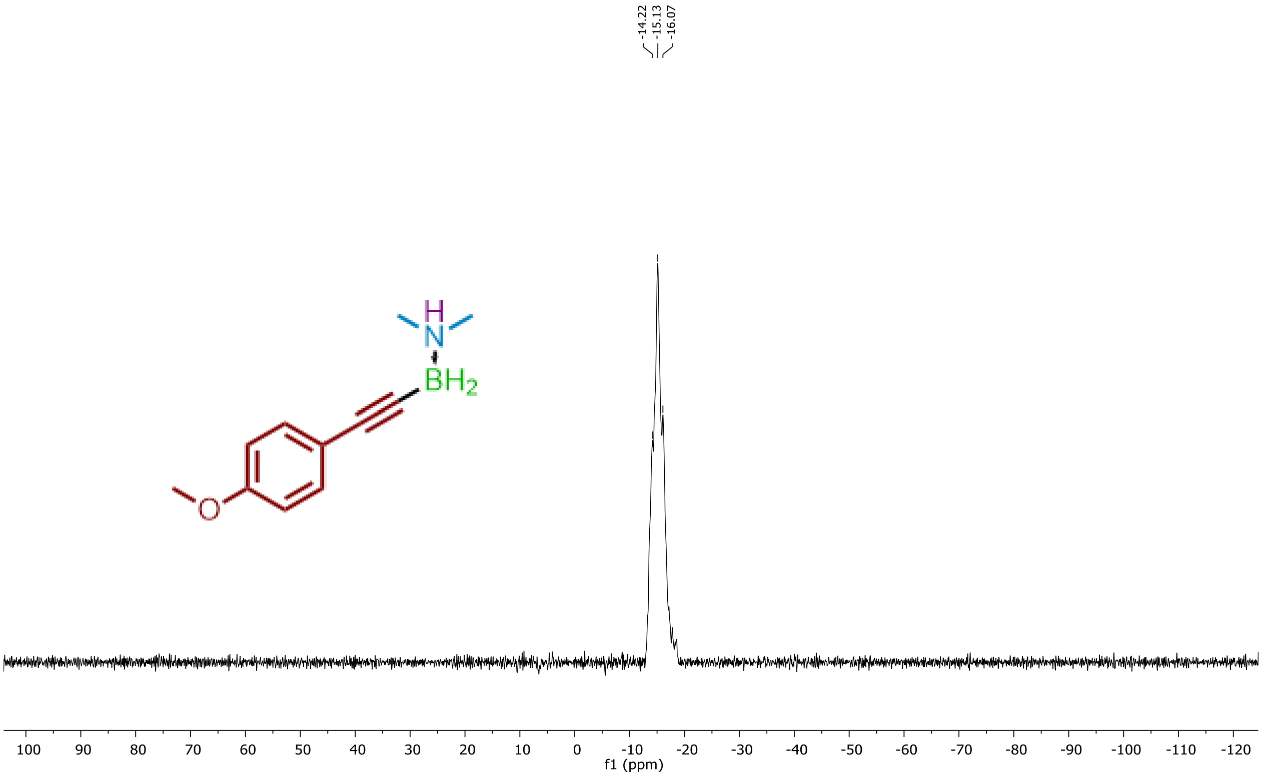

^{11}B NMR (96 MHz, $CDCl_3$) ((4-methoxyphenyl)ethynyl)borane-dimethylamine (**7ae**)

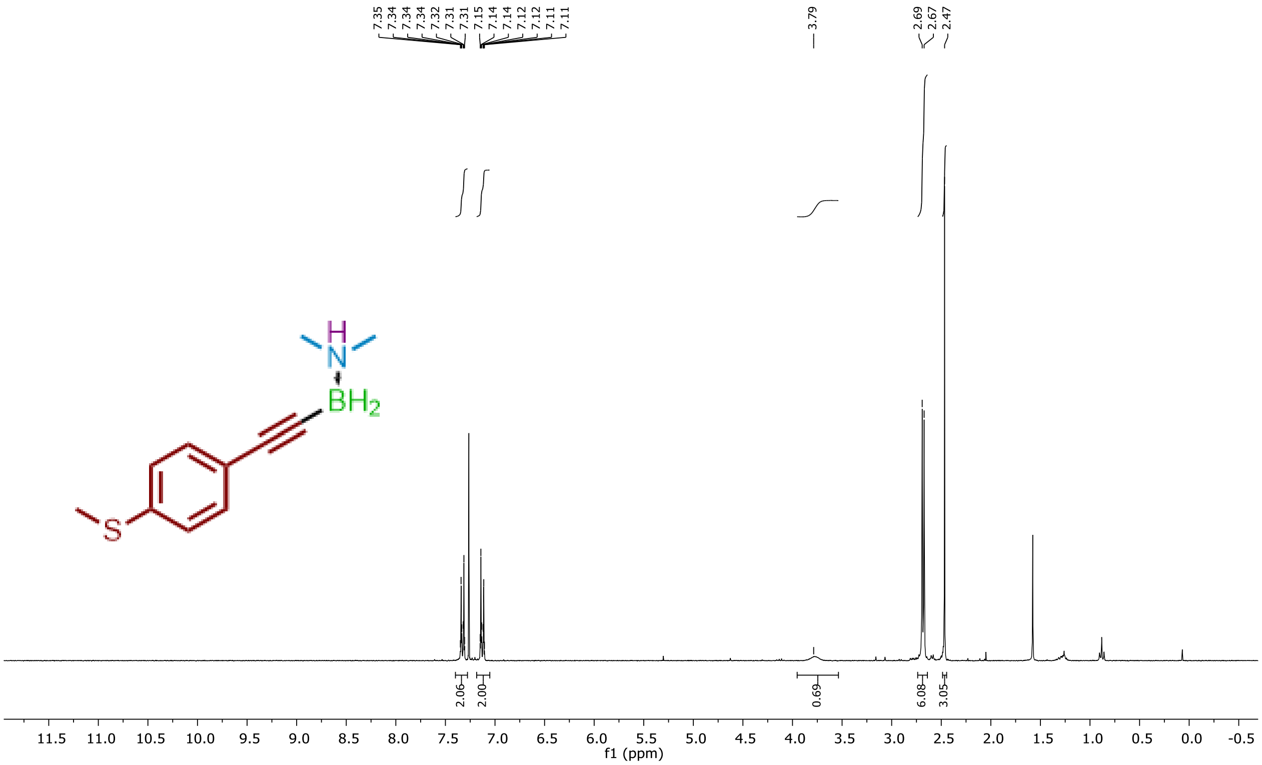

^{1}H NMR (300 MHz, $CDCl_3$) ((4-(methylthio)phenyl)ethynyl)borane-dimethylamine (**7be**)

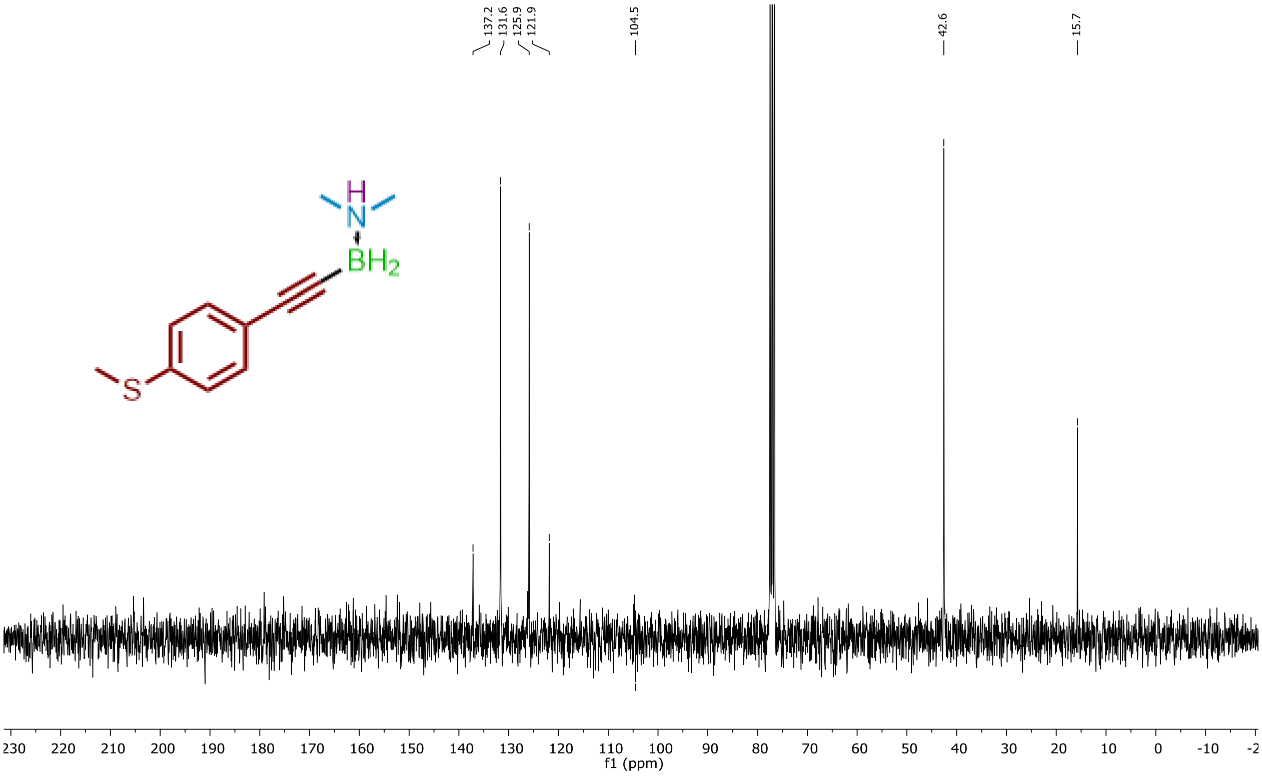

^{13}C NMR (75 MHz, $CDCl_3$) ((4-(methylthio)phenyl)ethynyl)borane-dimethylamine (**7be**)

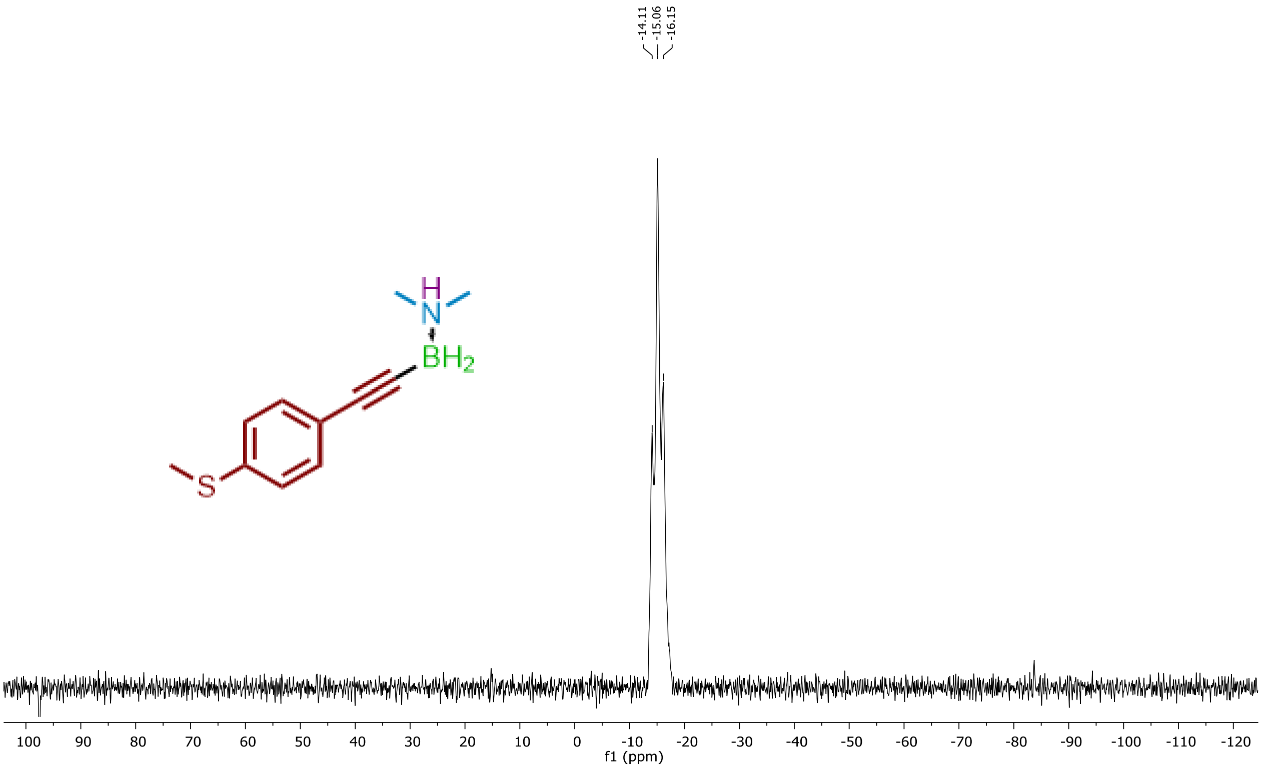

^{11}B NMR (96 MHz, $CDCl_3$) ((4-(methylthio)phenyl)ethynyl)borane-dimethylamine (**7be**)

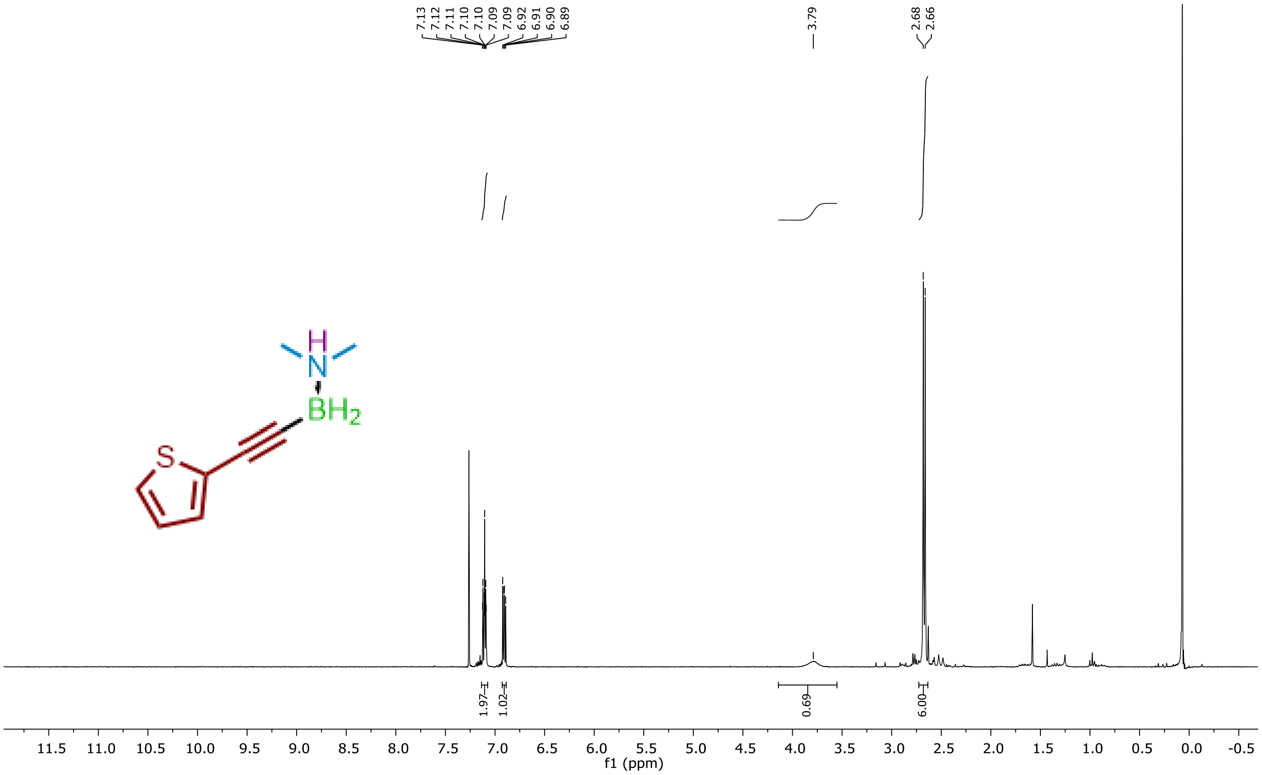

^{1}H NMR (300 MHz, $CDCl_3$) (thiophen-2-ylethynyl)borane-dimethylamine (**7ce**)

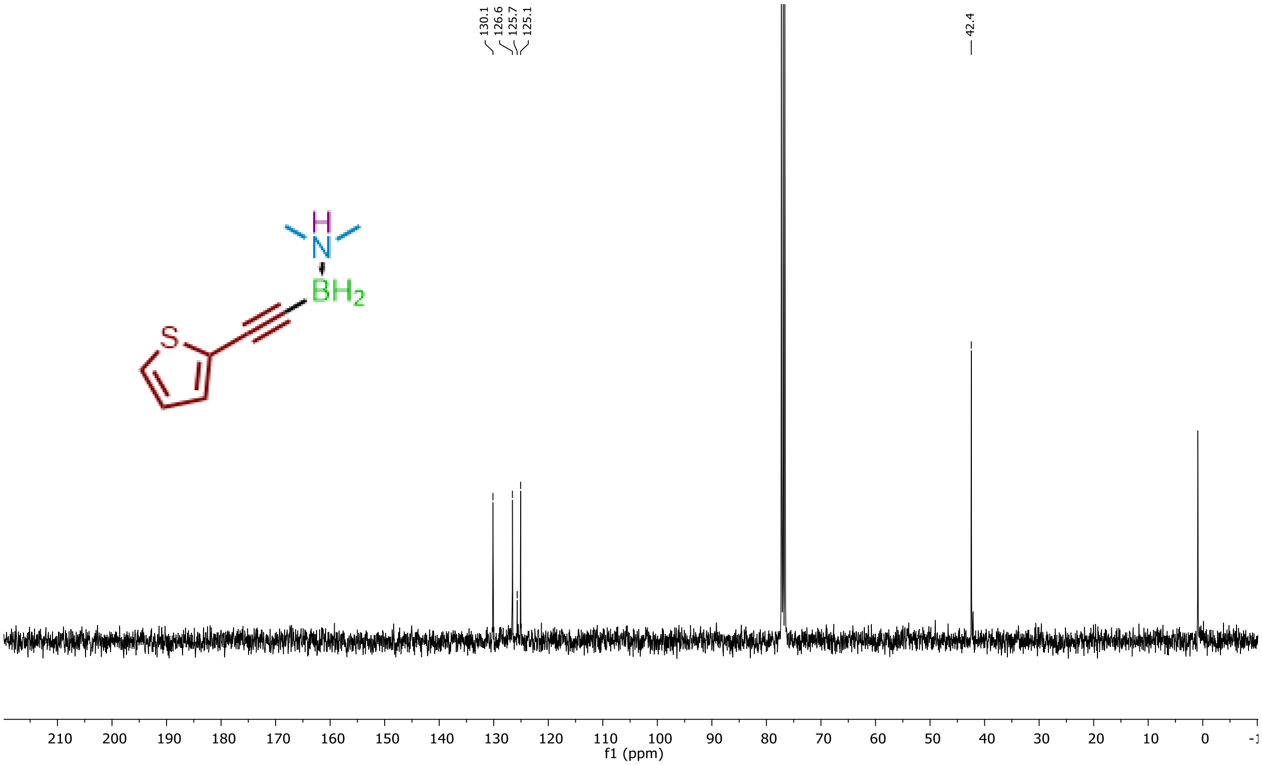

^{13}C NMR (75 MHz, $CDCl_3$) (thiophen-2-ylethynyl)borane-dimethylamine (**7ce**)

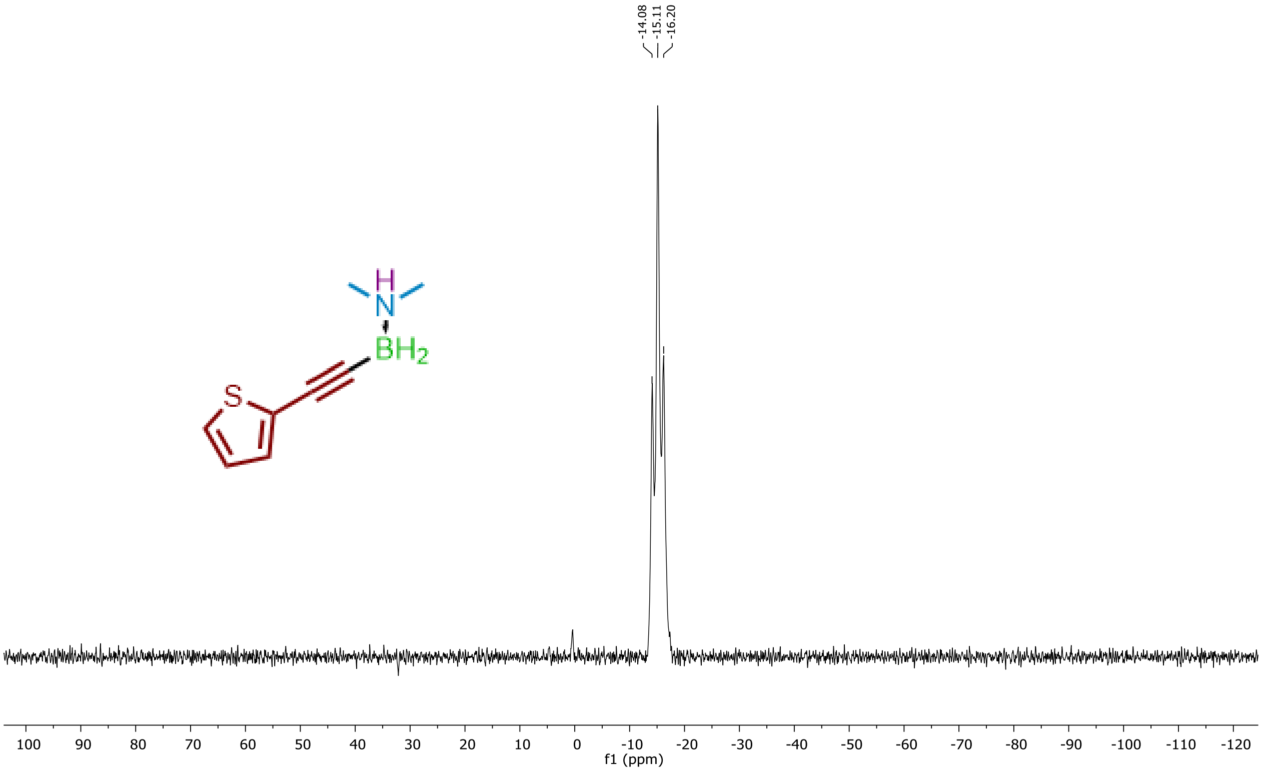

^{11}B NMR (96 MHz, $CDCl_3$) (thiophen-2-ylethynyl)borane-dimethylamine (**7ce**)

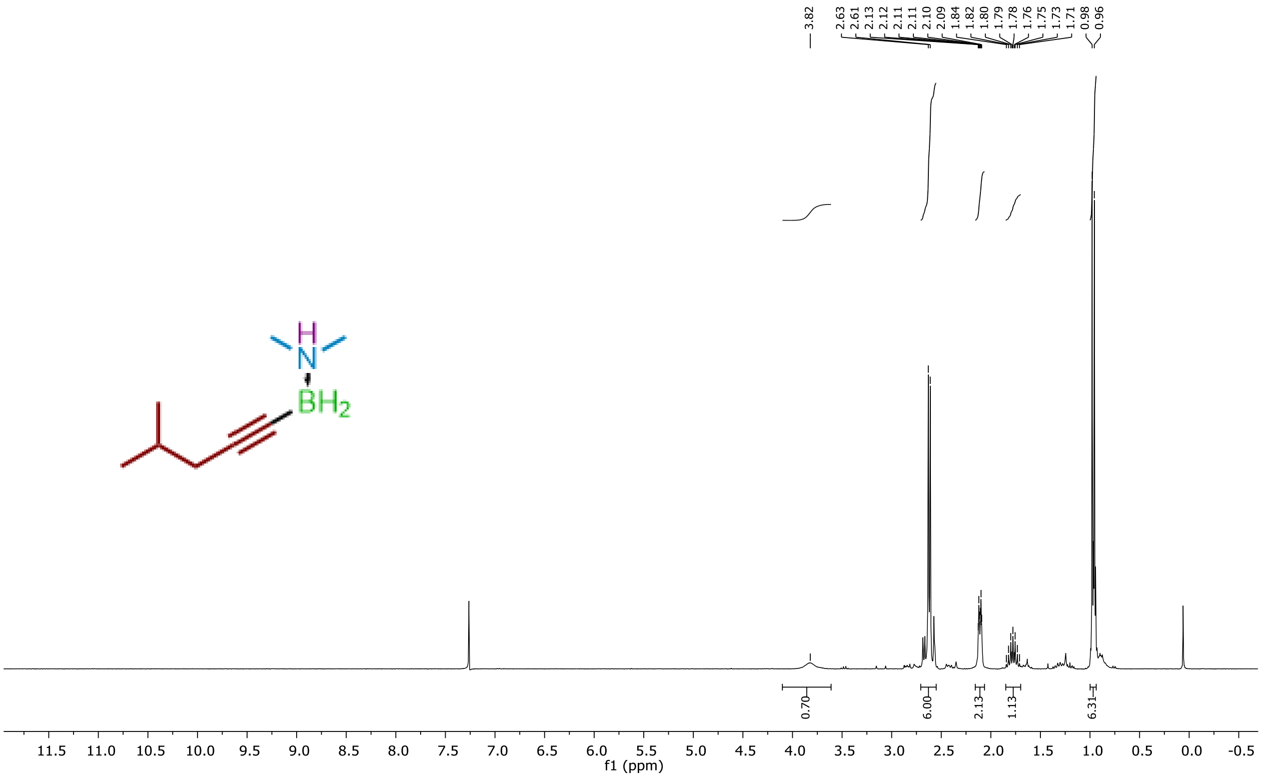

^{1}H NMR (300 MHz, $CDCl_3$) (4-methylpent-1-yn-1-yl)borane-dimethylamine (**7de**)

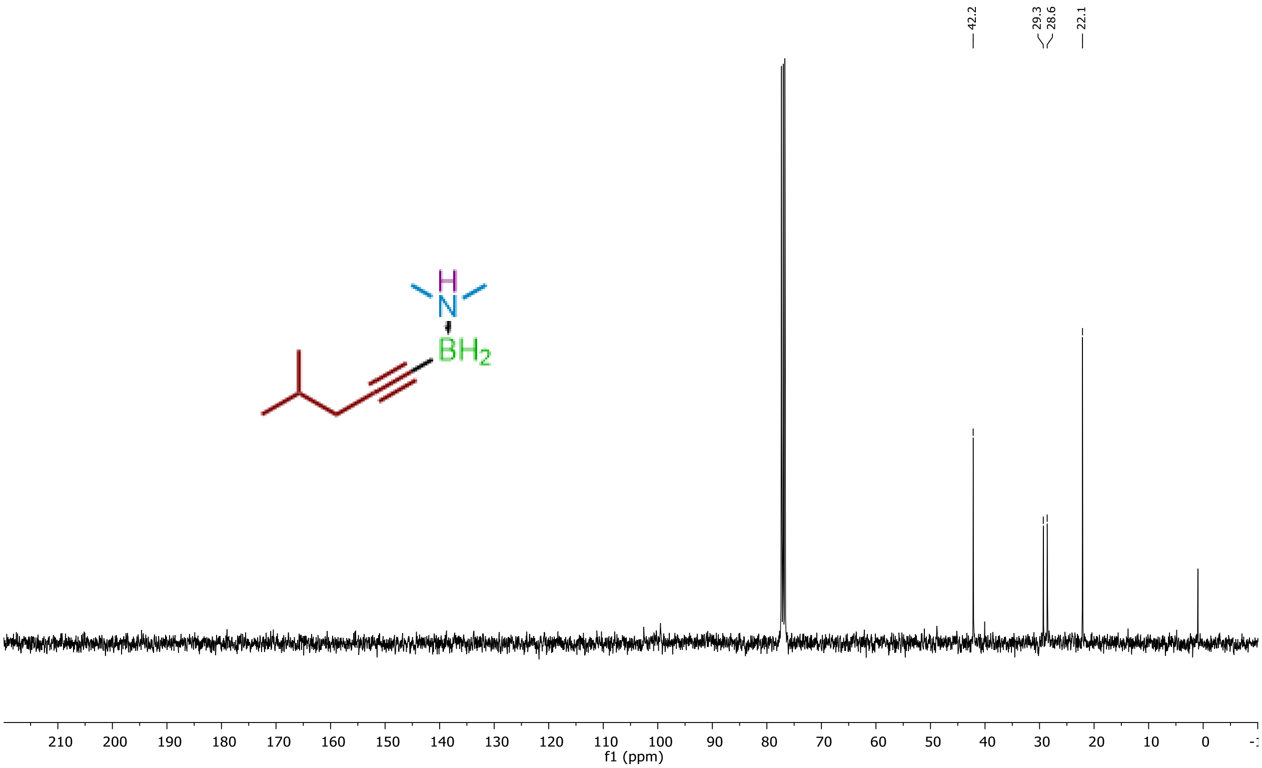

^{13}C NMR (75 MHz, $CDCl_3$) (4-methylpent-1-yn-1-yl)borane-dimethylamine (**7de**)

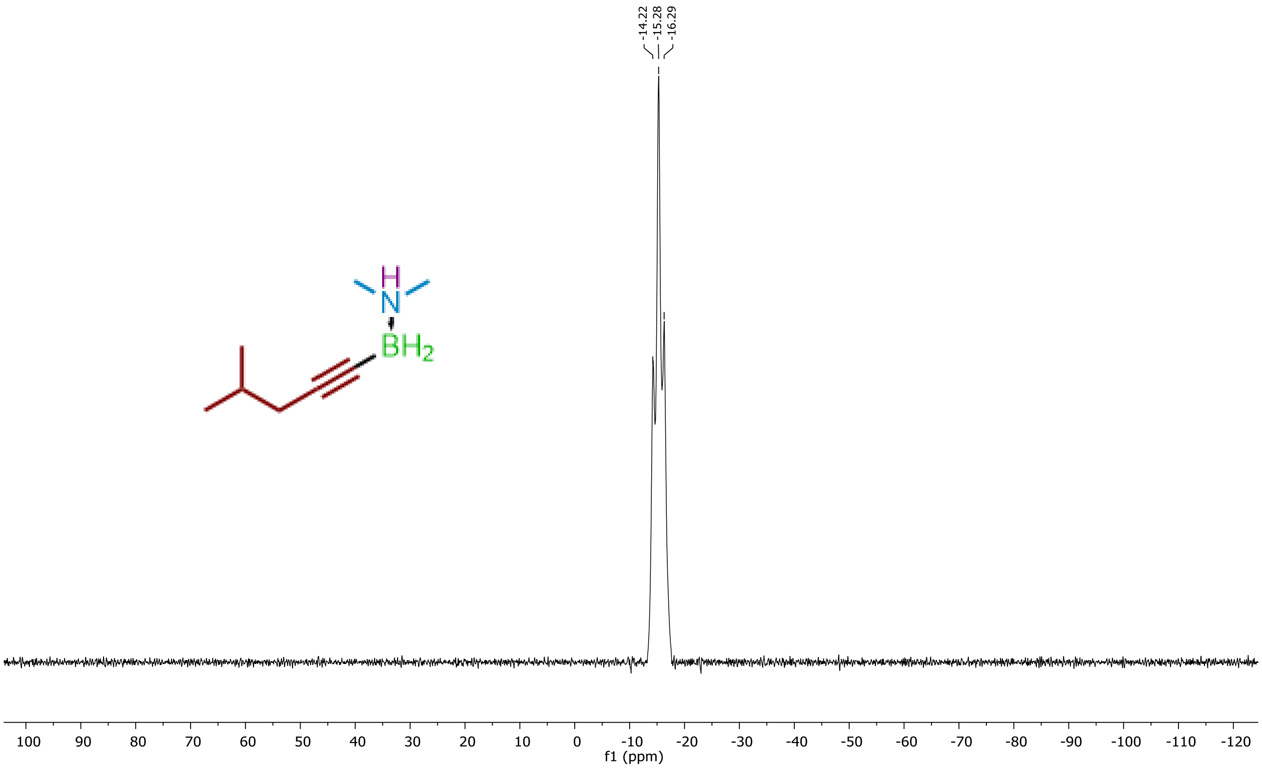

^{11}B NMR (96 MHz, $CDCl_3$) (4-methylpent-1-yn-1-yl)borane-dimethylamine (**7de**)
